# A systematic review of prolonged posture monitoring with accelerometer-based wearable sensors

**DOI:** 10.1371/journal.pdig.0001538

**Published:** 2026-09-15

**Authors:** Meixing Liao, Kai Huang, Eva Libeert, Bart Vanrumste, Simon Brumagne, Zhe Chen, Jean-Marie Aerts

**Affiliations:** 1 Department of Electrical Engineering, KU Leuven, Leuven, Belgium; 2 Department of Biosystems, KU Leuven, Leuven, Belgium; 3 Department of Rehabilitation Sciences, KU Leuven, Leuven, Belgium; 4 School of Economics and Management, Beihang University, Beijing, China; 5 Department of Rehabilitation, School of Nursing and Medical Technology, University of Medicine and Pharmacy at Ho Chi Minh City, Ho Chi Minh City, Vietnam; The University of British Columbia, CANADA

## Abstract

Prolonged sitting and standing are associated with health risks. Unlike generic sedentary behaviour monitoring based on energy expenditure, prolonged posture monitoring requires posture-specific detection and uninterrupted bout segmentation, yet current accelerometer-based approaches remain heterogeneous. This systematic review synthesises current practices and identifies research gaps across the sensing-to-feedback pipeline, including data collection, preparation, analysis, and user feedback. Ten databases, including Web of Science, Scopus, ProQuest Central, PubMed/MEDLINE, EMBASE, CINAHL, SPORTDiscus, IEEE Xplore, ACM Digital Library, and Engineering Village, were searched. Eligible studies monitored uninterrupted sitting or standing bouts of at least ten minutes during wake time using body-worn accelerometer-based sensors, provided sufficient methodological detail, and were published as peer-reviewed journal articles or full conference proceedings in English. From 27,334 records, fourteen studies were included. Risk of bias was assessed using design-specific tools: JBI checklists for randomised and cross-sectional studies, and WEAR-BOT for wearable-sensor validation or algorithm-development studies. Prolonged sitting was the primary target, while prolonged standing was not assessed as a dedicated bout-level outcome. Thigh-worn activPAL devices were most frequently used for posture measurement or as reference criteria. Bout duration thresholds and interruption rules varied substantially. Deep learning models reported the strongest performance for sitting recognition and transition detection. Four studies included feedback components, but none used real-time posture monitoring outcomes to trigger feedback. Substantial heterogeneity precluded quantitative synthesis, and the evidence base was concentrated in Western, office-based populations. Overall, accelerometer-based wearable sensors provide a feasible basis for monitoring prolonged sitting. Key gaps include inconsistent definitions, limited specific detection, heterogeneous data preparation and validation reporting, and a lack of translation from monitoring to actionable feedback. Future research should prioritise transparent bout definitions, reproducible pipelines, inclusive external validation, and interpretable feedback systems. PROSPERO registration: CRD42025637387.

## Author summary

Many people spend most of their day sitting or standing, which increases health risks. Commercial fitness trackers and existing research usually count total sitting time and measure inactivity, but few focus on how long specific postures are held uninterrupted. In this study, we systematically reviewed fourteen research articles on the monitoring of prolonged postures using wearable accelerometers. We examined sensor selection, data processing, analysis methods, and user feedback. Most research focuses on prolonged sitting. Few studies investigate prolonged standing as a separate issue. Only a few studies gave user feedback. None used real-time posture detection to trigger feedback. Our findings show that wearable sensors can monitor prolonged sitting. However, the varied methods across studies make comparison difficult. The field lacks consistent definitions of prolonged postures, detailed reporting of data preparation and analysis approaches, and further actions to turn monitoring into meaningful feedback. Inclusive research and method validation with diverse populations is needed before these tools can support posture and spinal health in daily life.

## Introduction

### Background: Sedentary behaviour and prolonged postures

For health and well-being, the World Health Organization (WHO) recommends that adults limit sedentary time and replace it with physical activity of any intensity [1]. Despite these recommendations, sedentary behaviour accounts for a substantial proportion of their waking hours. A recent accelerometer-based systematic review found that adult workers accumulated an average of 9.22 hours of sedentary behaviour per day [2]. Globally, insufficient physical activity remains highly prevalent, with nearly 1.8 billion adults (31% of the global adult population) failing to meet the recommended levels in 2022 [3].

The Sedentary Behaviour Research Network (SBRN) defines sedentary behaviour as any waking behaviour characterised by an energy expenditure ≤1.5 metabolic equivalents while in a sitting, reclining, or lying posture [4]. Most of the evidence on health effects concerns total sedentary behaviour or the accumulation of sedentary bouts rather than posture-specific monitoring. Nevertheless, bout duration and interruption patterns are increasingly recognised as relevant dimensions of sedentary exposure [5,6]. Additionally, prolonged standing has been associated with musculoskeletal symptoms and fatigue, although the evidence base is more limited and context-specific than that for the broader sedentary behaviour literature [7,8]. These findings highlight the importance of monitoring uninterrupted sitting and standing bouts alongside total sedentary time.

Although the importance of uninterrupted sitting and standing bouts is well recognised, the literature remains heterogeneous in its definitions and measurements of prolonged posture. Studies have used different duration thresholds to characterise prolonged sitting or standing, ranging from 5 minutes to 10, 20, 30, and 60 minutes, depending on the study objectives and applications [5,6,8–12]. In addition, studies differ in the sensors used (e.g., hip-, waist-, or thigh-worn accelerometers), wear protocols, and analytical procedures applied to detect posture and quantify bout accumulation [9,13–15]. Therefore, a comprehensive synthesis of current methodological approaches is needed to guide future research.

Given the wide range of duration thresholds, to maximise the inclusion of relevant studies, this systematic review operationally defines prolonged posture as a sitting or standing bout lasting at least 10 minutes without interruption during wake time. Although some studies have used shorter thresholds, such as 5 minutes [9], 10 minutes is considered more appropriate for representing prolonged posture in the context of this review. The focus is on sitting and standing, since they are most directly associated with occupational and daily musculoskeletal exposures. Prolonged lying or reclining postures during sleep are excluded, since distinguishing in-bed sedentary behaviour from sleep presents distinct physiological and methodological challenges [16].

### Digital monitoring with wearable sensors: Trends, gaps, and opportunities

Recent advances in wearable sensors, particularly accelerometer-based devices, offer a promising and affordable solution for non-invasive, continuous posture monitoring in everyday settings. With progress in signal processing and analysis, the latest devices collect movement and orientation data in real time, enabling posture detection and, in some cases, user feedback. For example, accelerometer-based systems have been deployed in occupational settings to track sedentary behaviour, while consumer wearable sensors, such as Fitbit, Garmin, and Apple Watch, integrate accelerometers for posture-related feedback during activities of daily life [17]. Together, these applications demonstrate the potential of wearable sensors to support dynamic posture monitoring and just-in-time alerts for prolonged posture interventions.

Despite these technological developments, current approaches remain methodologically diverse. As noted previously, studies differ in the sensor selection, placement, wear protocols, data preparation, and analysis methods used to quantify prolonged posture. These inconsistencies limit reproducibility, comparability, and clinical application. Commercial wearable sensors mainly provide general activity metrics, such as step count and total time spent sitting or standing. In contrast, detailed posture-specific and bout-level analyses usually require raw data from research-grade accelerometers with proper placement and sampling rates [18].

Moreover, given the ubiquity of smartphones and wearable devices, prolonged posture monitoring presents opportunities for mobile health interventions [19]. While this review focuses on monitoring, feedback features offer valuable insights for developing closed-loop or intervention-oriented systems that use prolonged posture monitoring data.

### Previous reviews and unaddressed problems

Several systematic reviews have investigated the use of wearable sensors for posture monitoring and physical activity assessment, but none directly focus on the methodological pipeline for detecting prolonged postures. Kristoffersson & Lindén reviewed wearable sensors for monitoring physical activity in the healthcare domain, such as gait, balance, and rehabilitation. However, their work did not specifically address prolonged postures [20]. Similarly, Kappattanavar et al. conducted a systematic review on the classification of sitting postures using inertial measurement units, accelerometers, and pressure sensors, but their focus was limited to identifying various sitting postures, with minimal discussion of sensor data collection and processing techniques [21]. Qi et al. explored physical activity recognition and monitoring within an Internet of Things (IoT) -based framework, highlighting sensor modalities, data processing methods, and machine learning (ML) applications. However, their review focused mainly on movement recognition rather than prolonged posture monitoring and did not evaluate real-time feedback mechanisms for musculoskeletal health interventions [22]. In the workplace, Khakurel et al. reviewed wearable applications for employee productivity and health monitoring, acknowledging their potential but focusing on work efficiency rather than the musculoskeletal risks associated with prolonged sitting [23].

Collectively, these existing reviews provide valuable insights into movement detection but do not offer a comprehensive analysis of prolonged posture monitoring. Notably, to the best of our knowledge, none of the previous reviews has synthesised the full sensing-to-feedback pipeline, from data collection and preparation to analytical methods and, where applicable, feedback-related features. These gaps motivate the need for a comprehensive, technical review of accelerometer-based prolonged posture monitoring.

### Objectives of this review

To address these gaps, this interdisciplinary systematic review aims to provide a comprehensive overview of accelerometer-based wearable sensors for prolonged posture monitoring, answering the main research question: **What are the current practices and techniques for prolonged posture monitoring using accelerometer-based wearable sensors?**

Recognising the complexity of the sensing-to-feedback pipeline, we structure this review around four interconnected sub-questions, each corresponding to a critical stage of the pipeline:

1. **Data collection:** What accelerometer-based wearable sensors are used for prolonged posture monitoring, and how is prolonged posture monitored in existing studies?
2. **Data preparation:** What techniques are applied to prepare raw accelerometer data for analysis?
3. **Data analysis:** What data analysis methods are used to monitor prolonged postures?
4. **User feedback strategies:** What feedback strategies, if any, are implemented to inform users about prolonged posture?

**Fig 1:**
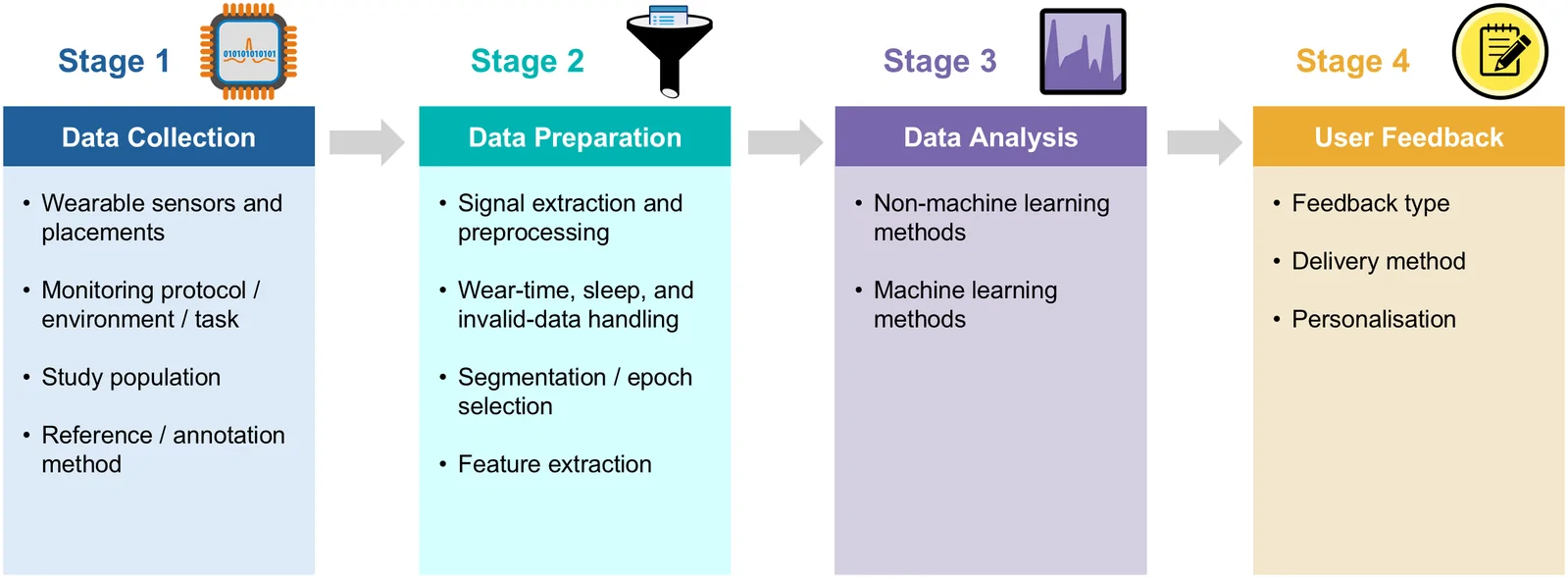
An overview of the sensing-to-feedback pipeline for accelerometer-based prolonged posture monitoring. The figure summarises key methodological stages and sources of variation identified across the reviewed studies, spanning data collection, data preparation, data analysis, and user feedback. The icons in this figure were obtained from Openclipart, which are released under the Creative Commons Zero 1.0 Public Domain License (stage 1: https://openclipart.org/100999, stage 2: https://openclipart.org/305652, stage 3: https://openclipart.org/200086, stage 4: https://openclipart.org/279779). The figure was assembled and modified by the authors.

As shown in Fig 1, these four stages reflect the sequential flow from sensor selection to actionable user feedback, where decisions made in earlier stages influence possibilities and outcomes at later stages. By addressing these four sub-questions in accordance with the Preferred Reporting Items for Systematic Reviews and Meta-Analyses (PRISMA) guidelines [24,25], this review aims to bridge disciplines and highlight the potential of accelerometer-based systems for prolonged posture monitoring.

Additionally, these sub-questions reflect critical design considerations for effective digital health solutions that aim to reduce prolonged static postures and promote proactive posture change. By synergising current practices across the entire sensing-to-feedback pipeline, this review provides actionable insights for researchers, clinicians, and digital health developers interested in prolonged posture monitoring and intervention through wearable technology.

## Materials and methods

### Protocol registration

This systematic review was conducted according to the PRISMA 2020 guidelines [24,25], and the completed PRISMA checklist is provided in S1 Checklist. The review protocol is registered with the International Prospective Register of Systematic Reviews (PROSPERO) under ID CRD42025637387. The initial registration was submitted on February 5, 2025, and last updated on June 17, 2026 [26]. Updates were made to align with the manuscript revisions, and the update notes are accessible in the PROSPERO registration. The most recent protocol registration (version 6.0) is provided in S1 Protocol.

### Information source and search strategy

A comprehensive search strategy was developed in collaboration with biomedical librarians. The search strategy combined three core concepts using Boolean operators: (1) wearable accelerometer-based sensors, (2) prolonged sitting or standing postures, and (3) data analysis methods. We included both free-text terms (e.g., “accelerometer”, “sedentary”, “machine learning”) and controlled vocabularies (e.g., Medical Subject Headings [MeSH]), with search terms adapted to each database. The search strategy prioritised terminology related to wake-time posture monitoring and sedentary behaviour, reflecting how prolonged postures are predominantly indexed in the wearable sensing literature.

The initial literature retrieval was conducted on January 12, 2025, in ten databases: Web of Science (SCI-EXPANDED, SSCI, AHCI, CPCI-S, CPCI-SSH, ESCI), Scopus, ProQuest Central, PubMed (including MEDLINE), EMBASE, CINAHL, SPORTDiscus, IEEE Xplore, ACM Full-text Collection, and Engineering Village (EI). To enhance search sensitivity, we refined the search strategy during the update search in December 2025. Specifically, we included behaviour and posture-related terminology defined by SBRN (“sedentary bout” and “standing bout”) and terms commonly used in posture monitoring literature (e.g., “sitting bout”, “uninterrupted sitting”, “sit to stand”) [4,9,27]. Furthermore, we identified device-specific terms from the wearable sensor literature and preliminary search results, such as “activPAL”, “ActiGraph”, “Axivity”, “GENEActiv”, “Movesense”, and “movisens”.

The refined search strategy was re-executed on December 4, 2025, across all databases except EI, as its results were duplicated in the other nine databases. To ensure completeness and maintain a timely review, an additional catch-up search was conducted on April 8, 2026, across all ten databases used in the initial search in January 2025, including EI. The search terms remained the same as in December 2025 to maintain consistency, while the Boolean logic was standardised: first, combine the title, abstract, and keywords for each concept using “OR”, then combine the three concepts using “AND”. Complete database-specific search strategies, modifications in each round, and retrieval counts are provided in S1 File.

All records retrieved in each search round were screened using the same eligibility criteria described in the following subsection. No language or publication date restrictions were applied during database searching. Articles published in languages other than English were excluded during title and abstract screening. In addition, reference lists of relevant reviews and included studies were screened to identify potentially eligible articles.

### Eligibility criteria

The following section outlines the inclusion and exclusion criteria applied in this review.

#### Inclusion criteria.

Studies were included if they met all of the following conditions:

1. Monitor prolonged sitting and/or standing bouts

The study focused on or reported the monitoring of prolonged sitting and/or standing bouts during wake time. For the purposes of this review, prolonged posture is defined as uninterrupted sitting and/or standing bouts lasting at least 10 minutes.

2. Use of accelerometer-based wearable sensors

Eligible studies must use at least one body-worn accelerometer-based sensor as the primary data collection tool. Smartphone-based studies are eligible if the phone is firmly worn on the body and its built-in accelerometer is used for posture detection.

3. Sufficient methodological reporting

The study provided adequate detail to allow interpretation of prolonged posture monitoring, which, at a minimum, includes: (i) population; (ii) sensor type and anatomical placement; (iii) measurement protocol, such as wear duration and tasks; and (iv) methods used to derive prolonged posture outcomes.

4. Population and publication
  4.1. Population: Conducted with human participants.
  4.2. Language: Full-text articles published in English.
  4.3. Publication type: Original peer-reviewed journal articles or peer-reviewed full-paper conference proceedings (published, early access, and in press).

#### Exclusion criteria.

Studies that did not meet all the inclusion criteria were excluded. The following are examples of exclusion:

1. Out-of-scope focus, including studies that monitored only bouts shorter than 10 minutes, studies of sedentary behaviour or physical activity without specified postures, posture classification studies lacking prolonged bouts, and sleep studies.
2. Ineligible instrumentation, defined as posture detection relying on non-wearable or non-accelerometer-based methods.
3. Insufficient methodological detail on how the prolonged postures were monitored.
4. Ineligible population or publication, including non-human studies, non-English full texts, inaccessible full texts, and publications that were not peer-reviewed or otherwise did not meet eligibility requirements.

### Selection process

After deduplication in EndNote Web, all identified records were imported into Rayyan. All screening was conducted by reviewers with interdisciplinary backgrounds and expertise in posture monitoring using wearable technologies. M.L. holds a background in mathematics, data science, and IoT. K.H. has a background in automation and human factors, with a research focus on work-related musculoskeletal disorders using ML. E.L. is a human health engineer.

In the title/abstract screening phase of the initial search in January, 2025, two reviewers (M.L. and K.H.) independently screened each record blindly against the eligibility criteria, and the disagreements were resolved through discussion. Records newly identified through the updated search on December 4, 2025, were screened by one reviewer (M.L.) using the same eligibility criteria and procedures as the initial search. Records identified through the catch-up search on April 8, 2026, were screened by the same reviewer using the revised eligibility criteria reported in this manuscript, the main update being the explicit definition of prolonged posture. To ensure rigour and consistency, in both update search and catch-up search, a second reviewer (K.H.) independently screened all records flagged for full-text retrieval or marked as uncertain, as well as a random 20% sample of records excluded at the title/abstract stage. Title/abstract screening was not repeated in full, as studies referring to prolonged posture or sedentary behaviour monitored using wearable sensors had already been retained at that stage, and the later refinement of the eligibility criteria did not alter title/abstract screening decisions.

Full-text articles of potentially relevant studies were retrieved and assessed for inclusion. To ensure consistency with the latest eligible criteria, all articles carried forward to full-text screening across all three searches were re-screened independently by both reviewers using the final inclusion/exclusion criteria reported in this manuscript, regardless of their results in the previous two searches. Both reviewers (M.L. and K.H.) independently assessed them for inclusion. Any disagreements (coded as “include” vs. “exclude” or “unsure”) were resolved through discussion between the two primary reviewers. If consensus could not be reached, the tiebreaker (E.L.) made the final decision. The study selection process was documented using a PRISMA flow diagram. Studies excluded during full-text screening are reported in the Results section.

### Data extraction

#### Data collection process.

After testing in a subset of included studies, a standardised data extraction form was developed and refined to ensure clarity and consistency. Two reviewers (M.L. and K.H.) independently extracted data from each study. Discrepancies were resolved through discussion and consensus.

#### Data items.

The data items extracted for all studies are demonstrated in Table 1.

**Table 1:** Extracted data items.

Category	Data items
General information	Index, first author and year, study type, primary objective, main conclusions, reporting items (code/data/algorithm availability, protocol/trial registration), identified search round (initial round in January 2025, updated round in December 2025 or catch-up round in April 2026), and data extraction date.
Prolonged posture characteristics and reported outcomes	Reported prolonged postures (sitting, standing, or both), definition of prolonged posture, interruption rule/tolerance if reported, and reported prolonged posture outcomes (e.g., number of prolonged bouts, bout duration, total prolonged sitting/standing time, longest bout, break frequency).
Stage 1: Data collection	Population characteristics (sample size, age, gender, health status), measurement environment, sensor type/model and placement, sensor role (test measure or reference), reference criterion/ground truth, wear protocol, measurement tasks, and sampling rate.
Stage 2: Data preparation	Signal extraction and preprocessing, wear-time, sleep, and invalid-data handling, segmentation or epoch selection, and feature extraction.
Stage 3: Data analysis	Analytical approach, posture classification logic, bout detection approach, main results, validation method, and timing (real-time/post-hoc).
Stage 4: User feedback (if reported)	Type of feedback provided, delivery method, and personalisation.

### Study risk of bias assessment

Given the heterogeneity of study designs, quality and risk of bias were assessed with design-specific tools. Method development and validation studies were evaluated using the WEArable Technology Risk of Bias and Objectivity Tool (WEAR-BOT) validity checklist [28]. Observational studies were appraised with the Joanna Briggs Institute (JBI) checklist for analytical cross-sectional studies [29], and intervention studies were assessed with the JBI critical appraisal checklist for randomised controlled trials [30]. Two reviewers (M.L. and K.H.) conducted assessments independently and resolved disagreements through discussion. Assessment results informed the interpretation of findings and were summarised narratively to address methodological heterogeneity. No formal certainty assessment was conducted. This review synthesised methodological practices rather than intervention outcomes or pooled effect estimates.

### Synthesis methods

All studies were included in the synthesis if they met the predefined inclusion criteria. Studies lacking key details (e.g., missing outcome measures, unclear methodologies) were excluded from the synthesis. The data extracted were tabulated (S1 Dataset) by two independent researchers (M.L. and K.H.), cross-checked, and summarised. The synthesis was organised to align with the sensing-to-feedback pipeline: data collection, preparation, analysis, and user feedback (Fig 1). Due to methodological heterogeneity across studies, no quantitative synthesis or meta-analysis was conducted. Instead, a structured narrative synthesis was performed, with visual representations (e.g., tables and figures) used to summarise trends in sensor placement, device types, analysis techniques, and feedback strategies.

## Results

### Study selection

**Fig 2:**
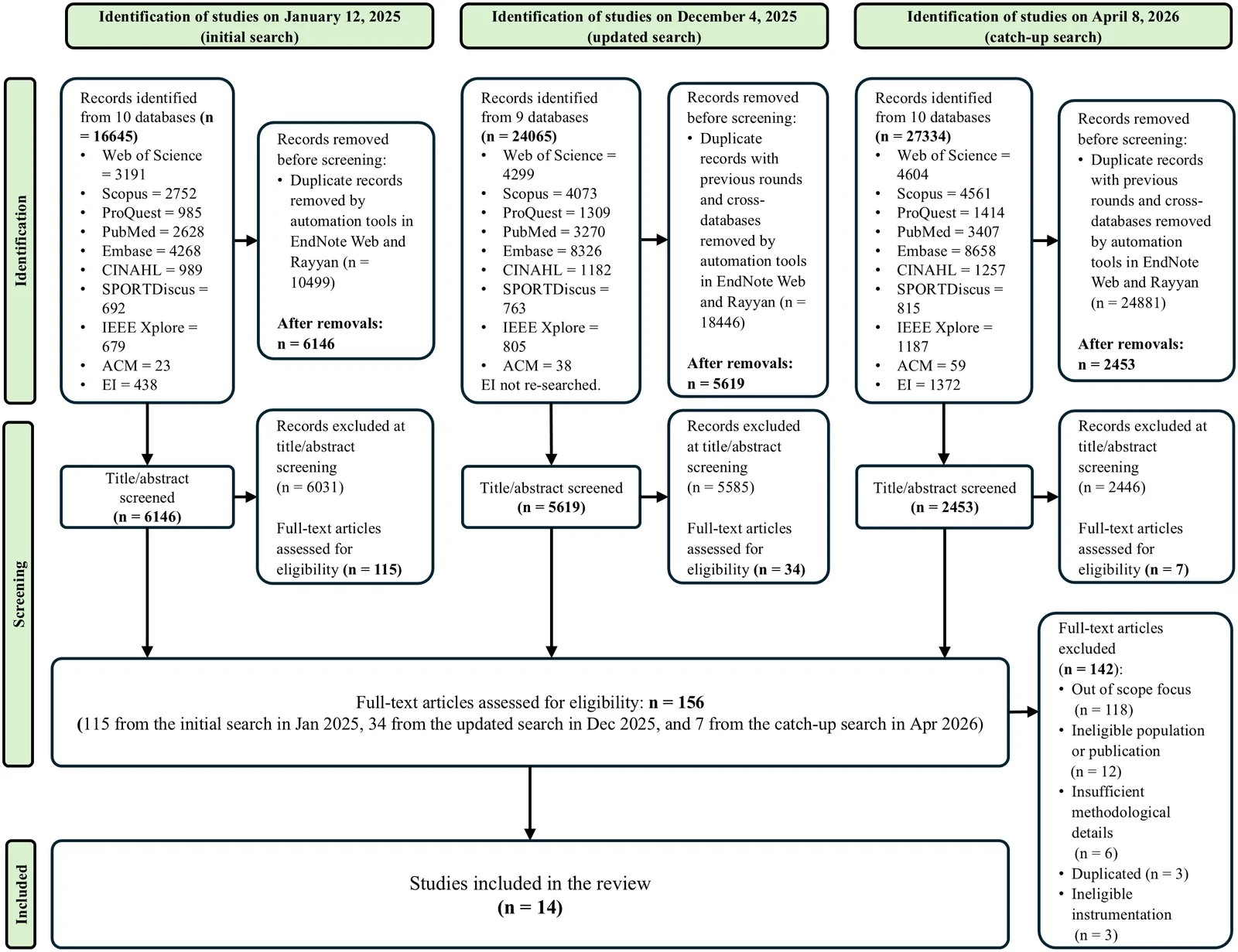
PRISMA flowchart of the results from the literature search.

Fig 2 provides an overview of the number of articles identified, screened, and excluded at each stage. Of 27,334 articles extracted from databases using the specified search strategies (S1 File), fourteen papers were ultimately included in this review [9,13–15,27,31–39].

Full-text exclusions were conducted according to the eligibility criteria outlined in the Methods section for the following primary reasons:

- Duplicate records (n = 3);
- Ineligible instrumentation (n = 3);
- Ineligible population or publication type (n = 12);
- Insufficient methodological detail (n = 6);
- Out-of-scope study focus, including
  - Sedentary behaviour studies without posture monitoring or prolonged bout reporting (n = 78);
  - Activity recognition or posture classification without prolonged posture monitoring (n = 17);
  - Sedentary behaviour studies that reported prolonged sedentary bouts but did not separate sitting/lying postures (n = 8);
  - Studies addressing sitting or standing bouts shorter than 10 minutes (n = 8);
  - Non-wear time detection (n = 3);
  - Posture correction (n = 2);
  - Descriptive posture allocation study (n = 1);
  - Sitting discomfort prediction (n = 1).

A comprehensive list of excluded studies, along with the rationale for exclusion, is available in S1 Table.

### Study characteristics

S1 Dataset provides the detailed data extraction form covering the items listed in Table 1. S1 Fig gives a visual summary of methodological characteristics across the sensing-to-feedback pipeline in the fourteen included studies.

Across the included studies, the earliest was published in 2012 [38]. Notably, most studies were published from 2018 onward [9,13–15,27,31–34,36,37,39], indicating increased interests in recent years. All included studies focused exclusively on prolonged sitting, with none specifically reporting outcomes related to prolonged standing. The most frequently used threshold for defining a prolonged bout was 30 minutes (n = 8, 57.1%) [13,15,27,31,33,35,37,38]. Most studies were conducted in unrestricted free-living settings (n = 11, 78.6%) [9,13,15,27,31–37], with additional studies in occupational free-living contexts (n = 6, 42.9%) [14,27,31,34,35,38], and one in an educational free-living context [39]. The majority of the study samples comprised healthy adults aged 18–65 years [9,14,27,31–38], while older adults, patients, children, or adolescents were less commonly represented.

Methodologically, the reviewed literature mainly chose 3-axis accelerometers (n = 13, 92.9%) [9,13–15,27,31–37,39], with activPAL being the most frequently used sensor (n = 12, 85.7%) [9,13–15,27,31,33–38]. Self-report diaries or logs served as the primary reference or contextual notes (n = 11, 78.6%) [9,14,15,27,31,33–36,38,39], and most studies used wear protocols of at least seven days (n = 12, 85.7%) [9,13–15,27,31–37]. Sampling rates varied, with 30 Hz being the most common (n = 5, 35.7%) [9,13,15,33,37]. For data preparation, both proprietary/validated processing tools and custom preprocessing were used in 11 studies (78.6%) [9,13–15,27,31–39]. Diary-based wear-time handling was also reported in 11 studies (78.6%) [9,14,15,27,31,33–36,38,39], while algorithm-based handling was used in eight studies (57.1%) [9,13,15,27,32,33,36,37]. Event-based segmentation and fixed-window epoching were equally prevalent (n = 6 each, 42.9%) [13,15,27,31–39]. Outcome-based features represented the most common feature extraction strategy (n = 8, 57.1%) [14,27,31,34–36,38,39]. At the analytical stage, non-ML approaches were slightly more common than ML methods (n = 8, 57.1% versus n = 6, 42.9%), and all studies conducted post-hoc rather than real-time analyses. Only four studies (28.6%) incorporated user feedback [27,31,35,38]. Reporting of reproducibility-related items was limited: original code was available online in four studies (28.6%) [9,13,15,33], datasets were available upon request in six (42.9%) [13,14,27,31,32,39], and published protocols or trial registration were reported in five (35.7%) [27,31,34,35,38]. The included studies were evenly distributed among interventional studies (n = 4, 28.6%) [27,31,35,38], method development and validation studies (n = 5, 35.7%) [9,13,15,33,37], and observational or feasibility studies (n = 5, 35.7%) [14,32,34,36,39]. The following subsections provide a detailed report of the results.

### Prolonged posture characteristics and reported outcomes

As detailed in S1 Dataset (“Prolonged posture” sheet), the included studies demonstrated considerable variation in the definition and operationalisation of prolonged posture. As visualised in S1 Fig, in all included studies, sitting was the primary posture targeted for prolonged monitoring, and none reported outcomes specifically related to prolonged standing bouts.

Nevertheless, six studies reported total daily and/or occupational standing time without performing bout-level analyses [9,27,34–37]. In two intervention studies, standing was promoted as the recommended behaviour to interrupt prolonged sitting, but standing-related outcomes were not reported [31,38]. Four studies reported either sit-to-stand transition frequency or detection accuracy [13,15,37,39]. However, transition counts do not provide information about the duration of subsequent standing bouts or whether standing was accumulated in prolonged uninterrupted periods. One study uniquely examined temporal patterns of “time on feet”, a composite category that included standing still, standing with small movements, walking, running, climbing stairs, and cycling. This study reported the percentage of work time spent in long bouts (»30 minutes) of time on feet, but did not distinguish between static standing and ambulation [14].

The threshold for defining a prolonged bout varied across studies, with ≥ 30 minutes of uninterrupted sitting being the most frequently used criterion, appearing in eight studies [13,15,27,31,33,35,37,38]. Other thresholds included ≥ 5 and ≥ 10 minutes [9], ≥ 20 minutes [31,37], and ≥ 60 minutes [27,36]. Three studies did not specify exact thresholds for prolonged bouts but instead reported multiple bout duration categories [32,34,39]. Notably, one study categorised uninterrupted bouts for both sitting and time on feet into bins of: < 1, 1–2, 2–3, 3–4, 4–5, 5–10, 10–30, 30–60, and >60 minutes, and further grouped them as short (≤ 5 minutes), moderate (>5 to ≤ 30 minutes), and long (>30 minutes) [14].

Event-based aggregation of consecutive posture classifications from wearable devices was the standard analytical approach across studies [9,27,31,35,36,38]. Two studies required strictly uninterrupted bouts, in which any non-sitting or non-sedentary epoch ended the bout [13,33]. One study applied a minimum duration of 30 seconds to define a sedentary bout or break [32]. Another study allowed a limited timing exception for work-hour assignment, including sedentary periods that crossed the start or end of the reported workday if the majority of the bout occurred within work hours and no more than 10 minutes extended outside [38]. A 1-minute lag tolerance for transition pairing was also reported, although consecutive sedentary minutes were still aggregated into strict sitting bouts [15].

Reported prolonged posture-related outcomes demonstrated considerable variability in studies. The most commonly assessed outcome was the total time accumulated in prolonged sitting bouts [9,13,15,27,31,33–37]. In addition to total time, several studies characterised the distribution of bout lengths by reporting either the number of prolonged bouts per day [27,31,38], or the number and accumulated time across multiple duration categories [32,34,37,39]. Derived pattern metrics, such as mean bout duration, usual bout duration, and the alpha coefficient, which describes the steepness of the bout length distribution, were also recorded [13,15,32,33]. Postural transition metrics were similarly documented, including the number of breaks in sedentary time [13,32–34], sit-to-stand transition frequency [13,15,37,39], and time between sitting bouts [35]. Some studies categorised outcomes of prolonged sitting monitoring by context, reporting separately for work hours, non-work hours, weekdays, and weekends [27,34,35]. Lastly, one study applied isometric log-ratio coordinates to compare time on feet and sitting across short-, moderate-, and long-bout categories [14].

Due to substantial differences in wear protocols, bout thresholds, and units reported across studies, absolute outcome values could not be pooled quantitatively. Results are therefore presented narratively using representative examples. Where absolute values were reported, the burden of prolonged sitting was considerable, though it varied across thresholds and contexts. For example, in an office-based intervention study, participants spent 5.7 ± 1.0 hours per day sitting at work, with 3.3 ± 1.3 hours per day accumulated in bouts of at least 30 minutes [38]. Similarly, another office worker study found that prolonged sitting in bouts of 20–30 minutes accounted for 49.4 ± 19.3% and 36.0 ± 17.9% of work time, respectively [31]. In a large population-based cohort, average sitting time was 9.5 hours per day in men and 9.0 hours per day in women, with 2.4 hours per day and 2.2 hours per day, respectively, accumulated in bouts of at least 60 minutes [36]. In contrast, some studies emphasised temporal patterning rather than a single prolonged threshold. For example, one free-living study reported 290.8 minutes per day of sitting, a 50% weighted median sitting bout of 17.4 minutes, 37.9 sitting bouts per day, and 42.6 breaks per day [32]. Collectively, these examples demonstrate that the included studies assessed different dimensions of prolonged posture, such as total exposure, prolonged bout burden, bout distribution, and interruption frequency, thereby limiting direct comparisons across studies.

### Data collection

The first stage of the prolonged posture monitoring pipeline is data collection. This section examines accelerometer-based wearable sensors, detailing the study population, measurement environments, sensor type and model, sensor placements, sensor functions, methods for establishing reference criteria, wear protocols, measurement tasks, and sampling rates. The detailed extracted data are presented in S1 Dataset (“Data collection” sheet).

#### Study populations and measurement environments.

The 14 extracted studies included more than 7,400 participants monitored across various free-living settings. Sample sizes in small-scale validation studies ranged from 21 to 38 participants [9,31,32,37,38], while a large cohort investigation involved more than 5,000 individuals [36].

Several studies monitored participants in unrestricted free-living conditions, capturing posture during typical daily activities without domain-specific limitations [15,32,33,36,37]. In contrast, a substantial subset focused on context-specific free-living environments, where monitoring occurred under naturalistic conditions within defined settings such as offices, workplaces, or schools [9,14,27,31,34,35,38,39]. These settings included commercial banking [31], insurance company offices [27], university workplaces [38], eldercare work [14], and primary school classrooms [39].

The majority of studies investigated healthy, working-age adults monitored during unrestricted daily life [9,14,27,31–35,38]. Two cohort studies assessed middle-aged adults from general populations in free-living conditions [32,36]. Clinical and chronic disease populations were represented in two studies: one examined office workers with and without type 2 diabetes [34], and another focused on female breast cancer survivors [37]. Three studies included older adults aged above 65 [15,33,37], while two targeted primary school children [13,39].

Notably, all included studies were conducted exclusively in high-income Western countries. Three studies originated from the United Kingdom [31,36,38], and three from Australia [13,33,35]. Two studies each were conducted in Sweden [9,14], the United States [15,37], and Finland [32,39]. Single studies were based in Spain [34], and the Netherlands [27].

Taken together, the included studies show that prolonged sitting monitoring has been examined in diverse populations, but mainly in adult free-living and occupational contexts in developed countries.

#### Accelerometer-based wearable sensors: type, placement, and role.

A wide range of accelerometer-based wearable sensors was used across the included studies, each requiring a different wear protocol and sensor configuration.

**Fig 3:**
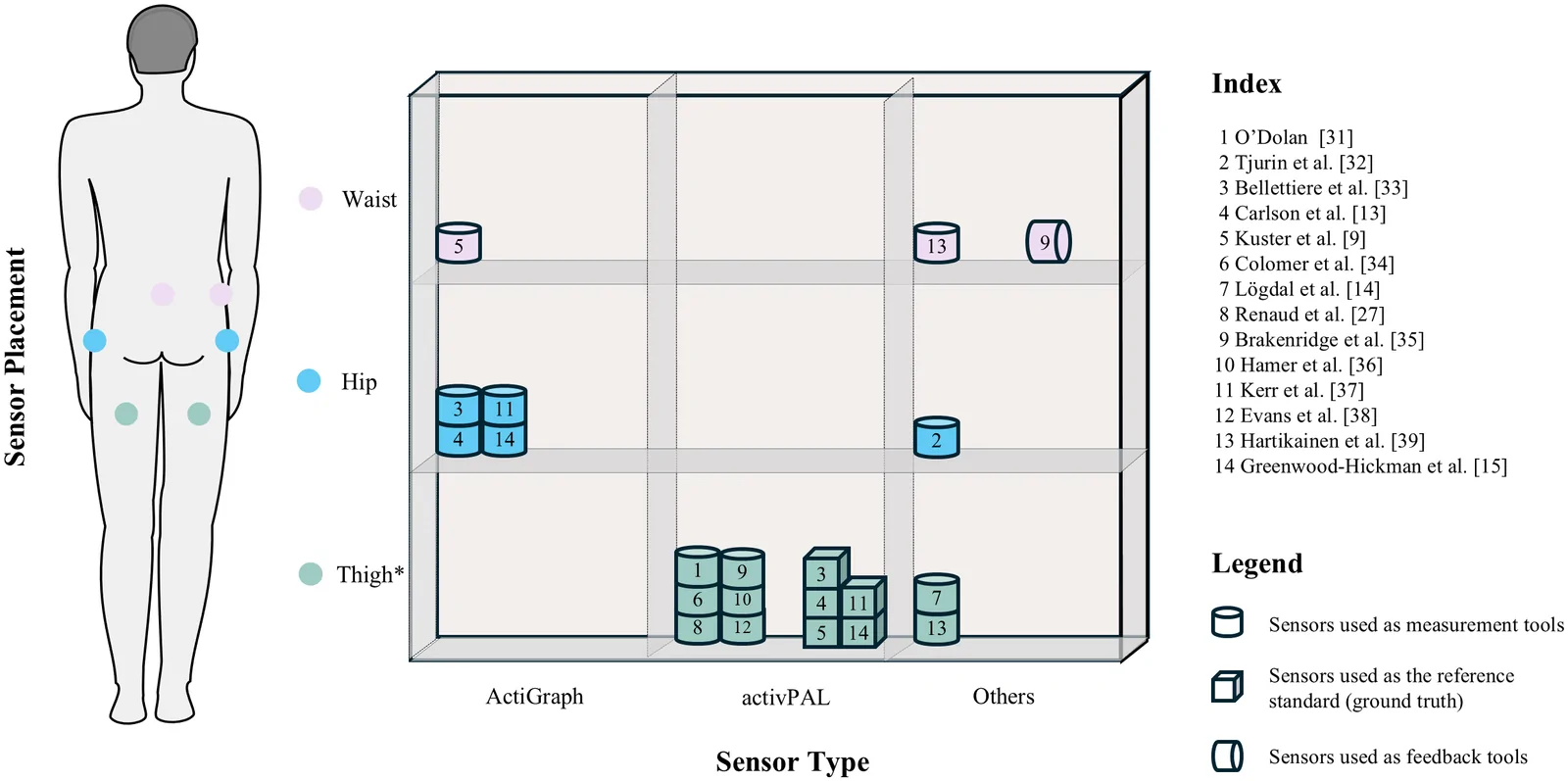
Distribution of studies by sensor placement and sensor type. The index corresponds to the index presented in the data extraction form in S1 Dataset. Numbers in brackets following author names indicate reference numbers. In the visualisation, sensor placement is indicated by colour, sensor type by layering, and sensor role by shape. The legend specifies that vertical cylinders represent sensors used as measurement tools, cubes indicate sensors used as reference standards (ground truth), and horizontal cylinders represent sensors used as feedback tools. Thigh-mounted sensors* are located at the midline of the anterior aspect of the thigh. The body silhouette in this figure was obtained from Openclipart, which is released under the Creative Commons Zero 1.0 Public Domain License (https://openclipart.org/14572). The figure was assembled and modified by the authors.

The most frequently used sensor was activPAL (n = 11, 78.6%). Among them, six studies employed activPAL as the primary objective measure [27,31,34–36,38], while five used it as the reference criterion for validating hip- or waist-worn accelerometers [9,13,15,33,37]. ActiGraph devices represented the second most common sensor type (n = 5, 35.7%), appearing exclusively in method development and validation studies [13,15,33,37]. These lightweight accelerometers were typically worn at the hip, with one study using a waist placement [9]. Other sensors were used in single studies, including the Hookie AM20 hip-worn triaxial accelerometer [32], the Axivity AX3 thigh-worn accelerometer [14], and the RM42 triaxial accelerometer, which was worn at the waist and thigh in school children [39]. Additionally, one study incorporated a waist-worn consumer tracker (LUMOback) for real-time feedback alongside an activPAL reference measure [35]. Fig 3 presents the distribution of studies by sensor placement and sensor type.

As demonstrated, sensor placement varied according to study objectives and device design. The thigh was the most frequently used placement (n = 13, 92.9%), reflecting the predominance of activPAL and the use of Axivity and RM42 thigh-worn sensors [9,13–15,27,31,33–39]. The hip ranked second (n = 5, 35.7%), corresponding to studies using ActiGraph and Hookie AM20 devices [13,15,32,33,37]. Three studies examined waist-worn sensors, including ActiGraph [9], RM42 [39], and LUMOback [35]. Six studies implemented dual-sensor configurations, combining a thigh-worn reference device with a hip- or waist-worn test device for algorithm validation or feedback [9,13,15,33,37,39].

#### Reference criterion.

In the included studies, the primary reference criterion included self-reported diaries, logs, or schedules. These instruments identified contextual periods, such as waking hours, bedtime, work hours, sensor removal or non-wear time [9,14,15,27,31,33–36,38,39]. Their primary function was to refine the analysis window and improve the interpretation of free-living data, rather than to serve as an independent posture reference.

Additionally, ten studies used activPAL-derived event-level or algorithm-based postural classification [9,13,15,27,33–38]. In these investigations, activPAL functioned as the primary ground truth for labelling sitting/lying, standing, and stepping events. Several studies specifically used activPAL event files or posture labels as the criterion for training, testing, or validating alternative classifiers [9,13,15,37]. Furthermore, one study relied on ActiPass, a previously validated classification algorithm, alongside diary data [14], while another incorporated a thigh-worn accelerometer and a validated mean amplitude deviation method, supplemented by visual inspection and school schedule information [39]. One study explicitly stated that activity data were cross-validated against completed diaries as the primary reference, rather than an independent device-based criterion [31]. Another study did not report a reference criterion [32].

In general, in the reviewed studies, thigh-worn activPAL most frequently served as the reference criterion, while hip- or waist-worn accelerometers, particularly ActiGraph devices, were assessed as alternative measurement tools. As quantitative performance was influenced by the entire analytical pipeline rather than sensor hardware alone, comparative accuracy across populations and settings is presented in the Data analysis section.

#### Wear protocol and measurement tasks.

Among the 14 studies reviewed, short-term monitoring in free-living settings was the predominant design. Most studies implemented a 7-day continuous wear protocol in free-living contexts, instructing participants to wear the sensor(s) continuously, including during sleep and, in some cases, during water-based activities [14,15,31,33–37]. One study implemented a 9-day protocol but excluded the initial attachment and final detachment days, resulting in 7 full days of analysable data [27]. Other notable deviations included a 14-day waking-hours protocol [32], a median 9-day wear period [9], a school-week protocol [39], a 5-workday protocol [38], and a paediatric study that monitored up to 8 consecutive days per season over four seasons [13].

A further distinction was the extent of monitoring, specifically whether whole-day activities or context-specific activities were assessed. Two studies limited monitoring to specific contexts rather than conducting a full free-living assessment. One study collected workplace-only data over five workdays during both the baseline and intervention periods [38], while another restricted measurements to general education classroom lessons over a single school week, excluding physical education and recess [39]. In dual-sensor studies using both thigh- and hip-worn devices, participants were typically required to wear the activPAL reference device for 24 hours, whereas the ActiGraph was worn only during waking hours [33,37]. Participant diaries were frequently used to document wake time, bedtime, working hours, and device removal periods, facilitating accurate identification of waking wear time and differentiation between occupational and non-occupational contexts [9,14,27,31,34,35,38,39]. Minimum valid wear criteria varied across studies, with some requiring at least four valid days with a minimum of 600 minutes of daily wear time [32], or at least 12 hours of recording per day [9]. Across all studies, measurement tasks consisted of unrestricted free-living daily activities during the designated wear periods, with no prescribed structured activities.

#### Sampling rate selection.

As indicated in S1 Fig, the included studies demonstrate considerable variation in the reported sampling rates during data collection, with values ranging from 20 to 100 Hz.

Most studies reported sampling rates of 20 or 30 Hz for raw accelerometer data. Studies employed the default configurations of activPAL (20 Hz) [9,27,36], or ActiGraph devices (30 Hz) [9,13,15,33,37]. One used a frequency of 25 Hz with the Axivity AX3 sensor [14]. Two studies reported the highest sampling frequency of 100 Hz, using Hookie AM 20 and RM42 triaxial accelerometers, respectively [32,39]. Several articles did not explicitly report the sampling frequency [31,34,35,38], and one study referenced only the use of activPAL default settings without providing a numeric value [27].

### Data preparation

The second stage of the prolonged posture monitoring pipeline is data preparation, during which raw accelerometer signals are processed into structured inputs suitable for analysis. The reviewed studies used diverse data preparation techniques that varied according to sensor type, monitoring protocols, and analytical objectives. For systematic comparison, these procedures were categorised into four primary components: 1) signal extraction and preprocessing; 2) handling of wear-time, sleep, and invalid data; 3) segmentation or epoching; and 4) feature extraction. This framework aligns with the main data preparation practices documented in accelerometer-based posture and activity monitoring research [40–43].

#### Signal extraction and preprocessing.

The included studies used a combination of proprietary manufacturer software, custom analytical scripts, and validated external processing pipelines to extract and prepare accelerometer data.

Several studies used proprietary software packages or validated processing tools for initial signal extraction and event classification [9,14,27,31,33–38]. In addition, multiple ML studies input raw ActiGraph triaxial acceleration directly into models, occasionally incorporating activPAL event files as reference labels [13,15,33,37].

To address advanced analytical objectives and synchronise multi-sensor data streams, several studies implemented preprocessing strategies based on custom analytical scripts in Matlab [14,32,34], Excel [34,38], SAS [35], HSC analyse programme (for occupational data) [27], or freely available validated software tools [36]. Two studies downsampled raw ActiGraph triaxial data from 30 to 10 Hz using boxcar aggregation to reduce the dataset size and standardise inputs for subsequent ML pipelines [13,15]. Another study processed raw, unfiltered hip accelerometer data to classify five postural categories, applying a low-pass filter with a 0.5 Hz cutoff to estimate the average gravity direction [37]. One study processed data in a supercomputing cluster, calculating the mean amplitude deviation over 1-second non-overlapping epochs [44], and further averaging over 15-second intervals to capture short activity bursts, with visual inspection of accelerometer traces to verify lesson-specific wear time [39]. The most comprehensive preprocessing pipeline synchronised the clocks of ActiGraph and activPAL devices by estimating time offsets from cross-correlations of normalised sensor axes across consecutive 3-hour episodes, fitting a linear approximation of drift over time, and correcting the ActiGraph timestamps accordingly. This pipeline also incorporated valid-recording-time selection, sleep removal, and exclusion of non-wear and contradictory signals [9].

In general, signal extraction and preprocessing approaches ranged from standardised software-based event extraction to highly customised raw signal pipelines that included downsampling, filtering, synchronisation, and behavioural relabelling. This methodological heterogeneity demonstrates that, even before feature extraction or modelling, the included studies exhibited substantial differences in the preparation of posture-relevant signals for subsequent analysis.

#### Wear-time, sleep, and invalid-data handling.

The handling of wear, sleep, and invalid data showed substantial methodological variation in the included studies. In general, three main strategies include: 1) self-reported diaries/logs; 2) algorithmic or threshold-based detection of non-wear and sleep; and 3) study-specific validity screening rules.

Self-reported diaries or logs were used in eleven of the 14 studies (78.6%) to document bedtime, wake time, work hours, device removal, or inconsistencies between reported and recorded wear [9,14,15,31,33–35,38,39]. In some cases, these records were used only to define wake or work periods, whereas in others they were cross-checked against monitor outputs and used to exclude inconsistent or invalid data [9,15,31,33,39].

Algorithmic or threshold-based approaches to wear-time handling were reported in eight studies [9,13,15,27,32,33,36,37,45,46]. Four studies implemented the Choi algorithm for ActiGraph data, using a 90-minute window, a 30-minute stream frame, and a 2-minute tolerance to identify non-wear periods [13,15,33,37,45,46]. Two studies used ProcessingPAL or similar automated activPAL-based routines to detect sleep, prolonged non-wear, and invalid data, such as the Winkler et al. automated sleep/non-wear algorithm within ProcessingPAL [13,27,47]. One study defined non-wear as at least 30 consecutive minutes of zero values, with the first and last minutes of each wear period trimmed to minimise dressing and undressing artefacts. In this study, time exceeding 20 hours of daily use was also excluded from lying time accumulated after midnight, when participants were assumed to be asleep [32]. A large cohort study used a custom algorithm to classify sleep or non-wear based on the longest bout per 24-hour period, extended bouts, and adjacent low-movement windows [36]. Additionally, a study excluded ActiGraph non-wear using diary information, sensor discrepancies, and periods of at least 90 minutes of constant raw signal, which were interpreted as prolonged non-wear [9].

The validation screening criteria exhibited considerable heterogeneity. Four dual-monitor studies restricted their analysis to valid concurrent waking wear of both devices, excluding periods when either monitor was not worn or when the participants were in bed [13,15,33,37]. Three studies used visual inspection to verify wear periods or to identify invalid recordings, such as device time drift or unconfirmed classroom wear [15,33,39]. The minimum inclusion thresholds varied: at least one valid day with 10 hours of waking wear after excluding the first partial day [36]; at least one workday with four hours of recording [14]; at least two workdays with a minimum of four hours in each measurement period [38]; or all days with less than 95% of time spent in mode activPAL code, at least 500 steps, and at least 12 hours of recording [9]. In an occupational study, activPAL data were considered valid only if wear time exceeded 80% of self-reported work hours, and days with fewer than 500 steps, at least 95% of the time in any activity, or a maximum of 10 hours of waking wear were considered invalid [27].

Overall, wear time, sleep, and invalid data handling were highly study-specific, reflecting differences in device type, monitoring context, and study design. The most common pattern was a combination of diary-assisted annotation, algorithmic non-wear detection, and additional validity screening, but no standardised approach was observed across the included studies.

#### Segmentation and epoching.

Segmentation strategies in included studies can be categorised into four main approaches: event-based processing (n = 6), fixed-window epoching (n = 6), minute-based classification (n = 1), and continuous bout summarisation (n = 1).

Event-based segmentation was used primarily in studies that relied on activPAL output or other posture classification pipelines that directly detected posture changes, rather than using a fixed epoch length. In these six studies, posture events such as sitting/lying, standing, stepping, or walking were first identified and subsequently summarised into bout-based prolonged posture outcomes [27,31,34–36,38]. In addition, a study grouped event-based posture output into bout durations of <20, 20–40, 40–60, and >60 minutes [34]. Another study kept sedentary periods that crossed reported work boundaries if most of the event occurred within work hours and no more than 10 minutes were extended beyond them [38].

Fixed-window epoching was also implemented in six studies [13,15,32,33,37,39], with substantial variation in epoch lengths. Epochs ranged from 1-second preprocessing intervals to 15-second summaries, as well as 5- or 10-second model-input windows. Two studies used 5-second windows for feature extraction and classification [32,37]. Three studies within the Convolutional Neural Network (CNN) Hip Accelereometer Posture (CHAP) method framework chose 10-second non-overlapping windows as the primary unit of analysis [13,15,33]. In the CHAP-child study, activPAL one-second labels were aggregated into 10-second epochs, and an epoch was classified as sedentary if at least 6 of the 10 seconds were labelled sitting/lying. Additionally, one study processed the waist-worn signal in non-overlapping 1-second epochs and then averaged over 15-second intervals, while the thigh-worn device employed a separate transition-detection algorithm [39].

One study implemented minute-based posture classification rather than short, fixed windows. Although ActiGraph episode data were available at a 1-second resolution, the prediction model classified posture at the minute level. For model development, only training minutes with constant activPAL labels were included, while testing minutes comprised all available data from days with at least 10 hours of recording [9]. Another study summarised continuous behaviour data using exposure variation analysis rather than a conventional classification epoch. In this investigation, bout durations were initially categorised into nine bins (<1, 1–2, 2–3, 3–4, 4–5, 5–10, 10–30, 30–60 and >60 minutes) and subsequently merged into three broader categories: short (≤ 5 minutes), moderate (>5 to ≤30 minutes), and long (>30 minutes) bouts [14].

In summary, segmentation strategies ranged from event-driven posture outputs to fixed windows of 1, 5, 10, 15 seconds, and 1 minute, with event-based and short-window approaches equally prevalent.

#### Feature extraction.

Another important step in accelerometer-based posture analysis is extracting features from sensor data, which defines how raw sensor signals are transformed into meaningful inputs for subsequent analysis. In this review, we categorise the extracted features by their methods of derivation, i.e., whether they were manually computed, derived from processing outcomes, or automatically learnt by models. Consequently, based on the common patterns observed in the included studies and the evidence, we organise the feature extraction methods into three categories: manually engineered features, outcome-based features, and automatically learnt features [48,49].

The first category, manually engineered features, was used in three of the 14 studies (21.4%) [9,32,37]. These features were explicitly computed from raw accelerometer signals using predefined statistical, signal processing, or biomechanical formulas, and subsequently served as inputs to classical ML models. The extracted features included:

- Time-domain features, such as mean, minimum, maximum, zero-crossing rate, mean amplitude deviation, standard deviation, percentiles, and vector-magnitude summaries [9,32,37].
- Frequency-domain features, such as dominant frequency, band power, power-spectrum measures, and entropy, were also reported [9,37].
- Orientation-related features, such as roll, pitch, yaw, gravity-orientation angles, and low-pass-filtered angular measures, were used to capture posture-relevant information [9,37].

The most comprehensive handcrafted pipeline computed 563 candidate features per training minute, consisting of 409 time-domain and 154 frequency-domain features. These were reduced to a final subset using random forest relevance ranking, followed by sequential forward selection. The best performing bout detection model ultimately used 14 features [9].

The second and most common category, outcome-based or derived features, was used in eight of the 14 studies (57.1%) [14,27,31,34–36,38,39]. These features were not extracted directly from raw sensor windows but were derived from intermediate posture classifications, processed behavioural summaries, or threshold-based measures. Common examples include event-level posture labels produced by activPAL or similar processing software, such as sitting/lying, standing, stepping, or walking, which were then aggregated into bout-based prolonged posture outcomes [27,31,34–36,38]. Additionally, some studies applied more abstract derived representations, such as on-feet/sitting bout distributions transformed into five orthogonal isometric log-ratio coordinates for compositional analysis [14], or mean amplitude deviation with a sedentary threshold of 16.7 mg combined with thigh-derived sit-to-stand transitions standardised to 60 minutes of classroom time [39]. Other studies used derived activity outputs, such as cadence thresholds or validated count-based thresholds, to identify intensity-related categories [50]. For example, moderate-to-vigorous physical activity was defined using a step cadence of at least 100 steps per minute [34,36]. These features represent a higher level of abstraction and were used primarily in observational, intervention, and epidemiological analyses rather than in model training.

The final category, automatically learnt features, was selected by three studies [13,15,33]. In these studies, features were extracted directly from raw accelerometer data using ML models without manual feature engineering. All three studies implemented a CNN with Bidirectional Long Short-Term Memory (CNN-BiLSTM) architectures to capture spatial and temporal patterns from raw tri-axial signals. Within these architectures, the CNN functioned as an automatic feature extractor, while the recurrent layer modelled sequential transitions between sitting and non-sitting states. This end-to-end learning strategy was applied in the CHAP framework and its adult and child extensions, enabling models to learn posture-relevant features directly from raw acceleration data.

In general, non-ML studies relied mainly on processed posture outcomes or derived behavioural summaries. Classical ML studies used handcrafted multivariate feature sets, while deep learning studies automatically incorporated feature extraction within the model.

### Data analysis

This section focuses on data analysis, the third phase of the prolonged posture monitoring pipeline. In this review, we define data analysis as the modelling strategies used to detect, assess, classify, or predict prolonged postures, and validate the accuracy of accelerometer-based monitoring systems. The reviewed studies used multiple rule-based thresholds, statistical methods, and ML techniques, with varying model complexity, data requirements, interpretability, and real-time applicability. To facilitate comparison, we organise these approaches into two broad categories based on whether they rely on ML-based modelling.

#### Non-machine learning methods.

Eight of the 14 included studies (57.1%) applied non-ML analytical approaches. Instead of developing ML models, these studies treated accelerometer-derived prolonged posture variables as study outcomes and analysed them using conventional statistical or rule-based methods. These methods included t-tests, Analysis of Covariance (ANCOVA), generalised linear models, linear mixed models, principal component analysis (PCA), exposure variation analysis (EVA), and compositional data analysis (CoDA) [14,27,31,34–36,38,39].

In workplace intervention studies, these methods were primarily used to evaluate whether prompts, organisational support, or workstation modifications affected outcomes related to prolonged sitting monitoring. A randomised trial that used paired t-tests and ANCOVA demonstrated that point-of-choice prompts reduced the number of prolonged sitting events by 0.14 events per hour (*p* = 0.012) and their duration by 15.4% (*p* = 0.007) compared to education alone. However, total sitting time did not differ significantly between groups (4.4%, *p* = 0.084) [38]. A cluster-randomised feasibility study, analysed with independent t-tests and effect sizes, indicated a tendency for the prompt-plus-education group to spend less time in prolonged sitting than the education-only group. Nevertheless, the 95% confidence intervals (CI) were wide, and the changes did not persist at follow-up. At baseline, prolonged sitting during work represented 49.4% ± 19.3% of time in events longer than 20 minutes and 36.0% ± 17.9% in events longer than 30 minutes. From baseline to intervention, both groups reduced prolonged sitting, but reductions were greater in the education-only group than in the prompt-plus-education group for percentage of work hours sitting (-6.5% vs 2.4%), mean sitting-event duration (-4.0 vs -0.9 minutes), time in events longer than 20 minutes (-12.8% vs -6.0%), and time in events longer than 30 minutes (-12.0% vs -4.2%) [31]. The two larger workplace trials also used mixed-model approaches, but reached different conclusions. Linear mixed models with multiple imputation indicated that both intervention arms improved sitting-related outcomes over time, but only the tracker arm demonstrated significant advantages between-groups at 12 months for overall stepping time (+20.6 minutes per 16-hour day, 95% CI: 3.1, 38.1, *p* = 0.021) and step count (+846.5 steps per 16-hour day, 95% CI: 67.8, 1625.2, *p* = 0.033) [35]. In contrast, another study using linear and logistic mixed models found no significant differences in the primary outcome, i.e., total sitting time, at the 8-month follow-up [27].

Observational studies used non-ML analytical methods to characterise behavioural patterns across various contexts and populations. For example, one study applied three-way Analysis of Variance (ANOVA) and one-way ANCOVA, revealing that students in open learning spaces exhibited 1.8 more 1–4-minute sedentary bouts per hour (*p* < 0.001), fewer bouts that exceeded 10 minutes (0.20 vs 0.48 bouts/h, *p* = 0.004), and 0.9 more sit-to-stand transitions per hour (*p* = 0.009) compared to students in conventional classrooms [39]. Another investigation applied PCA to identify multivariate patterns of sedentary behaviour among office workers with type 2 diabetes, finding that the first two main components accounted for approximately 60% of the variance and that adults with type 2 diabetes accumulated 31 fewer breaks per day than those without diabetes [34]. Additionally, descriptive statistics and generalised linear models demonstrated the feasibility of large-scale thigh-worn monitoring: 83.0% of fitted devices resulted in usable data, 79.6% of participants recorded at least six full valid days, and prolonged sitting in uninterrupted bouts of at least 60 minutes comprised 25.3% of total daily sitting in men and 24.4% in women [36]. Furthermore, one study expanded non-ML analysis beyond sitting-only metrics by examining both time spent on feet and time spent sitting among eldercare workers. Accelerometer outputs were initially summarised using event-based analysis, then transformed using CoDA into five orthogonal isometric log-ratio coordinates, and analysed using Multivariate Analysis of (co) Variance (MANOVA/MANCOVA), followed by univariate models. Home care and nursing home workers spent the majority of the workday on their feet (51.9% and 56.9%, respectively). The only significant difference between settings was that home care workers accumulated 30.1% less sitting in long bouts than in moderate or short bouts, compared to nursing home workers (*p* = 0.011). Increased age was also associated with more time on feet compared to sitting (*p* = 0.002, $\eta^{2}=0.06$) and more time on feet in long bouts compared to moderate and short bouts (*p* = 0.001, $\eta^{2}=0.06$) [14].

In summary, these eight non-ML studies demonstrated that accelerometer-derived prolonged posture outcomes can be effectively analysed using conventional statistical techniques to evaluate interventions, compare behavioural contexts, and characterise clinically relevant posture patterns. However, since these studies generally did not benchmark alternative detection methods, their primary contribution was to the interpretation and application of prolonged posture metrics rather than to direct comparisons of analytical performance.

#### Machine learning methods.

ML methods were used in six of the 14 included studies (42.9%) to classify posture, detect transitions, or estimate prolonged sitting bouts. Three studies applied classical ML models based on manually engineered features [9,32,37], while three applied deep learning CNN-BiLSTM models from the CHAP family that learnt representations directly from raw triaxial accelerometer signals [13,15,33]. Only these studies reported quantitative performance, which supported direct comparisons of monitoring accuracy across populations and settings. Table 2 summarises the study population, test sensor, reference criterion, analytical model, and the key quantitative performance metrics reported.

**Table 2:** Structured summary of machine learning studies by population, model, analytical task, and reported performance.

Paper	Population	Test sensor (placement)	Reference criterion	Model	Main task	Key reported performance
Tjurin et al. [32]	Middle-aged adults (n = 36)	Hookie AM20 triaxial accelerometer (hip)	No independent reference criterion reported	Bagged-trees classifier using 20 engineered features from raw 3D acceleration	Classify overall sedentary behaviour and sitting; derive bout-based variables	Prior validation of the model reported 96.4% sensitivity and 99.2% specificity for lying; and 92.2% sensitivity and 99.2% specificity for sitting using leave-one-out cross-validation. In the present study, the model was primarily used to derive sedentary and sitting pattern variables rather than to benchmark against alternative classifiers.
Kuster et al. [9]	Office workers (n = 38)	ActiGraph GT3X (waist)	activPAL (thigh)	Feature-selected ensemble tree-based classifier	Detect prolonged sitting bouts	The new algorithm predicted time in sitting bouts ≥5 min and ≥10 min with absolute bias ≤7 min/day. Among the ActiGraph cut-points, only 150 cpm without low-frequency extension did not differ significantly from activPAL for prolonged sitting (bias ≤18 min/day).
Kerr et al. [37]	Breast cancer survivors (n = 30)	Random forest classifier trained on activPAL-labelled hip accelerometer data	ActiGraph (hip)	activPAL (thigh)	Classify sitting, standing, stepping, sit-to-stand, and stand-to-sit	Accuracies were 77% for stepping, 63% for standing, 67% for sitting, 52% for sit-to-stand, and 51% for stand-to-sit. The 100 cpm method overestimated short sitting bouts and underestimated long sitting bouts.
Greenwood-Hickman et al. [15]	Older adults (n = 709)	ActiGraph GT3X+ (hip)	activPAL (thigh)	CNN-BiLSTM (CHAP)	Sitting/non-sitting classification, sit-to-stand transition detection, and sitting pattern estimation	Minute-level sitting/non-sitting agreement was 93%. Sensitivity for the sit-to-stand transition and PPV were both 83%. Mean sitting bout duration was 15.7 min for CHAP versus 15.4 min for activPAL.
Bellettiere et al. [33]	Adults aged 35+ (training n = 981, validation n = 421)	ActiGraph GT3X+ (hip)	activPAL (thigh)	CNN-BiLSTM (CHAP-Adult)	Sitting/non-sitting classification, transition detection, and sitting pattern estimation	Sensitivity was 95.3%, specificity was 89.8%, and positive predictive value (PPV) was 93.5%. Transition sensitivity was 74.4% and PPV was 77.6%. Correlations with activPAL ranged from 0.78 to 0.96; mean absolute percentage error (MAPE) was 4.0% for total sitting time, 12.2% for breaks, 11.6% for mean bout duration, and 9.6% for time in ≥30-min bouts.
Carlson et al. [13]	Children aged 8–11 years (n = 278)	ActiGraph GT3X+ (hip)	activPAL3 (thigh)	CNN-BiLSTM (CHAP-child)	Sitting/non-sitting classification, transition detection, and sedentary bout pattern estimation	Mean balanced accuracy was 87.6%. At a 1-min tolerance, transition sensitivity was 71.1% and was PPV 71.2%. For participant-season variables, most MAPEs were ≤11%, although time in ≥30-min bouts had 21.0% MAPE. The 100 cpm method showed markedly larger errors, with most MAPEs > 30%.

Comparative analyses indicate that performance varied substantially across populations and settings. Among office workers, an ActiGraph-based feature-selected ensemble classifier estimated time spent in sitting bouts of at least 5 and 10 minutes with an absolute bias of no more than 7 minutes per day relative to activPAL. In contrast, the best-performing conventional cut-point exhibited a bias of up to 18 minutes per day [9]. For breast cancer survivors, a hip-worn ActiGraph random forest classifier demonstrated moderate posture classification performance, with accuracies of 77% for stepping, 63% for standing, 67% for sitting, 52% for sit-to-stand, and 51% for stand-to-sit [37]. In older adults, CHAP achieved 93% agreement for minute-level sitting versus non-sitting classification, as well as 83% sensitivity and 83% positive predictive value (PPV) for sit-to-stand transitions [15]. Among adults aged 35 years and older, CHAP-Adult classified sitting with 95.3% sensitivity, 89.8% specificity, and 93.5% PPV. The correlations ranged from 0.78 to 0.96, and the mean absolute percentage error (MAPE) was 9.6% for time in bouts of at least 30 minutes [33]. In children, CHAP-child achieved a mean balanced accuracy of 87.6%, with sit-to-stand transition sensitivity and PPV of 71.1% and 71.2%, respectively. Most participant-season bout pattern variables remained within 11% MAPE of activPAL, although the time in bouts of at least 30 minutes showed a 21.0% MAPE [13]. These results indicate that the comparative accuracy depends on the sensor, placement, preprocessing, analytical model, and the population being monitored.

Overall, the classical ML studies demonstrated variable performance. Models based on engineered features were effective for specific adult applications, particularly when the analytical target was narrowly defined, such as estimating prolonged sitting bouts [9] and classifying sitting [32]. In contrast, performance decreased for tasks that involved multi-class posture and transition classification under free-living conditions, as shown in a random forest study of breast cancer survivors [37]. Another study reported strong prior validation for a bagged tree classifier, but the model was used primarily for descriptive purposes rather than to compare alternative approaches in free-living analyses [32].

In contrast, the three CHAP deep learning studies demonstrated consistently superior performance in the elderly, adults aged 35 years and older, and children [13,15,33]. Among older adults and general adult samples, CHAP-based models exhibited high agreement with activPAL for both sitting classification and sitting pattern estimation, achieved strong accuracy in transition detection, and produced relatively low error for bout-based variables [15,33]. Although performance in children remained robust, it was lower than in adult populations, particularly for longer bout estimates [13].

In summary, these findings indicate that analytical performance was influenced by both the modelling approach and the target population. Classical ML approaches were most effective for narrowly defined adult applications, whereas CHAP deep learning models delivered the most robust and consistent performance for simultaneous posture classification, transition detection, and posture pattern estimation across diverse age groups. Overall, deep learning demonstrated the strongest analytical performance among the evaluated models for prolonged posture monitoring.

### User feedback

Only four studies incorporated any form of user feedback strategy as the final stage of the pipeline [27,31,35,38], highlighting a gap in translating posture monitoring outcomes into actionable behaviour support.

In these four intervention studies, feedback was delivered through computer-based prompts, wearable activity trackers, or multi-component workplace programmes. Specifically, a study implemented hourly Microsoft Outlook meeting reminders to stand, featuring customised, positively framed messages that users could snooze or dismiss [31]. Another study used point-of-choice computer prompts that displayed a non-minimisable advice window for one minute every 30 minutes, accompanied by brief verbal and booklet-based education [38]. The third study provided a multi-component intervention that included optional haptic vibration feedback after 15 or 30 minutes of uninterrupted sitting through a waist-worn tracker, in addition to sit-stand workstations, group sessions, and a self-help booklet with personal goal setting [27]. The final study combined organisational support strategies with a waist-worn tracker that provides real-time feedback on sitting, standing, and stepping via a smartphone application, supplemented by champion-led emails and individualised activity reports [35].

The degree of personalisation varied across these four studies, ranging from fixed prompts [38], to moderate customisation of message content and user-controlled prompt intervals [27,31], and to workplace-branded communications incorporating participant images and quotes [35]. All feedback interventions focused exclusively on reducing prolonged sitting, without providing feedback on prolonged standing, which is consistent with the broader absence of standing-specific metrics in the reviewed literature. As more than two-thirds of the included studies relied on post-hoc analyses without a user-facing component, just-in-time feedback remains largely unexplored in research on prolonged posture monitoring. This gap presents an important opportunity to advance the field by integrating robust posture detection with user-centred, real-time feedback. The complete feedback characteristics for each study are provided in the data extraction table (S1 Dataset, “User feedback” sheet).

### Risk of bias assessment

The risk of bias was assessed using design-specific tools according to the type of study. Four randomised or cluster-randomised trials were evaluated with the JBI critical appraisal checklist for randomised controlled trials [27,31,35,38]. Five observational studies were assessed using the JBI critical appraisal checklist for analytical cross-sectional studies [14,32,34,36,39]. Five studies of wearable sensor validation or algorithm development were evaluated using the WEAR-BOT framework [9,13,15,33,37]. Complete operational rubrics, pre-consensus reviewer judgments, consensus item and domain-level judgments, and justification notes are available in S2 File.

The pre-consensus agreement between the authors was consistently high in all risk of bias assessments (Table 3). The overall agreement at the item/question-level reached 96.5% in 312 paired judgments, with Cohen’s κ=0.934. The tool-specific agreement was 96.2% for the JBI randomised-trial checklist (κ=0.905), 100.0% for the JBI analytical cross-sectional checklist (κ=1.000) and 95.9% for WEAR-BOT (κ=0.933) at the item-level, and 100.0% (κ=1.000) at the domain-level. Most disagreements reflected minor distinctions between “Yes” and “Probably yes”, between “No” and “Probably no”, or relating to whether treatment provider blinding should be rated as not applicable or not met in behavioural intervention studies. All disagreements were resolved through discussion.

**Table 3:** Pre-consensus inter-rater agreement for risk of bias assessments.

Assessment tool	Unit of agreement	Paired judgments	Agreements	Agreement (%)	Cohen’s κ
JBI randomized controlled trial checklist	Question-level	52	50	96.2	0.905
JBI analytical cross-sectional checklist	Question-level	40	40	100.0	1.000
WEAR-BOT	Item-level	220	211	95.9	0.933
WEAR-BOT	Domain-level	40	40	100.0	1.000
Overall	Item/question-level	312	301	96.5	0.934

**Fig 4:**
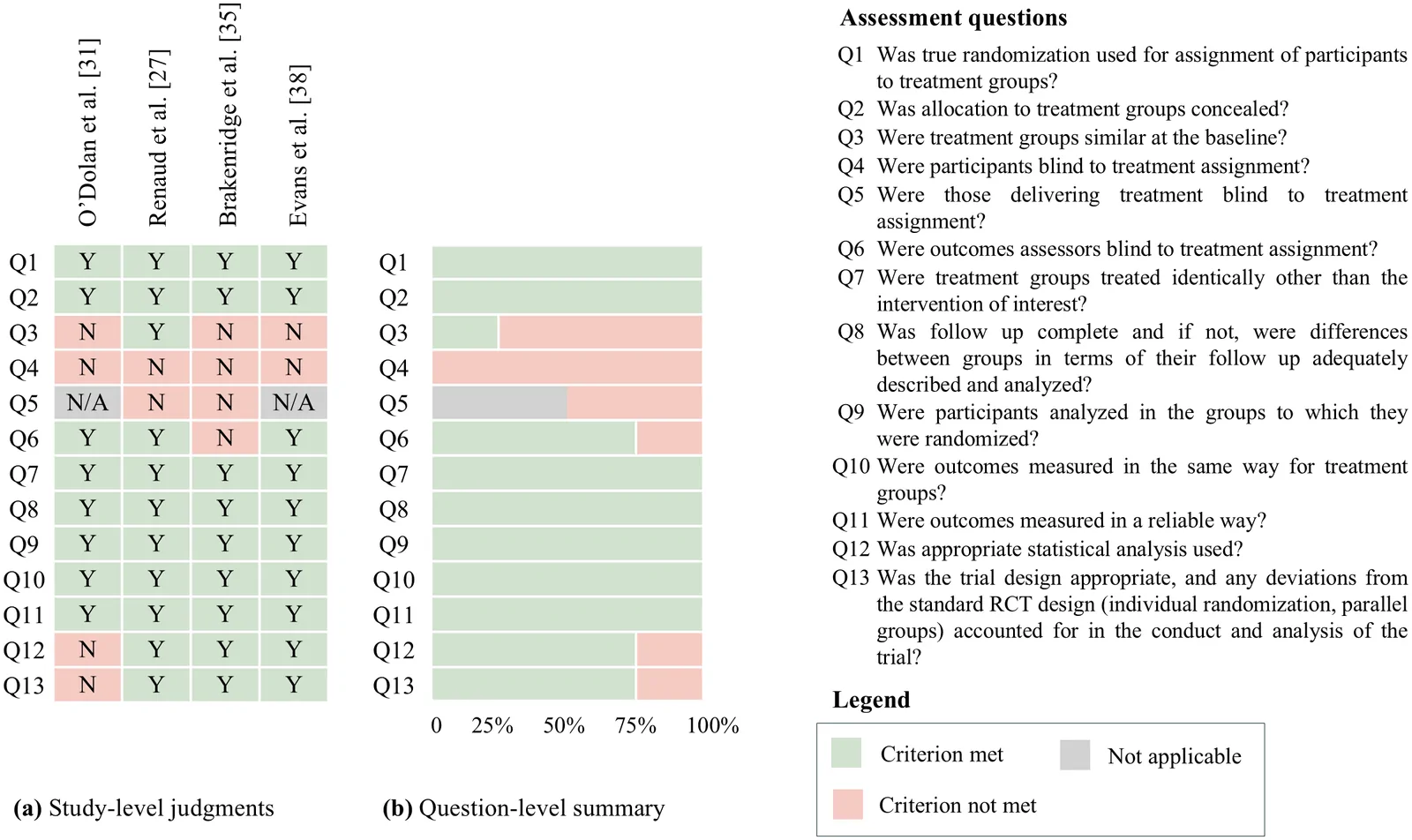
Risk of bias assessment of randomised and cluster-randomised trials using the JBI critical appraisal checklist for randomised controlled trials. **(a)** Study-level judgments for each included trial across the thirteen JBI assessment questions. **(b)** Question-level summary showing the proportion of studies rated as criterion met or criterion not met for each question.

The methodological quality of the four randomised or cluster-randomised trials was generally acceptable, with several risks of bias identified (Fig 4). All four studies satisfied criteria for true randomisation, allocation concealment, complete or adequately described follow-up, analysis according to randomised group allocation, consistent outcome measurement between groups, and reliable outcome measurement [27,31,35,38]. Three studies demonstrated appropriate statistical analysis and proper handling of the trial design [27,35,38]. Baseline similarity was adequate in only one study [27], while baseline imbalance was present in the remaining three trials [31,35,38]. As anticipated, participant blinding was not achieved in any study, given the behavioural and workplace nature of the interventions [27,31,35,38]. Nevertheless, the lack of participant blinding remained a potential source of performance bias. Blinding of the personnel who delivered the intervention was not achieved in two studies [27,35], and was considered not applicable in two studies in which no separate blinded treatment provider was involved, or blinding was not feasible [31,38]. Outcome assessor blinding was considered adequate in three studies, mainly when the prolonged posture outcomes were derived from the devices, or the assessors were reported to be blinded [27,31,38], yet one study had unclear or inadequate assessor blinding [35]. One cluster-randomised feasibility trial did not clearly account for the cluster design in its statistical analysis or in its trial design [31]. In summary, the principal concerns of the randomised and cluster-randomised trials were baseline imbalance, lack of participant and personnel blinding, and incomplete handling of cluster-specific design features.

**Fig 5:**
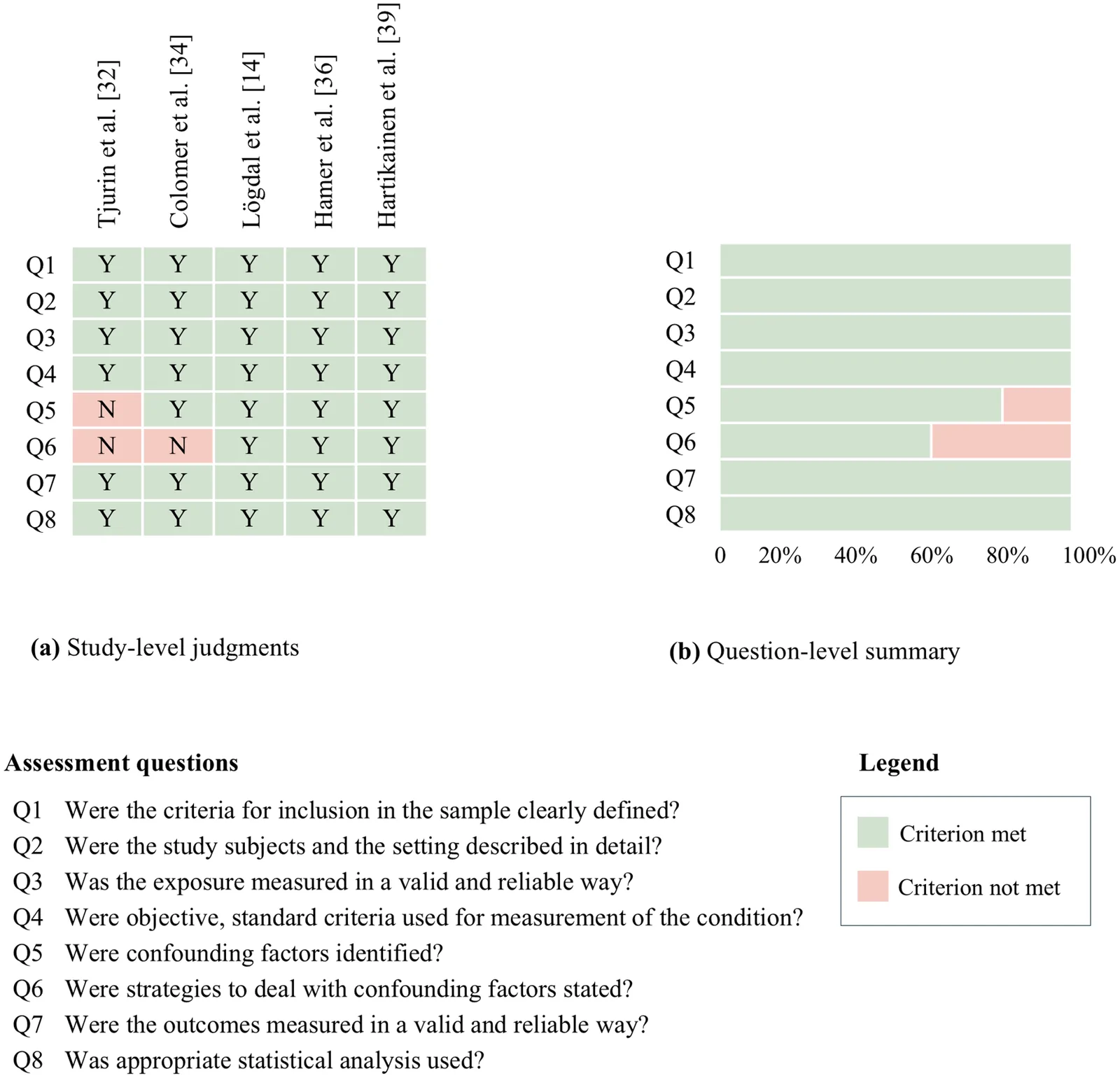
Risk of bias assessment of observational studies using the JBI critical appraisal checklist for analytical cross-sectional studies. **(a)** Study-level judgments for each included study across the eight JBI assessment questions. **(b)** Question-level summary showing the proportion of studies rated as criterion met or criterion not met for each question.

The five cross-sectional analytical studies demonstrated a generally low risk of bias (Fig 5). Each study clearly defined inclusion criteria, described study subjects and measurement settings in detail, used valid and reliable exposure measures, applied objective and standardised criteria for measuring the outcomes of prolonged posture, ensured the measurement of the results was valid and reliable, and used appropriate statistical analyses [14,32,34,36,39]. The primary limitation was related to confounding. Three studies explicitly identified relevant confounding factors and described strategies to address them [14,36,39], while two studies did not adequately identify or control confounding in their primary analyses [32,34].

**Fig 6:**
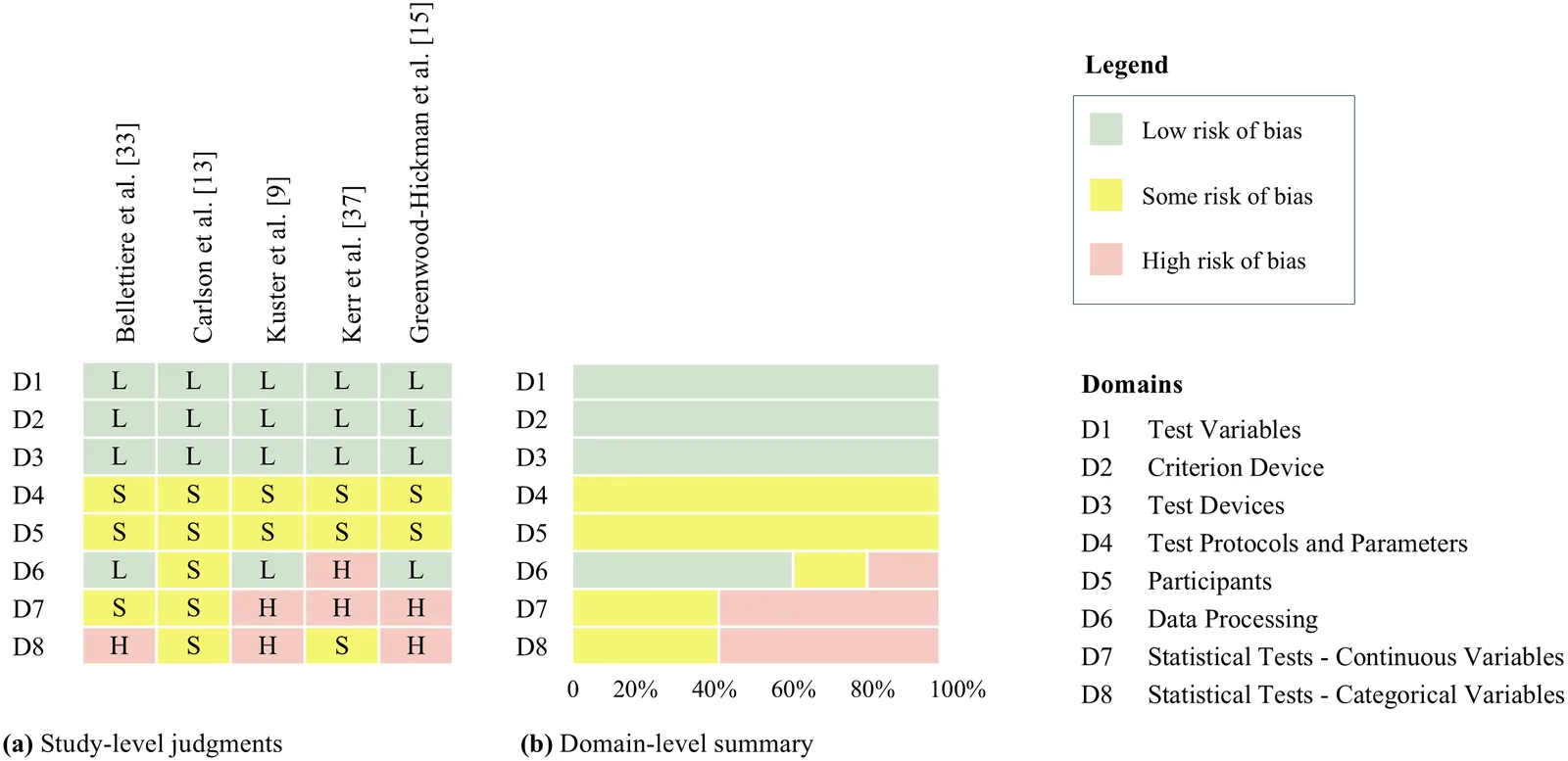
Risk of bias assessment of wearable-sensor validation and algorithm-development studies using the WEAR-BOT framework. **(a)** Study-level judgments across the eight WEAR-BOT domains. **(b)** Domain-level summary showing the proportion of studies rated as low risk of bias, some risk of bias, or high risk of bias for each domain.

Among the five wearable sensor validation or algorithm development studies assessed using WEAR-BOT, the risk of bias varied in different domains (Fig 6). All studies exhibited low risk in test variable comparability, criterion device validity, and test device reporting and use [9,13,15,33,37]. However, persistent concerns were observed in the test protocol and participant domains, primarily due to incomplete reporting, insufficient control of environmental factors, and the absence of a priori sample size justification, which are expected for free-living studies [9,13,15,33,37]. Data processing quality was higher in studies that provided detailed descriptions of preprocessing, device alignment, missing data handling, and software resources [9,13,15,33]. In contrast, one study provided less comprehensive procedures for reproducibility and missing data management [37]. The greatest concerns were observed in the statistical testing domain. Continuous variable validation was consistently rated moderate or high risk, mainly because formal equivalence tests, Bland–Altman plots or limits of agreement, and pre-specified validity thresholds were frequently omitted [9,13,15,33,37]. Categorical variable validation was also frequently limited by incomplete reporting of confusion matrices, categorical association statistics (e.g., Cohen’s kappa), or diagnostic validity thresholds [9,13,15,33,37]. In summary, the WEAR-BOT assessment indicated that the wearable validation studies were generally robust in device setup and criterion selection, but less rigorous in participant justification and in comprehensive reporting of statistical validation.

All judgments were based only on information reported in the included articles and their supplementary material. Information was not inferred from device manuals, common practice, or reviewer expertise unless explicitly stated or cited by the study.

## Discussion

This review synthesises the current state of accelerometer-based prolonged posture monitoring using a four-stage sensing-to-feedback pipeline (Fig 1). Evidence indicates that accelerometer-based monitoring is feasible for quantifying prolonged sitting. However, the field is constrained by heterogeneous definitions and analytical pipelines, limited evidence on prolonged standing, and incomplete reporting of validation. Additionally, the transition from post-hoc monitoring to real-time actionable feedback remains insufficiently explored. The following subsections interpret the findings in relation to each research sub-question and discuss implications for future research and practice.

### Principal results

This review focuses on prolonged posture monitoring, which requires posture-specific detection, uninterrupted bout segmentation, and identification of postural transitions. These requirements present distinct technical challenges compared to sedentary behaviour monitoring, which is primarily defined by low energy expenditure while sitting, reclining, or lying [4]. Although the findings support this distinction, it is not consistently operationalised in the current literature. The classification of prolonged sitting as either a subset of sedentary behaviour or as a distinct measurement target varies across studies, influencing sensor selection, bout definition, and outcome reporting [9,15,36]. This differentiation has meaningful implications for digital health, as the clinical or ergonomic interpretation of monitoring data depends not only on total sedentary or upright time but also on whether a posture is sustained continuously and when interruptions occur [8,51].

Three primary findings emerged in the sensing-to-feedback pipeline. First, the operational definition of “prolonged” varied substantially across the included studies, with bout duration thresholds ranging from 5 to 60 minutes and inconsistent reporting of interruption criteria. This definitional variability indicates that studies using the same terminology may be evaluating fundamentally different constructs, thereby limiting comparability between studies and complicating the application of evidence in practice. Second, the evidence base is notably unbalanced. In the included studies, prolonged sitting has received considerable methodological attention; whereas prolonged standing, despite its recognised associations with musculoskeletal symptoms and fatigue, was not specifically addressed by any of them. Standing was primarily considered an interruption of sitting or an undifferentiated component of “time on feet”, rather than a prolonged posture that required a dedicated bout-level analysis. Third, the technical capacity to detect prolonged sitting bouts has advanced substantially, particularly through validated ML algorithms. However, all analyses in the included studies were conducted post-hoc. Although four intervention studies incorporated feedback components, these were triggered by fixed timers or device-based reminders rather than by real-time detection of prolonged posture. The following sections interpret these findings in detail for each stage of the sensing-to-feedback pipeline and discuss their implications for future research and digital health practice.

#### Stage 1: Data collection.

The method of data collection determines the posture information available for subsequent analyses. Thigh-worn sensors, particularly activPAL, are frequently used for their high accuracy in distinguishing sitting, standing, and stepping, making this placement well-suited for criterion measurement and algorithm validation [52]. However, thigh attachment typically requires skin contact and careful attachment, which may reduce long-term adherence in certain populations [53,54]. In contrast, hip- and waist-worn accelerometers mounted on belts are widely used in large-scale activity monitoring studies, but they often suffer from non-wear time, particularly when participants remove devices during sleep or water-based activities [55].

Risk of bias assessments highlight the importance of selecting an appropriate criterion. Most validation studies used activPAL as the reference measure [9,13,15,33,37]. Although activPAL is widely validated for distinguishing sedentary and standing postures, it is not error-free. For example, non-standard sitting postures may be misclassified, and sitting and lying are not always separable with a single thigh-worn monitor [56]. In supervised learning, such label uncertainty can affect model training, as algorithms may learn systematic patterns from noisy or misaligned reference labels, particularly in time-series activity recognition tasks [57,58].

Data collection in free-living environments presents practical challenges not encountered in laboratory settings. Device displacement, inconsistent wear, misclassification of non-wear, and incomplete diary reporting can compromise data quality [53,59]. While these issues do not invalidate free-living monitoring, they highlight the need for transparent reporting of wear instructions, valid-wear criteria, attachment procedures, and data quality evaluation.

The current evidence base is limited in both population and setting coverage. Most included studies have been conducted in Western, adult, office-based populations. Without inclusive validation, digital health systems may produce outputs that appear precise but are inaccurate for intended users, particularly when algorithms are developed or validated in narrow populations and then applied across demographic, clinical, or contextual groups with different movement patterns [60–62]. Future studies should validate prolonged posture monitoring in populations where deployment is anticipated, including children, older adults, clinical groups, and occupations involving prolonged standing.

The sampling rate should be treated as a deliberate design choice rather than a technical detail. Sampling rates affect the processing and comparability of raw accelerometer data, including activity count generation and cross-device comparability [63,64]. It also influences the trade-off between signal resolution, classification performance, data volume, and battery consumption. Lower sampling rates can reduce storage and power demands, but the acceptable rate depends on monitor targets, sensor placements, and analytical models [65,66].

In summary, data collection choices in prolonged posture monitoring are design decisions that influence validity, usability, and generalisability throughout the research process. Researchers should select and report sensor configurations that align with monitoring objectives. Whether wrist-worn devices, which impose a lower user burden and are widely used in consumer wearables, can support prolonged posture monitoring remains an open question, as no included study evaluated this configuration. Practitioners should assess whether devices and placements are appropriate for the user population and the intended clinical or ergonomic application before implementing prolonged posture metrics in practice.

#### Stage 2: Data preparation.

The reviewed studies exhibited substantial variation in data preparation, from signal extraction to sleep and non-wear handling, epoching, synchronisation, and feature construction, which complicates cross-study comparisons and reproducibility [67]. For example, two studies using identical sensors and bout thresholds may result in inconsistent prolonged bout estimation if their data preparation pipelines differ.

Sleep and non-wear detection play a particularly important role. Failing to identify these time windows can lead to an overestimation of waking sedentary or sitting time, whereas overly strict non-wear algorithms may exclude valid periods of static sitting or standing [45,47,55]. Although diary-based annotation is frequently used to distinguish sleep, wake time, and monitor removal, it imposes burdens on participants and researchers and may be incomplete or inaccurate [47,53]. Algorithmic methods offer greater efficiency, but their performance can vary depending on device placement, population, wear protocol, and parameter settings [46,68]. Studies should therefore specify how sleep, non-wear, water-based activities, and partial wear were handled.

In validation studies using concurrent devices, even minor time offsets can bias transition detection and introduce bout level errors. The most rigorous studies reviewed provided detailed descriptions of alignment and time-drift correction, while less robust studies omitted these methodological details [9,37]. Future validation work should report device initialisation, synchronisation, and drift checks as standard practice.

The epoch length and segmentation rules substantially influence analytical outcomes. Different epoch lengths can change estimates of sedentary time and time accumulated in bouts, as longer epochs average behaviour over a wider time interval and may mask brief posture changes or interruptions [44,69]. In wearable-based human activity recognition, window size is also known to affect classification performance, reflecting a trade-off between temporal resolution and signal stability [70,71]. For real-time feedback, the window needs to remain sensitive to emerging prolonged bouts while resisting false alerts from brief signal fluctuations.

Feature extraction decisions affect both analytical performance and interpretability. Manually engineered features offer transparency and may generalise well in some activity recognition tasks, but require task-relevant expertise and careful feature engineering [48]. Using proprietary software to extract results is convenient, but it can limit transparency and reproducibility when the underlying algorithms are inaccessible or updated without complete methodological disclosure [72]. Deep learning models can learn features directly from raw wearable sensor signals and achieve advanced human activity recognition, but typically require large labelled datasets and careful validation [73]. The choice of feature extraction method should therefore align with the specific application. For example, transparency is crucial for clinical interpretation, while greater model complexity can be justified for predictive tasks when supported by validated data and appropriate interpretability methods [74,75].

Ultimately, preprocessing determines the interpretability of posture metrics. For example, a reported 30-minute sitting bout is meaningful only if wake time, non-wear periods, posture labels, and interruption rules are clearly defined. In digital health systems that provide feedback or workplace recommendations, this transparency is essential.

In summary, data preparation constitutes a primary source of methodological variability. Studies should document their pipelines in reproducible detail, including software, parameters, synchronisation, sleep and non-wear handling, epoching, feature extraction, and bout construction rules. For real-time applications, it is also important to distinguish preprocessing steps that are feasible in real time from those that are only possible post hoc.

#### Stage 3: Data analysis.

The reviewed studies employed analytical methods for two primary purposes: 1) applying posture metrics as epidemiological or intervention outcomes, and 2) developing algorithms for posture classification and bout estimation. This distinction is important because the required level of accuracy depends on the intended use. Measurement error may be acceptable for estimating group-level exposure if it is quantified and handled appropriately, whereas individual-level monitoring and real-time feedback require greater precision because errors can directly trigger inappropriate or missed interventions [76–78].

Another important distinction is that total sitting time, classification accuracy, transition detection, and prolonged bout estimation represent different analytical targets. A model may accurately estimate total sitting time, but may still misclassify transitions or bout durations, both of which are critical for personalised monitoring and intervention. Consequently, validation studies should report performance separately for posture classification, transition timing, total sitting time, bout duration, and prolonged bout accumulation.

Deep learning models, such as the CHAP family [13,15,33], have produced improved estimates of sitting patterns from hip-worn accelerometers compared to cut-point methods. However, these results indicate potential rather than established generalisability. Model performance in wearable-based human activity recognition is influenced by training data, population characteristics, sensor placement, preprocessing procedures, and the quality of reference labels [48,73,77]. Therefore, external validation in the intended target population is essential before deploying these models for individual-level digital health applications [60,76,77].

Reporting validation procedures requires improvement. Although several studies provided accuracy or error metrics, essential elements such as equivalence testing, Bland–Altman plots, limits of agreement, pre-specified validity thresholds, and categorical agreement statistics were often missing. Without these, it is difficult to judge whether an algorithm is suitable for specific applications.

Interpretability is also a critical consideration. Deep learning models can capture complex temporal patterns in wearable sensor data, but often lack transparency, whereas simpler models may offer greater interpretability at the expense of flexibility or performance [73,75]. When complex models are implemented, providing open-source code, reporting subgroup performance, and documenting failure cases can improve transparency, trust, and independent evaluation [74,77].

In practical applications, analytical methods should align with the specific context of decision-making. Group-level intervention evaluations may be adequately addressed by validated post-hoc bout metrics, whereas individual-level monitoring and real-time prompts require reliable transition detection with minimal latency to avoid inappropriate or missed feedback [76,78]. Clinical applications may also require integrating posture duration with symptom reports or contextual data, as digital health interventions depend not only on accurate sensing but also on timely, contextually appropriate decision rules [78,79]. Therefore, analytical performance should be assessed in relation to the intended action, rather than relying exclusively on statistical metrics.

In summary, current analytical methods have made clear progress in monitoring prolonged sitting. Future work should prioritise application-specific validation of bout-level outcomes, transition timing, subgroup robustness, and real-time feasibility.

#### Stage 4: User feedback.

Prolonged posture monitoring may serve epidemiological or evaluative purposes without providing user feedback. On the other hand, as defined by the WHO, translating the latest data, research, and evidence into action is a key objective to advance digital health and innovation [80]. Therefore, this review examines how studies translate monitoring outputs into timely and actionable support for behaviour change. This approach aligns with just-in-time adaptive intervention frameworks, which focus on providing appropriate support at the right time based on the changing state of an individual [78]. However, none of the reviewed studies progressed from validated posture classification to adaptive and personalised feedback. Instead, the four intervention studies used fixed timers or reminders instead of real-time detection. This feedback gap likely results from unresolved measurement challenges in the first three stages.

Providing effective feedback depends on reliable bout detection. For prolonged sitting, in principle, issuing a prompt when a bout reaches a predefined threshold is feasible. In contrast, for prolonged standing, where the appropriate response may involve walking or varying posture rather than simply sitting down, feedback cannot be effectively designed without standing-specific monitoring, which is largely absent. Consequently, posture-aware feedback design is limited by the capabilities of the monitoring system.

Future studies should evaluate user acceptance, the risk of notification fatigue, long-term engagement, and any unintended shifts to alternative postures. The criteria for triggering feedback need to be clearly justified, since acceptable bout lengths differ between epidemiological surveillance, workplace prevention, rehabilitation, and self-management contexts, and should be adaptable to individual needs.

### Comparison with previous work

Previous reviews have demonstrated the feasibility of wearable sensors for posture classification, physical activity monitoring, and sedentary behaviour assessment [20,21,54]. The present review builds on this foundation by focusing on prolonged posture monitoring and synthesising the entire sensing-to-feedback process. This perspective identifies three methodological issues that are often overlooked in broader reviews on wearable sensing. First, prolonged posture monitoring requires bout segmentation and transition detection, which go beyond estimating total sedentary time. Second, the operational definition of “prolonged”, including bout duration thresholds and interruption rules, varies considerably across studies. This definitional heterogeneity has not been systematically examined as a methodological issue. Third, prolonged standing is seldom addressed in previous studies. These gaps differentiate prolonged posture monitoring from general posture or activity monitoring, highlighting the need for focused methodological development.

### Limitations

Although this review adhered to the PRISMA guidelines and followed a rigorous methodology, it is subject to several limitations.

First, substantial methodological heterogeneity was observed in the 14 included studies, including differences in sensor placements, wear protocols, sampling rates, preprocessing methods, bout definitions, interruption rules, and reported results, which precluded quantitative meta-analysis. Consequently, the synthesis should be regarded as a structured methodological overview rather than as a pooled performance estimate.

Second, the evidence was uneven across both postures and populations. Although prolonged posture was defined to include both sitting and standing, the included studies predominantly examined sitting. Occupational groups with high standing exposure and clinical populations with musculoskeletal disorders were under-represented, which limits the generalisability of findings beyond office settings.

Third, the review was restricted to English-language publications and studies that employed accelerometer-based wearable sensors. Therefore, relevant studies published in other languages, in the grey literature, or that used alternative sensing modalities may have been missed.

Notably, the search strategy was intentionally designed to prioritise high sensitivity over high specificity. The large number of records identified, relative to the few studies meeting the inclusion criteria, reflects the broad application of the term “sedentary”, which covers a wider range of topics, including non-postural sedentary behaviours, screen time, and self-report research, compared to the narrower focus on prolonged posture monitoring in this review. This approach was chosen because prolonged posture monitoring is not consistently indexed as a distinct topic, and prioritising sensitivity reduces the risk of omitting relevant studies, even if it results in retrieving many irrelevant records.

### Future works

Future research should prioritise the following directions, in line with the four-stage sensing-to-feedback pipeline:

1. Prolonged standing should be treated as a distinct monitoring target. Research should include dedicated validation in standing-intensive occupations and report bout-level standing outcomes along with sitting metrics.
2. Greater consistency is needed in the operational definition of “prolonged”, including explicit justification of bout duration, interruption rules, tolerance, and wake time definitions.
3. Validation reporting should include separate performance metrics for posture classification, transition detection, total time, bout duration, and prolonged bout accumulation, in addition to overall classification accuracy. It should also incorporate equivalence testing, Bland–Altman limits of agreement, and pre-specified validity thresholds.
4. It is necessary to evaluate whether the algorithms validated in post-hoc analyses can operate with sufficient latency and reliability for real-time bout detection and closed-loop feedback.
5. The evidence base should be expanded beyond Western, office-based adult populations to include children, older adults, clinical groups, standing-intensive workers, and non-Western settings.
6. Data preparation pipelines should be reported in sufficient detail to ensure reproducibility. This includes software, parameters, synchronisation, sleep and non-wear handling, epoching, and bout-construction rules, which are essential to support cross-study comparisons and the transition to scalable digital health systems.

## Conclusion

This systematic review demonstrates the technical feasibility of accelerometer-based monitoring of prolonged sitting, particularly when validated algorithms are applied to thigh or hip-worn sensors. Nevertheless, the field remains heterogeneous, with considerable variation in the definitions of “prolonged”, inconsistent data preparation, diverse analytical methods, and limited progress in translating post-hoc classification into real-time feedback. Prolonged standing has received little attention as a distinct monitoring target, and the current evidence base is largely derived from Western, office-based, adult populations. Overcoming these limitations will require comprehensive technical reporting, inclusive validation, interpretable analytics, and sustained interdisciplinary collaboration to transition prolonged posture monitoring from a technically feasible research tool to an evidence-based scalable digital health solution.

## Supporting information

S1 Checklist Completed PRISMA 2020 checklist and abstract checklist for this systematic review. This file was adapted from the PRISMA 2020 checklist and PRISMA 2020 abstract checklist, which are distributed under the Creative Commons Attribution 4.0 International License (CC BY 4.0). The original checklists were published as part of the PRISMA 2020 statement by Page et al. [24,25].
(PDF)

S1 Protocol PROSPERO registration. Study protocol registered in PROSPERO (CRD42025637387), last updated on June 17, 2026 (Version 6.0).
(PDF)

S1 File Full search strategies for all databases. This supplement presents the full search strategies used for each database, including Web of Science, Scopus, ProQuest Central, PubMed, Embase, CINAHL, SPORTDiscus, IEEE Xplore, ACM Digital Library, and Engineering Village (EI). It provides the detailed strategies for the initial search (January 12, 2025), the updated search (December 4, 2025), and the catch-up search (April 8, 2026), the number of records retrieved from each database before deduplication, and the modifications in each search.
(PDF)

S1 Table Excluded studies. List of studies excluded during full-text screening, with primary reasons for exclusion.
(XLSX)

S1 Dataset Data extraction. Complete data extraction for all 14 included studies according to the data items listed in Table 1.
(XLSX)

S1 Fig Visual overview of study characteristics. This supplement provides a visual summary of the methodological characteristics of the 14 included studies across the sensing-to-feedback pipeline. The key aspects covered include data collection, data preparation, data analysis, user feedback, reproducibility-related reporting items, and study category.
(PDF)

S2 File Risk of bias assessment operational rubrics and per-study judgments. This supplement provides the operational rubrics applied in the risk of bias assessment, and the per-item judgments made by both reviewers (M.L. and K.H.) for each study.
(XLSX)
