# Supplementary material for: A systematic review of prolonged posture monitoring with accelerometer-based wearable sensors
Source: PLOS Digit Health. 2026 Sep 15;5(9):e0001538. doi: 10.1371/journal.pdig.0001538 (PMC13577576; doi:10.1371/journal.pdig.0001538)
Supplement: Supplementary file 2 [file pdig.0001538.s002.pdf]

Text-Only PROSPERO Registration Record (Version 6.0)

Remark: This file was prepared by the authors as a text-only version of the latest PROSPERO registration record (CRD42025637387, updated on June 17, 2026), excluding third-party branding or logos.

REVIEW TITLE AND BASIC DETAILS

Review title

A Systematic Review of Prolonged Posture Monitoring with Accelerometer-Based Wearable Sensors

Review type 1 change

None

Review objectives 1 change

Main research question: What are the current practices and techniques for prolonged posture monitoring using accelerometer-based wearable sensors?

Sub-questions:

1. Data collection: What accelerometer-based wearable sensors are used for prolonged posture monitoring and how is prolonged posture monitored in existing studies?
2. Data preparation: What techniques are applied to prepare raw accelerometer data for analysis?
3. Data analysis: What data analysis methods are used to monitor prolonged postures?
4. User feedback strategies: What feedback strategies, if any, are implemented to inform users about prolonged posture?

Keywords 1 change

Accelerometer; IMU; Wearable Sensors; Prolonged posture; Posture monitoring; Sedentary behaviour; Machine learning; Data analysis; Systematic review

SEARCHING AND SCREENING

Searches

Databases:

- General: Web of Science (SCI-EXPANDED, SSCI, AHCI, CPCI-S, CPCI-SSH, ESCI), Scopus, ProQuest Central
- Medical/biomedical: PubMed (include MEDLINE), EMBASE, CINAHL, SPORTDiscus
- Engineering: IEEE Xplore, ACM Full-text Collection, Engineering Village (EI)

Study design

Both randomized and nonrandomized study types will be included.

Included

Full-text original studies published in peer-reviewed journal articles or conference proceedings in English will be included. Other types of publications (e.g., reviews, abstracts, editorials, preprints, patents, commentaries) will be excluded.

Excluded

Other types of publications (e.g., reviews, abstracts, editorials, commentaries) will be excluded.

Link to search strategy

A full search strategy has been uploaded to PROSPERO. The PDF may be accessed through this link https://www.crd.york.ac.uk/PROSPEROFILES/9cc4e4fd377f86b5f1df7061cf57a46d.pdf.

ELIGIBILITY CRITERIA

Condition or domain being studied

Sedentary; Sedentary Lifestyle; Low back pain; Active Monitoring; Self-monitoring

The focus of this study is mainly on prolonged posture monitoring using accelerometer-based wearable sensors.

Population

Any individuals engaging in prolonged postures of any age and population group are of interest.

Intervention(s) or exposure(s)

Accelerometer-based wearable sensing technologies.

Comparator(s) or control(s)

This review does not have any comparators

Context

Inclusion criteria

Studies were included if they met all of the following conditions:

Monitor prolonged sitting and/or standing bouts

The study focused on or reported the monitoring of prolonged sitting and/or standing bouts during wake time. For the purposes of this review, prolonged posture is defined as uninterrupted sitting and/or standing bouts lasting at least 10 minutes.

Use of accelerometer-based wearable sensors

Eligible studies must use at least one body-worn accelerometer-based sensor as the primary data collection tool. Smartphone-based studies are eligible if the phone is firmly worn on the body and its built-in accelerometer is used for posture detection.

Sufficient methodological reporting

The study provided adequate detail to allow interpretation of the monitoring of prolonged posture. At minimum, this included: (i) population; (ii) sensor type and anatomical placement; (iii) measurement protocol, such as wear duration and tasks; and (iv) methods used to derive prolonged posture outcomes.

Population and publication

- Population: Conducted with human participants.
- Language: Full-text articles published in English.
- Publication type: Original peer-reviewed journal articles or peer-reviewed full-paper conference proceedings (published, early access, and in press).

Exclusion criteria

Studies that did not meet all the inclusion criteria were excluded.

OUTCOMES TO BE ANALYSED

Main outcomes 1 change

Measures of effect

1. Sensing technologies: This outcome will explore the aims, types, placements, and the outcomes of accelerometer-based wearable sensing technologies used for prolonged posture monitoring.
2. Data preparation and analysis methods: This outcome focuses on the data preparation and analysis techniques used in the studies.

Additional outcomes

None.

Measures of effect

None.

DATA COLLECTION PROCESS

Data extraction (selection and coding)

The review will be conducted following the PRISMA guidelines. The same search terms will be used for all the selected databases. After removing the duplicates, two reviewers will blindly screen the records following the inclusion/exclusion criteria. Disagreements will be resolved

through the 'tiebreaker'. If necessary, consult the other authors. Records retrieved from the database searches will be imported into EndNote web to de-duplicate, then be imported to Rayyan for screening. Two reviewers will independently screen titles and abstracts against the predefined eligibility criteria.

Studies will be included if they focus on the monitoring of prolonged sitting and/or standing bouts during wake time, use at least one body-worn accelerometer-based sensor as the primary measurement tool, and provide sufficient methodological detail to allow interpretation of how prolonged posture was monitored. For this review, prolonged posture is defined as uninterrupted sitting and/or standing bouts lasting at least 10 minutes. Studies will be excluded if they do not address prolonged sitting and/or standing during wake time, focus only on bouts shorter than 10 minutes, report generic sedentary behavior or physical activity without a methodological contribution to prolonged posture monitoring, rely only on intensity-threshold approaches without posture-specific detection, or focus on sleep.

An online database will be created to save the documents. That database will have a daily backup and is shared within the authors. A standardized data extraction form will be used to collect study characteristics, participant and setting characteristics, study design, sensor and device characteristics, device placement, data collection protocols, definitions of prolonged sitting and/or standing, reference or annotation methods, data processing and analytical approaches, outcomes related to prolonged posture monitoring, key findings, and main conclusions.

Risk of bias (quality) assessment

Methodological quality and risk of bias will be assessed using design-appropriate tools, reflecting the heterogeneous study designs included in this review. Method development and validation studies will be appraised using the WEArable Technology Risk of Bias and Objectivity Tool (WEAR-BOT) validity checklist. Observational studies will be appraised using the Joanna Briggs Institute (JBI) critical appraisal checklist for analytical cross-sectional studies. Intervention studies will be appraised using the JBI critical appraisal checklist for randomized controlled trials. Two reviewers will assess studies independently, inter-rater agreement will be quantified before consensus, and disagreements will be resolved by discussion or third-reviewer adjudication. Per-study domain ratings will be reported; no weighted overall score will be used.

In addition to the formal tools, appraisal will focus on characteristics particularly relevant to prolonged posture monitoring with accelerometers, including device type and placement, criterion/reference method, synchronization and alignment procedures, data collection setting, preprocessing transparency, handling of missing or cleaned data, outcome definition, and the appropriateness of validity metrics and statistical analyses. Because the aim of this review is to synthesize monitoring methods rather than estimate intervention effects, design-appropriate methodological appraisal tools will be used rather than a single universal risk-of-bias instrument.

PLANNED DATA SYNTHESIS

Strategy for data synthesis

The data synthesis strategy will be carried out following the guidance of Campbell et al. (2019).

Analysis of subgroups or subsets

No.

REVIEW AFFILIATION, FUNDING AND PEER REVIEW

Review team members

Ms Meixing Liao. KU Leuven. Belgium.

No conflict of interest declared.

Mr Kai Huang. School of Economics and Management, Beihang University, China; M3-BIORES (Measure, Model & Manage Bioresponses), Department of Biosystems, KU Leuven. Belgium.

No conflict of interest declared.

Ms Eva Libeert (review guarantor). M3-BIORES (Measure, Model & Manage Bioresponses), Department of Biosystems, KU Leuven. Belgium.

No conflict of interest declared.

Named contact

Ms Meixing Liao (meixing.liao@kuleuven.be). KU Leuven. Belgium.

Review affiliation

KU Leuven, Beihang University

Funding source

China Scholarship Council.

Grant number

No.202208090040; No.202406020168

Additional non-commercial funding information

The Academic Excellence Foundation of BUAA for PhD Students

TIMELINE OF THE REVIEW

Review timeline

Start date: 01 November 2024. End date: 28 February 2025

Date of first submission to PROSPERO

25 January 2025

Date of registration in PROSPERO

05 February 2025

CURRENT REVIEW STAGE

Publication of review results

Results of the review will be published in English.

Preprint publication

Meixing Liao, Kai Huang, Eva Libeert, Bart Vanrumste, Simon Brumagne, Zhe Chen, Jean-Marie Aerts. A Systematic Review of Prolonged Posture Monitoring With Accelerometer-Based Wearable Sensors (Preprint) . doi:10.2196/preprints.79437

Journal publication

Not yet published in a journal but will be in future.

Stage of the review at this submission 1 change

**Review stage**	**Started**	**Completed**
Pilot work	✓	✓
Formal searching/study identification	✓	✓
Screening search results against inclusion criteria	✓	✓
Data extraction or receipt of IPD	✓	✓
Risk of bias/quality assessment	✓	✓
Data synthesis	✓	✓

Review status

The review is completed.

ADDITIONAL INFORMATION

Additional information

Collaborators

Not applicable.

- **Professor Jean-Marie Aerts**, M3-BIORES (Measure, Model & Manage Bioresponses), Department of Biosystems, KU Leuven
- **Assistant/Associate Professor Zhe Chen**, School of Economics and Management, Beihang University
- **Professor Simon Brumagne**, Research Group for Musculoskeletal Rehabilitation, Department of Rehabilitation Sciences, KU Leuven

- **Professor Bart Vanrumste**, e-Media Research Lab, Department of Electrical Engineering (ESAT), KU Leuven

PROSPERO version history 1 change

- Version 6.0, published 17 Jun 2026
- Version 5.0, published 18 Apr 2026
- Version 4.0, published 12 Apr 2026
- Version 3.0, published 24 Dec 2025
- Version 2.2, published 24 Dec 2025
- Version 2.1, published 30 Jul 2025
- Version 2.0, published 06 May 2025
- Version 1.1, published 05 Feb 2025
- Version 1.0, published 05 Feb 2025

Review conflict of interest

None known

Country

Belgium; China

Other registration details

Not applicable.

Medical Subject Headings

Accelerometry; Data Collection; Feedback; Humans; Low Back Pain; Musculoskeletal Diseases; Posture; Wearable Electronic Devices

Details of any existing review of the same topic by the same authors

Not applicable.

Revision note 1 change

Updated sub-research questions and keywords to keep consistent with the publication.

Disclaimer

The content of this record displays the information provided by the review team. PROSPERO does not peer review registration records or endorse their content.

PROSPERO accepts and posts the information provided in good faith; responsibility for record content rests with the review team. The guarantor for this record has affirmed that the information provided is truthful and that they understand that deliberate provision of inaccurate information may be construed as scientific misconduct.

PROSPERO does not accept any liability for the content provided in this record or for its use. Readers use the information provided in this record at their own risk.

Any enquiries about the record should be referred to the named review contact
