# Supplementary material for: A systematic review of prolonged posture monitoring with accelerometer-based wearable sensors
Source: PLOS Digit Health. 2026 Sep 15;5(9):e0001538. doi: 10.1371/journal.pdig.0001538 (PMC13577576; doi:10.1371/journal.pdig.0001538)
Supplement: Supplementary file 3 [file pdig.0001538.s003.pdf]

Search strategy

Concept 1: Wearable accelerometer-based sensors

Relevant MeSH terms: "Wearable Electronic Devices"[MeSH] OR "Accelerometry"[MeSH]

Key words (initial search, Jan 12, 2025): "inertial" OR "inertia" OR "IMU" OR "IMUs" OR "wearable*" OR "actigraph*" OR "accelerome*" OR "acceleration" OR "magnetometer" OR "magnetometers" OR "gyroscope" OR "gyroscopes" OR "activity tracker" OR "activity trackers" OR "fitness tracker" OR "fitness trackers" OR "fitness band" OR "fitness bands" OR "body-worn sensor" OR "body-worn sensors" OR "body worn sensor" OR "body worn sensors" OR "motion sensor" OR "motion sensors" OR "motion tracker" OR "motion trackers" OR "motion capture" OR "smartwatch" OR "smart watch"

Key words (update search, Dec 4, 2025; catch-up search, Apr 8, 2026): "accelerome*" OR "acceleration" OR "actigraph*" OR "activPAL" OR "Axivity" OR "GENEActiv" OR "Xsens" OR "Shimmer" OR "Fitbit" OR "Garmin" OR "Polar" OR "Apple Watch" OR "wearable*" OR "smartwatch" OR "smart watch" OR "activity tracker" OR "activity trackers" OR "fitness tracker" OR "fitness trackers" OR "fitness band" OR "fitness bands" OR "inertial" OR "inertia" OR "IMU" OR "IMUs" OR "IMU-based" OR "Movesense" OR "movisens" OR "body-worn sensor" OR "body-worn sensors" OR "body worn sensor" OR "body worn sensors" OR "motion sensor" OR "motion sensors" OR "motion tracker" OR "motion trackers" OR "motion capture"

Modifications:

- Removed: "magnetometer" OR "magnetometers" OR "gyroscope" OR "gyroscopes"
- Added: "activPAL" OR "Axivity" OR "GENEActiv" OR "Xsens" OR "Shimmer" OR "Fitbit" OR "Garmin" OR "Polar" OR "Apple Watch" OR "Movesense" OR "movisens" OR "IMU-based"
- Rationale: The review focuses on accelerometer-based methods; therefore, we removed terms related to other sensor types. In the meantime, we added the widely used sensor brands in the field.

Concept 2: Prolonged posture

Relevant MeSH term: "Sedentary Behavior"[Mesh]

Key words (initial search, Jan 12, 2025): "prolonged posture" OR "prolonged postures" OR "sedentary" OR "prolonged sitting" OR "prolonged standing" OR "sitting time" OR "sitting posture" OR "sitting postures" OR "standing time" OR "standing posture" OR "standing postures" OR "static posture" OR "static postures" OR "static sitting" OR "static standing"

Key words (update search, Dec 4, 2025; catch-up search, Apr 8, 2026): "prolonged posture" OR "prolonged postures" OR "sedentary" OR "prolonged sitting" OR "prolonged standing" OR "static posture" OR "static postures" OR "static sitting" OR "static standing" OR "sitting time" OR "standing time" OR "sedentary bout" OR "sedentary bouts" OR "sitting bout" OR "sitting bouts" OR "standing bout" OR "standing bouts" OR "posture bout" OR "posture bouts" OR "uninterrupted sitting" OR "uninterrupted standing" OR "sustained sitting" OR "sustained standing" OR "sustained posture" OR "bout duration" OR "bout durations" OR "bout length" OR "posture duration" OR "posture durations" OR "sit-to-stand" OR "sit to stand" OR "stand-to-sit" OR "stand to sit" OR "posture transition" OR "posture change" OR "posture monitoring" OR

"posture detection" OR "posture classification" OR "posture recognition" OR "sitting posture" OR "sitting postures" OR "standing posture" OR "standing postures"

Modifications:

- Added "sedentary bout" OR "sedentary bouts" OR "sitting bout" OR "sitting bouts" OR "standing bout" OR "standing bouts" OR "posture bout" OR "posture bouts" OR "uninterrupted sitting" OR "uninterrupted standing" OR "sustained sitting" OR "sustained standing" OR "sustained posture" OR "bout duration" OR "bout durations" OR "bout length" OR "posture duration" OR "posture durations" OR "sit-to-stand" OR "sit to stand" OR "stand-to-sit" OR "stand to sit" OR "posture transition" OR "posture change" OR "posture monitoring" OR "posture detection" OR "posture classification" OR "posture recognition"
- Rationale: We incorporated bout-, duration-, and transition-related terms to more accurately represent the review's emphasis on uninterrupted sitting or standing, temporal continuity, and the segmentation of prolonged posture bouts. These additions aimed to enhance the retrieval of studies that define prolonged posture based on bout duration and posture transitions, rather than relying exclusively on general sedentary behavior terminology.

Concept 3: Data analysis methods

Relevant MeSH terms: "Electronic Data Processing"[Mesh] OR "Data Analysis"[Mesh] OR "Algorithms"[Mesh]

Key words (initial search, Jan 12, 2025): "machine learning" OR "deep learning" OR "neural network" OR "neural networks" OR "NN" OR "NNs" OR "CNN" OR "CNNs" OR "RNN" OR "RNNs" OR "DNN" OR "DNNs" OR "KNN" OR "k-nearest neighbors" OR "SVM" OR "support vector machine" OR "LSTM" OR "XGBoost" OR "catboost" OR "adaboost" OR "decision tree" OR "decision trees" OR "random forest" OR "random forests" OR "supervised" OR "unsupervised" OR "Naive Bayes" OR " Principal Component Analysis" OR "PCA" OR "dimension reduction" OR "linear discriminate" OR "LDA" OR "transformer" OR "reinforcement learning" OR "computer vision" OR "artificial intelligence" OR "AI" OR "unsupervised learning" OR "supervised learning" OR "data analysis" OR "data analyses" OR "feature extraction" OR "data preprocessing" OR "data processing" OR "signal processing" OR "information processing" OR "feature engineering" OR "statistical model" OR "ANOVA" OR "algorithm" OR "algorithms" OR "classification" OR "classifier" OR "time-series analysis" OR "time-series analyses" OR "real-time analysis" OR "real-time analyses" OR "sentiment analysis" OR "sentiment analyses" OR "pattern analysis" OR "pattern analyses" OR "prediction" OR "predictor" OR "predictors" OR "regression" OR "regressor" OR "regressors"

Key words (update search, Dec 4, 2025; catch-up search, Apr 8, 2026): "artificial intelligence" OR "AI" OR "machine learning" OR "deep learning" OR "supervised learning" OR "unsupervised learning" OR "reinforcement learning" OR "transfer learning" OR "neural network" OR "neural networks" OR "NN" OR "NNs" OR "CNN" OR "CNNs" OR "RNN" OR "RNNs" OR "LSTM" OR "DNN" OR "DNNs" OR "transformer" OR "support vector machine" OR "SVM" OR "decision tree" OR "decision trees" OR "random forest" OR "random forests" OR "XGBoost" OR "CatBoost" OR "AdaBoost" OR "KNN" OR "k-nearest neighbors" OR "Naive Bayes" OR "feature extraction" OR "feature engineering" OR "dimension reduction" OR "Principal Component Analysis" OR "PCA" OR "linear discriminant" OR "LDA" OR "data preprocessing" OR "data processing" OR "signal

processing" OR "data analysis" OR "data analyses" OR "classification" OR "classifier" OR "regression" OR "regressor" OR "regressors" OR "prediction" OR "predictor" OR "predictors" OR "pattern analysis" OR "pattern analyses" OR "time-series analysis" OR "time-series analyses" OR "statistical model" OR "statistical learning" OR "threshold" OR "threshold-based" OR "rule-based" OR "real-time analysis" OR "real-time analyses" OR "algorithm" OR "algorithms"

Modifications:

- Removed: "supervised" OR "unsupervised" OR "computer vision" OR "ANOVA" OR "sentiment analysis" OR "sentiment analyses"
- Added: "transfer learning" OR "statistical learning" OR "threshold" OR "threshold-based" OR "rule-based"
- Rationale: Terms with broad or low specificity, such as "supervised", "unsupervised", "computer vision", "ANOVA", "sentiment analysis", and "sentiment analyses" were removed, while terms such as threshold, threshold-based, rule-based, statistical learning, and transfer learning were incorporated to more accurately represent the analytical approaches employed in prolonged posture monitoring.

Table I lists the identified results in each database in each search before deduplication.

Table I Identified results in each database before deduplication.

**Database**	**Initial Search (January 12, 2025)**	**Updated search (December 4, 2025)**	**Catch-up search (April 8, 2026)**
Web of Science	3191	4299	4604
Scopus	2752	4073	4561
ProQuest Central	985	1309	1414
PubMed	2628	3270	3407
Embase	4268	8326	8658
CINAHL	989	1182	1257
SPORTDiscus	692	763	815
IEEE Xplore	679	805	1187
ACM Digital Library	23	38	59
Engineering Village (EI)	438	Didn't extract[1]	1372
**Total**	16645	24065	27334

[1]Engineering Village (EI) was not re-searched during the update on December 4, 2025, because results from the initial January 2025 search were already included by other databases. To ensure completeness and transparency, EI was subsequently re-searched during the catch-up search on April 8, 2026, which identified 1,372 records.

The subsequent pages present database-specific search queries, organized by individual database and search. Each concept's search query is highlighted using distinct colors: concept 1 in grey, concept 2 in green, and concept 3 in purple. Search-specific conditions and remarks are documented in their respective sections.

Web of science

Search in: Web of Science Core Collection

Editions: Science Citation Index Expanded (SCI-EXPANDED)--1900-present, Social Sciences Citation Index (SSCI)--1900-present, Arts & Humanities Citation Index (A&HCI)--1975-present, Conference Proceedings Citation Index – Science (CPCI-S)--1900-present, Conference Proceedings Citation Index – Social Science & Humanities (CPCI-SSH)--1900-present, Emerging Sources Citation Index (ESCI)--2005-present

No other filters applied during database searching.

Initial search on Jan 12, 2025

Queries:

#1 TS=("inertial" OR "inertia" OR "IMU" OR "IMUs" OR "wearable*" OR "actigraph*" OR "accelerome*" OR "acceleration" OR "magnetometer" OR "magnetometers" OR "gyroscope" OR "gyroscopes" OR "activity tracker" OR "activity trackers" OR "fitness tracker" OR "fitness trackers" OR "fitness band" OR "fitness bands" OR "body-worn sensor" OR "body-worn sensors" OR "body worn sensor" OR "body worn sensors" OR "motion sensor" OR "motion sensors" OR "motion tracker" OR "motion trackers" OR "motion capture" OR "smartwatch" OR "smart watch")

#2 TS=("prolonged posture" OR "prolonged postures" OR "sedentary" OR "prolonged sitting" OR "prolonged standing" OR "sitting time" OR "sitting posture" OR "sitting postures" OR "standing time" OR "standing posture" OR "standing postures" OR "static posture" OR "static postures" OR "static sitting" OR "static standing")

#3 TS=("machine learning" OR "deep learning" OR "neural network" OR "neural networks" OR "NN" OR "NNs" OR "CNN" OR "CNNs" OR "RNN" OR "RNNs" OR "DNN" OR "DNNs" OR "KNN" OR "k-nearest neighbors" OR "SVM" OR "support vector machine" OR "LSTM" OR "XGBoost" OR "catboost" OR "adaboost" OR "decision tree" OR "decision trees" OR "random forest" OR "random forests" OR "supervised" OR "unsupervised" OR "Naive Bayes" OR " Principal Component Analysis" OR "PCA" OR "dimension reduction" OR "linear discriminate" OR "LDA" OR "transformer" OR "reinforcement learning" OR "computer vision" OR "artificial intelligence" OR "AI" OR "unsupervised learning" OR "supervised learning" OR "data analysis" OR "data analyses" OR "feature extraction" OR "data preprocessing" OR "data processing" OR "signal processing" OR "information processing" OR "feature engineering" OR "statistical model" OR "ANOVA" OR "algorithm" OR "algorithms" OR "classification" OR "classifier" OR "time-series analysis" OR "time-series analyses" OR "real-time analysis" OR "real-time analyses" OR "sentiment analysis" OR "sentiment analyses" OR "pattern analysis" OR "pattern analyses" OR "prediction" OR "predictor" OR "predictors" OR "regression" OR "regressor" OR "regressors")

#4 #1 AND #2 AND #3

Number of records identified: 3191.

Updated search on Dec 4, 2025

Queries:

#5 TS=("accelerome*" OR "acceleration" OR "actigraph*" OR "activPAL" OR "Axivity" OR "GENEActiv" OR "Xsens" OR "Shimmer" OR "Fitbit" OR "Garmin" OR "Polar" OR "Apple Watch" OR "wearable*" OR "smartwatch" OR "smart watch" OR "activity tracker" OR "activity trackers" OR "fitness tracker" OR "fitness trackers" OR "fitness band" OR "fitness bands" OR "inertial" OR "inertia" OR "IMU" OR "IMUs" OR "IMU-based" OR "Movesense" OR "movisens" OR "body-worn sensor" OR "body-worn sensors" OR "body worn sensor" OR "body worn sensors" OR "motion sensor" OR "motion sensors" OR "motion tracker" OR "motion trackers" OR "motion capture")

#6 TS=("prolonged posture" OR "prolonged postures" OR "sedentary" OR "prolonged sitting" OR "prolonged standing" OR "static posture" OR "static postures" OR "static sitting" OR "static standing" OR "sitting time" OR "standing time" OR "sedentary bout" OR "sedentary bouts" OR "sitting bout" OR "sitting bouts" OR "standing bout" OR "standing bouts" OR "posture bout" OR "posture bouts" OR "uninterrupted sitting" OR "uninterrupted standing" OR "sustained sitting" OR "sustained standing" OR "sustained posture" OR "bout duration" OR "bout durations" OR "bout length" OR "posture duration" OR "posture durations" OR "sit-to-stand" OR "sit to stand" OR "stand-to-sit" OR "stand to sit" OR "posture transition" OR "posture change" OR "posture monitoring" OR "posture detection" OR "posture classification" OR "posture recognition" OR "sitting posture" OR "sitting postures" OR "standing posture" OR "standing postures")

#7 TS=("artificial intelligence" OR "AI" OR "machine learning" OR "deep learning" OR "supervised learning" OR "unsupervised learning" OR "reinforcement learning" OR "transfer learning" OR "neural network" OR "neural networks" OR "NN" OR "NNs" OR "CNN" OR "CNNs" OR "RNN" OR "RNNs" OR "LSTM" OR "DNN" OR "DNNs" OR "transformer" OR "support vector machine" OR "SVM" OR "decision tree" OR "decision trees" OR "random forest" OR "random forests" OR "XGBoost" OR "CatBoost" OR "AdaBoost" OR "KNN" OR "k-nearest neighbors" OR "Naive Bayes" OR "feature extraction" OR "feature engineering" OR "dimension reduction" OR "Principal Component Analysis" OR "PCA" OR "linear discriminant" OR "LDA" OR "data preprocessing" OR "data processing" OR "signal processing" OR "data analysis" OR "data analyses" OR "classification" OR "classifier" OR "regression" OR "regressor" OR "regressors" OR "prediction" OR "predictor" OR "predictors" OR "pattern analysis" OR "pattern analyses" OR "time-series analysis" OR "time-series analyses" OR "statistical model" OR "statistical learning" OR "threshold" OR "threshold-based" OR "rule-based" OR "real-time analysis" OR "real-time analyses" OR "algorithm" OR "algorithms")

#8 #5 AND #6 AND #7

Number of records identified: 4299.

Catch-up search on Apr 8, 2026

Queries: same as December 4, 2025.

Number of records identified: 4604.

Scopus

No additional filter applied during database searching.

Initial search on Jan 12, 2025

Queries:

TITLE-ABS (

("inertial" OR "inertia" OR "IMU" OR "IMUs" OR "wearable*" OR "actigraph*" OR "accelerome*" OR "acceleration" OR "magnetometer" OR "magnetometers" OR "gyroscope" OR "gyroscopes" OR "activity tracker" OR "activity trackers" OR "fitness tracker" OR "fitness trackers" OR "fitness band" OR "fitness bands" OR "body-worn sensor" OR "body-worn sensors" OR "body worn sensor" OR "body worn sensors" OR "motion sensor" OR "motion sensors" OR "motion tracker" OR "motion trackers" OR "motion capture" OR "smartwatch" OR "smart watch")

AND ("prolonged posture" OR "prolonged postures" OR "sedentary" OR "prolonged sitting" OR "prolonged standing" OR "sitting time" OR "sitting posture" OR "sitting postures" OR "standing time" OR "standing posture" OR "standing postures" OR "static posture" OR "static postures" OR "static sitting" OR "static standing")

AND ("machine learning" OR "deep learning" OR "neural network" OR "neural networks" OR "NN" OR "NNs" OR "CNN" OR "CNNs" OR "RNN" OR "RNNs" OR "DNN" OR "DNNs" OR "KNN" OR "k-nearest neighbors" OR "SVM" OR "support vector machine" OR "LSTM" OR "XGBoost" OR "catboost" OR "adaboost" OR "decision tree" OR "decision trees" OR "random forest" OR "random forests" OR "supervised" OR "unsupervised" OR "Naive Bayes" OR " Principal Component Analysis" OR "PCA" OR "dimension reduction" OR "linear discriminate" OR "LDA" OR "transformer" OR "reinforcement learning" OR "computer vision" OR "artificial intelligence" OR "AI" OR "unsupervised learning" OR "supervised learning" OR "data analysis" OR "data analyses" OR "feature extraction" OR "data preprocessing" OR "data processing" OR "signal processing" OR "information processing" OR "feature engineering" OR "statistical model" OR "ANOVA" OR "algorithm" OR "algorithms" OR "classification" OR "classifier" OR "time-series analysis" OR "time-series analyses" OR "real-time analysis" OR "real-time analyses" OR "sentiment analysis" OR "sentiment analyses" OR "pattern analysis" OR "pattern analyses" OR "prediction" OR "predictor" OR "predictors" OR "regression" OR "regressor" OR "regressors"))

OR AUTHKEY (

("inertial" OR "inertia" OR "IMU" OR "IMUs" OR "wearable*" OR "actigraph*" OR "accelerome*" OR "acceleration" OR "magnetometer" OR "magnetometers" OR "gyroscope" OR "gyroscopes" OR "activity tracker" OR "activity trackers" OR "fitness tracker" OR "fitness trackers" OR "fitness band" OR "fitness bands" OR "body-worn sensor" OR "body-worn sensors" OR "body worn sensor" OR "body worn sensors" OR "motion sensor" OR "motion sensors" OR "motion tracker" OR "motion trackers" OR "motion capture" OR "smartwatch" OR "smart watch")

AND ("prolonged posture" OR "prolonged postures" OR "sedentary" OR "prolonged sitting" OR "prolonged standing" OR "sitting time" OR "sitting posture" OR "sitting postures" OR "standing time" OR "standing posture" OR "standing postures" OR "static posture" OR "static postures" OR "static sitting" OR "static standing")

AND ("machine learning" OR "deep learning" OR "neural network" OR "neural networks" OR "NN" OR "NNs" OR "CNN" OR "CNNs" OR "RNN" OR "RNNs" OR "DNN" OR "DNNs" OR "KNN" OR "k-nearest neighbors" OR "SVM" OR "support vector machine" OR "LSTM" OR "XGBoost" OR "catboost" OR "adaboost" OR "decision tree" OR "decision trees" OR "random forest" OR "random forests" OR "supervised" OR "unsupervised" OR "Naive Bayes" OR " Principal Component Analysis" OR "PCA" OR "dimension reduction" OR "linear discriminate" OR "LDA" OR "transformer" OR "reinforcement learning" OR "computer vision" OR "artificial intelligence" OR "AI" OR "unsupervised learning" OR "supervised learning" OR "data analysis" OR "data analyses" OR "feature extraction" OR "data preprocessing" OR "data processing" OR "signal processing" OR "information processing" OR "feature engineering" OR "statistical model" OR "ANOVA" OR "algorithm" OR "algorithms" OR "classification" OR "classifier" OR "time-series analysis" OR "time-series analyses" OR "real-time analysis" OR "real-time analyses" OR "sentiment analysis" OR "sentiment analyses" OR "pattern analysis" OR "pattern analyses" OR "prediction" OR "predictor" OR "predictors" OR "regression" OR "regressor" OR "regressors"))

Number of records identified: 2752.

Updated search on Dec 4, 2025

Queries:

TITLE-ABS (

("accelerome*" OR "acceleration" OR "actigraph*" OR "activPAL" OR "Axivity" OR "GENEActiv" OR "Xsens" OR "Shimmer" OR "Fitbit" OR "Garmin" OR "Polar" OR "Apple Watch" OR "wearable*" OR "smartwatch" OR "smart watch" OR "activity tracker" OR "activity trackers" OR "fitness tracker" OR "fitness trackers" OR "fitness band" OR "fitness bands" OR "inertial" OR "inertia" OR "IMU" OR "IMUs" OR "IMU-based" OR "Movesense" OR "movisens" OR "body-worn sensor" OR "body-worn sensors" OR "body worn sensor" OR "body worn sensors" OR "motion sensor" OR "motion sensors" OR "motion tracker" OR "motion trackers" OR "motion capture")

AND ("prolonged posture" OR "prolonged postures" OR "sedentary" OR "prolonged sitting" OR "prolonged standing" OR "static posture" OR "static postures" OR "static sitting" OR "static standing" OR "sitting time" OR "standing time" OR "sedentary bout" OR "sedentary bouts" OR "sitting bout" OR "sitting bouts" OR "standing bout" OR "standing bouts" OR "posture bout" OR "posture bouts" OR "uninterrupted sitting" OR "uninterrupted standing" OR "sustained sitting" OR "sustained standing" OR "sustained posture" OR "bout duration" OR "bout durations" OR "bout length" OR "posture duration" OR "posture durations" OR "sit-to-stand" OR "sit to stand" OR "stand-to-sit" OR "stand to sit" OR "posture transition" OR "posture change" OR "posture monitoring" OR "posture detection" OR "posture classification" OR "posture recognition" OR "sitting posture" OR "sitting postures" OR "standing posture" OR "standing postures")

AND ("artificial intelligence" OR "AI" OR "machine learning" OR "deep learning" OR "supervised learning" OR "unsupervised learning" OR "reinforcement learning" OR "transfer learning" OR "neural network" OR "neural networks" OR "NN" OR "NNs" OR "CNN" OR "CNNs" OR "RNN" OR "RNNs" OR "LSTM" OR "DNN" OR "DNNs" OR "transformer" OR "support vector machine" OR "SVM" OR "decision tree" OR "decision trees" OR "random forest" OR "random forests" OR "XGBoost" OR "CatBoost" OR "AdaBoost" OR "KNN" OR "k-nearest neighbors" OR "Naive Bayes" OR "feature extraction" OR "feature engineering" OR "dimension reduction" OR

"Principal Component Analysis" OR "PCA" OR "linear discriminant" OR "LDA" OR "data preprocessing" OR "data processing" OR "signal processing" OR "data analysis" OR "data analyses" OR "classification" OR "classifier" OR "regression" OR "regressor" OR "regressors" OR "prediction" OR "predictor" OR "predictors" OR "pattern analysis" OR "pattern analyses" OR "time-series analysis" OR "time-series analyses" OR "statistical model" OR "statistical learning" OR "threshold" OR "threshold-based" OR "rule-based" OR "real-time analysis" OR "real-time analyses" OR "algorithm" OR "algorithms"))

OR AUTHKEY (

("accelerome*" OR "acceleration" OR "actigraph*" OR "activPAL" OR "Axivity" OR "GENEActiv" OR "Xsens" OR "Shimmer" OR "Fitbit" OR "Garmin" OR "Polar" OR "Apple Watch" OR "wearable*" OR "smartwatch" OR "smart watch" OR "activity tracker" OR "activity trackers" OR "fitness tracker" OR "fitness trackers" OR "fitness band" OR "fitness bands" OR "inertial" OR "inertia" OR "IMU" OR "IMUs" OR "IMU-based" OR "Movesense" OR "movisens" OR "body-worn sensor" OR "body-worn sensors" OR "body worn sensor" OR "body worn sensors" OR "motion sensor" OR "motion sensors" OR "motion tracker" OR "motion trackers" OR "motion capture")

AND ("prolonged posture" OR "prolonged postures" OR "sedentary" OR "prolonged sitting" OR "prolonged standing" OR "static posture" OR "static postures" OR "static sitting" OR "static standing" OR "sitting time" OR "standing time" OR "sedentary bout" OR "sedentary bouts" OR "sitting bout" OR "sitting bouts" OR "standing bout" OR "standing bouts" OR "posture bout" OR "posture bouts" OR "uninterrupted sitting" OR "uninterrupted standing" OR "sustained sitting" OR "sustained standing" OR "sustained posture" OR "bout duration" OR "bout durations" OR "bout length" OR "posture duration" OR "posture durations" OR "sit-to-stand" OR "sit to stand" OR "stand-to-sit" OR "stand to sit" OR "posture transition" OR "posture change" OR "posture monitoring" OR "posture detection" OR "posture classification" OR "posture recognition" OR "sitting posture" OR "sitting postures" OR "standing posture" OR "standing postures")

AND ("artificial intelligence" OR "AI" OR "machine learning" OR "deep learning" OR "supervised learning" OR "unsupervised learning" OR "reinforcement learning" OR "transfer learning" OR "neural network" OR "neural networks" OR "NN" OR "NNs" OR "CNN" OR "CNNs" OR "RNN" OR "RNNs" OR "LSTM" OR "DNN" OR "DNNs" OR "transformer" OR "support vector machine" OR "SVM" OR "decision tree" OR "decision trees" OR "random forest" OR "random forests" OR "XGBoost" OR "CatBoost" OR "AdaBoost" OR "KNN" OR "k-nearest neighbors" OR "Naive Bayes" OR "feature extraction" OR "feature engineering" OR "dimension reduction" OR "Principal Component Analysis" OR "PCA" OR "linear discriminant" OR "LDA" OR "data preprocessing" OR "data processing" OR "signal processing" OR "data analysis" OR "data analyses" OR "classification" OR "classifier" OR "regression" OR "regressor" OR "regressors" OR "prediction" OR "predictor" OR "predictors" OR "pattern analysis" OR "pattern analyses" OR "time-series analysis" OR "time-series analyses" OR "statistical model" OR "statistical learning" OR "threshold" OR "threshold-based" OR "rule-based" OR "real-time analysis" OR "real-time analyses" OR "algorithm" OR "algorithms"))

Number of records identified: 4073.

Catch-up search on Apr 8, 2026

Queries:

(TITLE-ABS("accelerome*" OR "acceleration" OR "actigraph*" OR "activPAL" OR "Axivity" OR "GENEActiv" OR "Xsens" OR "Shimmer" OR "Fitbit" OR "Garmin" OR "Polar" OR "Apple Watch" OR "wearable*" OR "smartwatch" OR "smart watch" OR "activity tracker" OR "activity trackers" OR "fitness tracker" OR "fitness trackers" OR "fitness band" OR "fitness bands" OR "inertial" OR "inertia" OR "IMU" OR "IMUs" OR "IMU-based" OR "Movesense" OR "movisens" OR "body-worn sensor" OR "body-worn sensors" OR "body worn sensor" OR "body worn sensors" OR "motion sensor" OR "motion sensors" OR "motion tracker" OR "motion trackers" OR "motion capture") OR AUTHKEY("accelerome*" OR "acceleration" OR "actigraph*" OR "activPAL" OR "Axivity" OR "GENEActiv" OR "Xsens" OR "Shimmer" OR "Fitbit" OR "Garmin" OR "Polar" OR "Apple Watch" OR "wearable*" OR "smartwatch" OR "smart watch" OR "activity tracker" OR "activity trackers" OR "fitness tracker" OR "fitness trackers" OR "fitness band" OR "fitness bands" OR "inertial" OR "inertia" OR "IMU" OR "IMUs" OR "IMU-based" OR "Movesense" OR "movisens" OR "body-worn sensor" OR "body-worn sensors" OR "body worn sensor" OR "body worn sensors" OR "motion sensor" OR "motion sensors" OR "motion tracker" OR "motion trackers" OR "motion capture"))

AND (TITLE-ABS ("prolonged posture" OR "prolonged postures" OR "sedentary" OR "prolonged sitting" OR "prolonged standing" OR "static posture" OR "static postures" OR "static sitting" OR "static standing" OR "sitting time" OR "standing time" OR "sedentary bout" OR "sedentary bouts" OR "sitting bout" OR "sitting bouts" OR "standing bout" OR "standing bouts" OR "posture bout" OR "posture bouts" OR "uninterrupted sitting" OR "uninterrupted standing" OR "sustained sitting" OR "sustained standing" OR "sustained posture" OR "bout duration" OR "bout durations" OR "bout length" OR "posture duration" OR "posture durations" OR "sit-to-stand" OR "sit to stand" OR "stand-to-sit" OR "stand to sit" OR "posture transition" OR "posture change" OR "posture monitoring" OR "posture detection" OR "posture classification" OR "posture recognition" OR "sitting posture" OR "sitting postures" OR "standing posture" OR "standing postures") OR AUTHKEY ("prolonged posture" OR "prolonged postures" OR "sedentary" OR "prolonged sitting" OR "prolonged standing" OR "static posture" OR "static postures" OR "static sitting" OR "static standing" OR "sitting time" OR "standing time" OR "sedentary bout" OR "sedentary bouts" OR "sitting bout" OR "sitting bouts" OR "standing bout" OR "standing bouts" OR "posture bout" OR "posture bouts" OR "uninterrupted sitting" OR "uninterrupted standing" OR "sustained sitting" OR "sustained standing" OR "sustained posture" OR "bout duration" OR "bout durations" OR "bout length" OR "posture duration" OR "posture durations" OR "sit-to-stand" OR "sit to stand" OR "stand-to-sit" OR "stand to sit" OR "posture transition" OR "posture change" OR "posture monitoring" OR "posture detection" OR "posture classification" OR "posture recognition" OR "sitting posture" OR "sitting postures" OR "standing posture" OR "standing postures"))

AND (TITLE-ABS("artificial intelligence" OR "AI" OR "machine learning" OR "deep learning" OR "supervised learning" OR "unsupervised learning" OR "reinforcement learning" OR "transfer learning" OR "neural network" OR "neural networks" OR "NN" OR "NNs" OR "CNN" OR "CNNs" OR "RNN" OR "RNNs" OR "LSTM" OR "DNN" OR "DNNs" OR "transformer" OR "support vector machine" OR "SVM" OR "decision tree" OR "decision trees" OR "random forest" OR "random forests" OR "XGBoost" OR "CatBoost" OR "AdaBoost" OR "KNN" OR "k-nearest neighbors" OR "Naive Bayes" OR "feature extraction" OR "feature engineering" OR "dimension reduction" OR "Principal Component Analysis" OR "PCA" OR "linear discriminant" OR "LDA" OR "data preprocessing" OR "data processing" OR "signal processing" OR "data analysis" OR "data analyses" OR "classification" OR "classifier" OR "regression" OR "regressor" OR "regressors" OR "prediction" OR "predictor" OR "predictors" OR "pattern analysis" OR "pattern analyses" OR "time-series analysis" OR "time-series analyses" OR "statistical model" OR "statistical learning"

OR "threshold" OR "threshold-based" OR "rule-based" OR "real-time analysis" OR "real-time analyses" OR "algorithm" OR "algorithms") OR AUTHKEY("artificial intelligence" OR "AI" OR "machine learning" OR "deep learning" OR "supervised learning" OR "unsupervised learning" OR "reinforcement learning" OR "transfer learning" OR "neural network" OR "neural networks" OR "NN" OR "NNs" OR "CNN" OR "CNNs" OR "RNN" OR "RNNs" OR "LSTM" OR "DNN" OR "DNNs" OR "transformer" OR "support vector machine" OR "SVM" OR "decision tree" OR "decision trees" OR "random forest" OR "random forests" OR "XGBoost" OR "CatBoost" OR "AdaBoost" OR "KNN" OR "k-nearest neighbors" OR "Naive Bayes" OR "feature extraction" OR "feature engineering" OR "dimension reduction" OR "Principal Component Analysis" OR "PCA" OR "linear discriminant" OR "LDA" OR "data preprocessing" OR "data processing" OR "signal processing" OR "data analysis" OR "data analyses" OR "classification" OR "classifier" OR "regression" OR "regressor" OR "regressors" OR "prediction" OR "predictor" OR "predictors" OR "pattern analysis" OR "pattern analyses" OR "time-series analysis" OR "time-series analyses" OR "statistical model" OR "statistical learning" OR "threshold" OR "threshold-based" OR "rule-based" OR "real-time analysis" OR "real-time analyses" OR "algorithm" OR "algorithms"))

Number of records identified: 4561.

Revision of Boolean logic structure:

- Previous structure used on Jan 12, 2025 and Dec 4, 2025:
  TITLE-ABS(concept 1 AND concept 2 AND concept 3)
  OR AUTHKEY(concept 1 AND concept 2 AND concept 3)
- Revised structure used on Apr 8, 2026:
  (TITLE-ABS(concept 1) OR AUTHKEY(concept 1))
  AND (TITLE-ABS(concept 2) OR AUTHKEY(concept 2))
  AND (TITLE-ABS(concept 3) OR AUTHKEY(concept 3))
- Rationale: The revised structure includes a greater number of results and ensures consistency with the search logic used in other databases.
- The search terms are same as December 4, 2025.

ProQuest Central

Limit to full text AND peer-reviewed.

Initial search on Jan 12, 2025

Queries: (TI("inertial" OR "inertia" OR "IMU" OR "IMUs" OR "wearable*" OR "actigraph*" OR "accelerome*" OR "acceleration" OR "magnetometer" OR "magnetometers" OR "gyroscope" OR "gyroscopes" OR "activity tracker" OR "activity trackers" OR "fitness tracker" OR "fitness trackers" OR "fitness band" OR "fitness bands" OR "body-worn sensor" OR "body-worn sensors" OR "body worn sensor" OR "body worn sensors" OR "motion sensor" OR "motion sensors" OR "motion tracker" OR "motion trackers" OR "motion capture" OR "smartwatch" OR "smart watch”) OR AB("inertial" OR "inertia" OR "IMU" OR "IMUs" OR "wearable*" OR "actigraph*" OR "accelerome*" OR "acceleration" OR "magnetometer" OR "magnetometers" OR "gyroscope" OR "gyroscopes" OR "activity tracker" OR "activity trackers" OR "fitness tracker" OR "fitness trackers" OR "fitness band" OR "fitness bands" OR "body-worn sensor" OR "body-worn sensors" OR "body worn sensor" OR "body worn sensors" OR "motion sensor" OR "motion sensors" OR

"motion tracker" OR "motion trackers" OR "motion capture" OR "smartwatch" OR "smart watch”))

AND (TI("prolonged posture" OR "prolonged postures" OR "sedentary" OR "prolonged sitting" OR "prolonged standing" OR "sitting time" OR "sitting posture" OR "sitting postures" OR "standing time" OR "standing posture" OR "standing postures" OR "static posture" OR "static postures" OR "static sitting" OR "static standing") OR AB("prolonged posture" OR "prolonged postures" OR "sedentary" OR "prolonged sitting" OR "prolonged standing" OR "sitting time" OR "sitting posture" OR "sitting postures" OR "standing time" OR "standing posture" OR "standing postures" OR "static posture" OR "static postures" OR "static sitting" OR "static standing"))

AND (TI("machine learning" OR "deep learning" OR "neural network" OR "neural networks" OR "NN" OR "NNs" OR "CNN" OR "CNNs" OR "RNN" OR "RNNs" OR "DNN" OR "DNNs" OR "KNN" OR "k-nearest neighbors" OR "SVM" OR "support vector machine" OR "LSTM" OR "XGBoost" OR "catboost" OR "adaboost" OR "decision tree" OR "decision trees" OR "random forest" OR "random forests" OR "supervised" OR "unsupervised" OR "Naive Bayes" OR " Principal Component Analysis" OR "PCA" OR "dimension reduction" OR "linear discriminate" OR "LDA" OR "transformer" OR "reinforcement learning" OR "computer vision" OR "artificial intelligence" OR "AI" OR "unsupervised learning" OR "supervised learning" OR "data analysis" OR "data analyses" OR "feature extraction" OR "data preprocessing" OR "data processing" OR "signal processing" OR "information processing" OR "feature engineering" OR "statistical model" OR "ANOVA" OR "algorithm" OR "algorithms" OR "classification" OR "classifier" OR "time-series analysis" OR "time-series analyses" OR "real-time analysis" OR "real-time analyses" OR "sentiment analysis" OR "sentiment analyses" OR "pattern analysis" OR "pattern analyses" OR "prediction" OR "predictor" OR "predictors" OR "regression" OR "regressor" OR "regressors") OR AB("machine learning" OR "deep learning" OR "neural network" OR "neural networks" OR "NN" OR "NNs" OR "CNN" OR "CNNs" OR "RNN" OR "RNNs" OR "DNN" OR "DNNs" OR "KNN" OR "k-nearest neighbors" OR "SVM" OR "support vector machine" OR "LSTM" OR "XGBoost" OR "catboost" OR "adaboost" OR "decision tree" OR "decision trees" OR "random forest" OR "random forests" OR "supervised" OR "unsupervised" OR "Naive Bayes" OR " Principal Component Analysis" OR "PCA" OR "dimension reduction" OR "linear discriminate" OR "LDA" OR "transformer" OR "reinforcement learning" OR "computer vision" OR "artificial intelligence" OR "AI" OR "unsupervised learning" OR "supervised learning" OR "data analysis" OR "data analyses" OR "feature extraction" OR "data preprocessing" OR "data processing" OR "signal processing" OR "information processing" OR "feature engineering" OR "statistical model" OR "ANOVA" OR "algorithm" OR "algorithms" OR "classification" OR "classifier" OR "time-series analysis" OR "time-series analyses" OR "real-time analysis" OR "real-time analyses" OR "sentiment analysis" OR "sentiment analyses" OR "pattern analysis" OR "pattern analyses" OR "prediction" OR "predictor" OR "predictors" OR "regression" OR "regressor" OR "regressors"))

Number of records identified: 985.

Updated search on Dec 4, 2025

Queries: (TI("accelerome*" OR "acceleration" OR "actigraph*" OR "activPAL" OR "Axivity" OR "GENEActiv" OR "Xsens" OR "Shimmer" OR "Fitbit" OR "Garmin" OR "Polar" OR "Apple Watch" OR "wearable*" OR "smartwatch" OR "smart watch" OR "activity tracker" OR "activity trackers" OR "fitness tracker" OR "fitness trackers" OR "fitness band" OR "fitness bands" OR "inertial"

OR "inertia" OR "IMU" OR "IMUs" OR "IMU-based" OR "Movesense" OR "movisens" OR "body-worn sensor" OR "body-worn sensors" OR "body worn sensor" OR "body worn sensors" OR "motion sensor" OR "motion sensors" OR "motion tracker" OR "motion trackers" OR "motion capture") OR AB("accelerome*" OR "acceleration" OR "actigraph*" OR "activPAL" OR "Axivity" OR "GENEActiv" OR "Xsens" OR "Shimmer" OR "Fitbit" OR "Garmin" OR "Polar" OR "Apple Watch" OR "wearable*" OR "smartwatch" OR "smart watch" OR "activity tracker" OR "activity trackers" OR "fitness tracker" OR "fitness trackers" OR "fitness band" OR "fitness bands" OR "inertial" OR "inertia" OR "IMU" OR "IMUs" OR "IMU-based" OR "Movesense" OR "movisens" OR "body-worn sensor" OR "body-worn sensors" OR "body worn sensor" OR "body worn sensors" OR "motion sensor" OR "motion sensors" OR "motion tracker" OR "motion trackers" OR "motion capture"))

AND (TI("prolonged posture" OR "prolonged postures" OR "sedentary" OR "prolonged sitting" OR "prolonged standing" OR "static posture" OR "static postures" OR "static sitting" OR "static standing" OR "sitting time" OR "standing time" OR "sedentary bout" OR "sedentary bouts" OR "sitting bout" OR "sitting bouts" OR "standing bout" OR "standing bouts" OR "posture bout" OR "posture bouts" OR "uninterrupted sitting" OR "uninterrupted standing" OR "sustained sitting" OR "sustained standing" OR "sustained posture" OR "bout duration" OR "bout durations" OR "bout length" OR "posture duration" OR "posture durations" OR "sit-to-stand" OR "sit to stand" OR "stand-to-sit" OR "stand to sit" OR "posture transition" OR "posture change" OR "posture monitoring" OR "posture detection" OR "posture classification" OR "posture recognition" OR "sitting posture" OR "sitting postures" OR "standing posture" OR "standing postures") OR AB("prolonged posture" OR "prolonged postures" OR "sedentary" OR "prolonged sitting" OR "prolonged standing" OR "static posture" OR "static postures" OR "static sitting" OR "static standing" OR "sitting time" OR "standing time" OR "sedentary bout" OR "sedentary bouts" OR "sitting bout" OR "sitting bouts" OR "standing bout" OR "standing bouts" OR "posture bout" OR "posture bouts" OR "uninterrupted sitting" OR "uninterrupted standing" OR "sustained sitting" OR "sustained standing" OR "sustained posture" OR "bout duration" OR "bout durations" OR "bout length" OR "posture duration" OR "posture durations" OR "sit-to-stand" OR "sit to stand" OR "stand-to-sit" OR "stand to sit" OR "posture transition" OR "posture change" OR "posture monitoring" OR "posture detection" OR "posture classification" OR "posture recognition" OR "sitting posture" OR "sitting postures" OR "standing posture" OR "standing postures"))

AND (TI("artificial intelligence" OR "AI" OR "machine learning" OR "deep learning" OR "supervised learning" OR "unsupervised learning" OR "reinforcement learning" OR "transfer learning" OR "neural network" OR "neural networks" OR "NN" OR "NNs" OR "CNN" OR "CNNs" OR "RNN" OR "RNNs" OR "LSTM" OR "DNN" OR "DNNs" OR "transformer" OR "support vector machine" OR "SVM" OR "decision tree" OR "decision trees" OR "random forest" OR "random forests" OR "XGBoost" OR "CatBoost" OR "AdaBoost" OR "KNN" OR "k-nearest neighbors" OR "Naive Bayes" OR "feature extraction" OR "feature engineering" OR "dimension reduction" OR "Principal Component Analysis" OR "PCA" OR "linear discriminant" OR "LDA" OR "data preprocessing" OR "data processing" OR "signal processing" OR "data analysis" OR "data analyses" OR "classification" OR "classifier" OR "regression" OR "regressor" OR "regressors" OR "prediction" OR "predictor" OR "predictors" OR "pattern analysis" OR "pattern analyses" OR "time-series analysis" OR "time-series analyses" OR "statistical model" OR "statistical learning" OR "threshold" OR "threshold-based" OR "rule-based" OR "real-time analysis" OR "real-time analyses" OR "algorithm" OR "algorithms") OR AB("artificial intelligence" OR "AI" OR "machine learning" OR "deep learning" OR "supervised learning" OR "unsupervised learning" OR "reinforcement learning" OR "transfer learning" OR "neural network" OR "neural networks"

OR "NN" OR "NNs" OR "CNN" OR "CNNs" OR "RNN" OR "RNNs" OR "LSTM" OR "DNN" OR "DNNs" OR "transformer" OR "support vector machine" OR "SVM" OR "decision tree" OR "decision trees" OR "random forest" OR "random forests" OR "XGBoost" OR "CatBoost" OR "AdaBoost" OR "KNN" OR "k-nearest neighbors" OR "Naive Bayes" OR "feature extraction" OR "feature engineering" OR "dimension reduction" OR "Principal Component Analysis" OR "PCA" OR "linear discriminant" OR "LDA" OR "data preprocessing" OR "data processing" OR "signal processing" OR "data analysis" OR "data analyses" OR "classification" OR "classifier" OR "regression" OR "regressor" OR "regressors" OR "prediction" OR "predictor" OR "predictors" OR "pattern analysis" OR "pattern analyses" OR "time-series analysis" OR "time-series analyses" OR "statistical model" OR "statistical learning" OR "threshold" OR "threshold-based" OR "rule-based" OR "real-time analysis" OR "real-time analyses" OR "algorithm" OR "algorithms"))

Number of records identified: 1309.

Catch-up search on Apr 8, 2026

Queries: same as December 4, 2025.

Number of records identified: 1414.

PubMed

No additional filter applied during database searching.

Initial search on Jan 12, 2025

Queries: ("Wearable Electronic Devices"[MeSH] OR "Accelerometry"[MeSH] OR "inertial"[tiab] OR "inertia”[tiab] OR "IMU"[tiab] OR "IMUs"[tiab] OR "wearable*"[tiab] OR "actigraph*"[tiab] OR "accelerome*"[tiab] OR "acceleration"[tiab] OR "magnetometer"[tiab] OR "magnetometers"[tiab] OR "gyroscope"[tiab] OR "gyroscopes"[tiab] OR "activity tracker"[tiab] OR "activity trackers"[tiab] OR "fitness tracker"[tiab] OR "fitness trackers"[tiab] OR "fitness band"[tiab] OR "fitness bands”[tiab] OR "body-worn sensor"[tiab] OR "body-worn sensors”[tiab] OR "body worn sensor”[tiab] OR "body worn sensors”[tiab] OR "motion sensor"[tiab] OR "motion sensors”[tiab] OR "motion tracker”[tiab] OR "motion trackers”[tiab] OR "motion capture"[tiab] OR "smartwatch”[tiab] OR "smart watch”[tiab]) AND ("Sedentary Behavior"[Mesh] OR "prolonged posture"[tiab] OR "prolonged postures”[tiab] OR "sedentary"[tiab] OR "prolonged sitting"[tiab] OR "prolonged standing"[tiab] OR "sitting time"[tiab] OR "sitting posture”[tiab] OR "sitting postures”[tiab] OR "standing time”[tiab] OR "standing posture”[tiab] OR "standing postures”[tiab] OR "static posture"[tiab] OR "static postures"[tiab] OR "static sitting"[tiab] OR "static standing"[tiab]) AND ("Electronic Data Processing"[Mesh] OR "Data Analysis"[Mesh] OR "Algorithms"[Mesh] OR "machine learning"[tiab] OR "deep learning"[tiab] OR "neural network"[tiab] OR "neural networks"[tiab] OR "NN"[tiab] OR "NNs"[tiab] OR "CNN"[tiab] OR "CNNs"[tiab] OR "RNN"[tiab] OR "RNNs"[tiab] OR "DNN"[tiab] OR "DNNs"[tiab] OR "KNN"[tiab] OR "k-nearest neighbors"[tiab] OR "SVM"[tiab] OR "support vector machine"[tiab] OR "LSTM"[tiab] OR "XGBoost"[tiab] OR "catboost"[tiab] OR "adaboost"[tiab] OR "decision tree"[tiab] OR "decision trees"[tiab] OR "random forest"[tiab] OR "random forests"[tiab] OR "supervised"[tiab] OR "unsupervised"[tiab] OR "Naive Bayes"[tiab] OR

"Principal Component Analysis"[tiab] OR "PCA"[tiab] OR "dimension reduction"[tiab] OR "linear discriminate"[tiab] OR "LDA"[tiab] OR "transformer"[tiab] OR "reinforcement learning"[tiab] OR "computer vision"[tiab] OR "artificial intelligence"[tiab] OR "AI"[tiab] OR "unsupervised learning"[tiab] OR "supervised learning"[tiab] OR "data analysis"[tiab] OR "data analyses"[tiab] OR "feature extraction"[tiab] OR "data preprocessing"[tiab] OR "data processing"[tiab] OR "signal processing"[tiab] OR "information processing"[tiab] OR "feature engineering"[tiab] OR "statistical model"[tiab] OR "ANOVA"[tiab] OR "algorithm"[tiab] OR "algorithms"[tiab] OR "classification"[tiab] OR "classifier"[tiab] OR "time-series analysis"[tiab] OR "time-series analyses"[tiab] OR "real-time analysis"[tiab] OR "real-time analyses"[tiab] OR "sentiment analysis"[tiab] OR "sentiment analyses"[tiab] OR "pattern analysis"[tiab] OR "pattern analyses"[tiab] OR "prediction"[tiab] OR "predictor"[tiab] OR "predictors"[tiab] OR "regression"[tiab] OR "regressor"[tiab] OR "regressors"[tiab])

Number of records identified: 2628.

Updated search on Dec 4, 2025

Queries: ("Wearable Electronic Devices"[Mesh] OR "Accelerometry"[Mesh] OR "accelerome*"[tiab] OR "acceleration"[tiab] OR "actigraph*"[tiab] OR "activPAL"[tiab] OR "Axivity"[tiab] OR "GENEActiv"[tiab] OR "Xsens"[tiab] OR "Shimmer"[tiab] OR "Fitbit"[tiab] OR "Garmin"[tiab] OR "Polar"[tiab] OR "Apple Watch"[tiab] OR "smart watch"[tiab] OR "smartwatch*"[tiab] OR "wearable*"[tiab] OR "activity tracker*"[tiab] OR "fitness tracker*"[tiab] OR "fitness band*"[tiab] OR "inertial"[tiab] OR "inertia"[tiab] OR "IMU"[tiab] OR "IMUs"[tiab] OR "IMU-based"[tiab] OR "Movesense"[tiab] OR "movisens"[tiab] OR "body-worn sensor*"[tiab] OR "body worn sensor*"[tiab] OR "motion sensor*"[tiab] OR "motion tracker*"[tiab] OR "motion capture"[tiab]) AND ("Sedentary Behavior"[Mesh] OR "prolonged posture"[tiab] OR "prolonged postures"[tiab] OR "prolonged sitting"[tiab] OR "prolonged standing"[tiab] OR "sedentary"[tiab] OR "static posture"[tiab] OR "static postures"[tiab] OR "static sitting"[tiab] OR "static standing"[tiab] OR "sitting time"[tiab] OR "standing time"[tiab] OR "sedentary bout"[tiab] OR "sedentary bouts"[tiab] OR "sitting bout"[tiab] OR "sitting bouts"[tiab] OR "standing bout"[tiab] OR "standing bouts"[tiab] OR "uninterrupted sitting"[tiab] OR "sustained sitting"[tiab] OR "sustained standing"[tiab] OR "sustained posture"[tiab] OR "bout duration"[tiab] OR "bout durations"[tiab] OR "bout length"[tiab] OR "posture duration"[tiab] OR "sit-to-stand"[tiab] OR "sit to stand"[tiab] OR "stand-to-sit"[tiab] OR "stand to sit"[tiab] OR "posture transition"[tiab] OR "posture change"[tiab] OR "posture monitoring"[tiab] OR "posture detection"[tiab] OR "posture classification"[tiab] OR "posture recognition"[tiab] OR "sitting posture"[tiab] OR "sitting postures"[tiab] OR "standing posture"[tiab] OR "standing postures"[tiab]) AND ("Electronic Data Processing"[Mesh] OR "Data Analysis"[Mesh] OR "Algorithms"[Mesh] OR "artificial intelligence"[tiab] OR "AI"[tiab] OR "machine learning"[tiab] OR "deep learning"[tiab] OR "supervised learning"[tiab] OR "unsupervised learning"[tiab] OR "reinforcement learning"[tiab] OR "transfer learning"[tiab] OR "neural network"[tiab] OR "neural networks"[tiab] OR "CNN"[tiab] OR "RNN"[tiab] OR "LSTM"[tiab] OR "DNN"[tiab] OR "transformer"[tiab] OR "support vector machine"[tiab] OR "SVM"[tiab] OR "decision tree"[tiab] OR "decision trees"[tiab] OR "random forest"[tiab] OR "random forests"[tiab] OR "XGBoost"[tiab] OR "CatBoost"[tiab] OR "AdaBoost"[tiab] OR "KNN"[tiab] OR "k-nearest neighbors"[tiab] OR "Naive Bayes"[tiab] OR "feature extraction"[tiab] OR "feature engineering"[tiab] OR "dimension reduction"[tiab] OR "Principal Component Analysis"[tiab] OR "PCA"[tiab] OR "linear discriminant"[tiab] OR "LDA"[tiab] OR "data preprocessing"[tiab] OR "data processing"[tiab] OR "signal

processing"[tiab] OR "data analysis"[tiab] OR "data analyses"[tiab] OR "classification"[tiab] OR "classifier"[tiab] OR "regression"[tiab] OR "regressor"[tiab] OR "regressors"[tiab] OR "prediction"[tiab] OR "predictor"[tiab] OR "predictors"[tiab] OR "pattern analysis"[tiab] OR "pattern analyses"[tiab] OR "time-series analysis"[tiab] OR "time-series analyses"[tiab] OR "statistical model"[tiab] OR "statistical learning"[tiab] OR "threshold"[tiab] OR "threshold-based"[tiab] OR "rule-based"[tiab] OR "real-time analysis"[tiab] OR "real-time analyses"[tiab] OR "algorithm"[tiab] OR "algorithms"[tiab])

Remark: even though "posture bout", "posture bouts", "uninterrupted standing", and "posture durations" were not found in the phrase index, they were kept in the search query for consistency across databases.

Number of records identified: 3270.

Catch-up search on Apr 8, 2026

Queries: same as December 4, 2025.

Number of records identified: 3407.

Embase

The results are restricted to "not conference abstract”. The restriction is added to the end of the queries, no additional filter applied during database searching.

Initial search on Jan 12, 2025

Queries:

#1 'wearable device'/exp OR 'accelerometry'/exp OR 'inertial':ti,ab,kw OR 'inertia':ti,ab,kw OR 'imu':ti,ab,kw OR 'imus':ti,ab,kw OR 'wearable*':ti,ab,kw OR 'actigraph*':ti,ab,kw OR 'accelerome*':ti,ab,kw OR 'acceleration':ti,ab,kw OR 'magnetometer':ti,ab,kw OR 'magnetometers':ti,ab,kw OR 'gyroscope':ti,ab,kw OR 'gyroscopes':ti,ab,kw OR 'activity tracker':ti,ab,kw OR 'activity trackers':ti,ab,kw OR 'fitness tracker':ti,ab,kw OR 'fitness trackers':ti,ab,kw OR 'fitness band':ti,ab,kw OR 'fitness bands':ti,ab,kw OR 'body-worn sensor':ti,ab,kw OR 'body-worn sensors':ti,ab,kw OR 'body worn sensor':ti,ab,kw OR 'body worn sensors':ti,ab,kw OR 'motion sensor':ti,ab,kw OR 'motion sensors':ti,ab,kw OR 'motion tracker':ti,ab,kw OR 'motion trackers':ti,ab,kw OR 'motion capture':ti,ab,kw OR 'smartwatch':ti,ab,kw OR 'smart watch':ti,ab,kw

#2 'sedentary lifestyle'/exp OR 'sitting'/exp OR 'standing'/exp OR 'prolonged posture':ti,ab,kw OR 'prolonged postures':ti,ab,kw OR 'sedentary':ti,ab,kw OR 'prolonged sitting':ti,ab,kw OR 'prolonged standing':ti,ab,kw OR 'sitting time':ti,ab,kw OR 'sitting posture':ti,ab,kw OR 'sitting postures':ti,ab,kw OR 'standing time':ti,ab,kw OR 'standing posture':ti,ab,kw OR 'standing postures':ti,ab,kw OR 'static posture':ti,ab,kw OR 'static postures':ti,ab,kw OR 'static sitting':ti,ab,kw OR 'static standing':ti,ab,kw

#3 'data processing'/exp OR 'data analysis'/exp OR 'algorithm'/exp OR 'machine learning':ti,ab,kw OR 'deep learning':ti,ab,kw OR 'neural network':ti,ab,kw OR 'neural networks':ti,ab,kw OR 'nn':ti,ab,kw OR 'nns':ti,ab,kw OR 'cnn':ti,ab,kw OR 'cnns':ti,ab,kw OR

'rnn':ti,ab,kw OR 'rnns':ti,ab,kw OR 'dnn':ti,ab,kw OR 'dnns':ti,ab,kw OR 'knn':ti,ab,kw OR 'k-nearest neighbors':ti,ab,kw OR 'svm':ti,ab,kw OR 'support vector machine':ti,ab,kw OR 'lstm':ti,ab,kw OR 'xgboost':ti,ab,kw OR 'catboost':ti,ab,kw OR 'adaboost':ti,ab,kw OR 'decision tree':ti,ab,kw OR 'decision trees':ti,ab,kw OR 'random forest':ti,ab,kw OR 'random forests':ti,ab,kw OR 'supervised':ti,ab,kw OR 'unsupervised':ti,ab,kw OR 'naive bayes':ti,ab,kw OR 'principal component analysis':ti,ab,kw OR 'pca':ti,ab,kw OR 'dimension reduction':ti,ab,kw OR 'linear discriminate':ti,ab,kw OR 'lda':ti,ab,kw OR 'transformer':ti,ab,kw OR 'reinforcement learning':ti,ab,kw OR 'computer vision':ti,ab,kw OR 'artificial intelligence':ti,ab,kw OR 'ai':ti,ab,kw OR 'unsupervised learning':ti,ab,kw OR 'supervised learning':ti,ab,kw OR 'data analysis':ti,ab,kw OR 'data analyses':ti,ab,kw OR 'feature extraction':ti,ab,kw OR 'data preprocessing':ti,ab,kw OR 'data processing':ti,ab,kw OR 'signal processing':ti,ab,kw OR 'information processing':ti,ab,kw OR 'feature engineering':ti,ab,kw OR 'statistical model':ti,ab,kw OR 'anova':ti,ab,kw OR 'algorithm':ti,ab,kw OR 'algorithms':ti,ab,kw OR 'classification':ti,ab,kw OR 'classifier':ti,ab,kw OR 'time-series analysis':ti,ab,kw OR 'time-series analyses':ti,ab,kw OR 'real-time analysis':ti,ab,kw OR 'real-time analyses':ti,ab,kw OR 'sentiment analysis':ti,ab,kw OR 'sentiment analyses':ti,ab,kw OR 'pattern analysis':ti,ab,kw OR 'pattern analyses':ti,ab,kw OR 'prediction':ti,ab,kw OR 'predictor':ti,ab,kw OR 'predictors':ti,ab,kw OR 'regression':ti,ab,kw OR 'regressor':ti,ab,kw OR 'regressors':ti,ab,kw

#4 #1 AND #2 AND #3 NOT 'conference abstract':it

Number of records identified: 4268.

Updated search on Dec 4, 2025

Queries:

#1 'wearable device'/exp OR 'accelerometry'/exp OR 'inertial measurement unit'/exp OR 'actigraph'/exp OR 'accelerome*':ti,ab,kw OR 'acceleration':ti,ab,kw OR 'actigraph*':ti,ab,kw OR 'activpal':ti,ab,kw OR 'axivity':ti,ab,kw OR 'geneactiv':ti,ab,kw OR 'xsens':ti,ab,kw OR 'shimmer':ti,ab,kw OR 'fitbit':ti,ab,kw OR 'garmin':ti,ab,kw OR 'polar':ti,ab,kw OR 'apple watch':ti,ab,kw OR 'smart watch':ti,ab,kw OR 'smartwatch*':ti,ab,kw OR 'wearable*':ti,ab,kw OR 'imu':ti,ab,kw OR 'imus':ti,ab,kw OR 'imu-based':ti,ab,kw OR 'inertial':ti,ab,kw OR 'inertia':ti,ab,kw OR 'movesense':ti,ab,kw OR 'movisens':ti,ab,kw OR 'body-worn sensor*':ti,ab,kw OR 'body worn sensor*':ti,ab,kw OR 'motion sensor*':ti,ab,kw OR 'motion tracker*':ti,ab,kw OR 'motion capture':ti,ab,kw OR 'activity tracker*':ti,ab,kw OR 'fitness tracker*':ti,ab,kw OR 'fitness band*':ti,ab,kw

#2 'sedentary behavior'/exp OR 'sedentary lifestyle'/exp OR 'sitting'/exp OR 'standing'/exp OR 'posture'/exp OR 'prolonged posture':ti,ab,kw OR 'prolonged postures':ti,ab,kw OR 'prolonged sitting':ti,ab,kw OR 'prolonged standing':ti,ab,kw OR 'sedentary':ti,ab,kw OR 'sedentary bout*':ti,ab,kw OR 'sitting bout*':ti,ab,kw OR 'standing bout*':ti,ab,kw OR 'posture bout*':ti,ab,kw OR 'static posture':ti,ab,kw OR 'static postures':ti,ab,kw OR 'static sitting':ti,ab,kw OR 'static standing':ti,ab,kw OR 'uninterrupted sitting':ti,ab,kw OR 'uninterrupted standing':ti,ab,kw OR 'sustained sitting':ti,ab,kw OR 'sustained standing':ti,ab,kw OR 'sustained posture':ti,ab,kw OR 'bout duration':ti,ab,kw OR 'bout durations':ti,ab,kw OR 'bout length':ti,ab,kw OR 'posture duration':ti,ab,kw OR 'posture durations':ti,ab,kw OR 'sit-to-stand':ti,ab,kw OR 'sit to stand':ti,ab,kw OR 'stand-to-sit':ti,ab,kw OR 'stand to sit':ti,ab,kw OR 'posture transition':ti,ab,kw OR 'posture

change':ti,ab,kw OR 'posture monitoring':ti,ab,kw OR 'posture detection':ti,ab,kw OR 'posture classification':ti,ab,kw OR 'posture recognition':ti,ab,kw OR 'sitting posture':ti,ab,kw OR 'sitting postures':ti,ab,kw OR 'standing posture':ti,ab,kw OR 'standing postures':ti,ab,kw

#3 'data processing'/exp OR 'information processing'/exp OR 'data analysis'/exp OR 'algorithm'/exp OR 'machine learning'/exp OR 'artificial intelligence'/exp OR 'machine learning':ti,ab,kw OR 'deep learning':ti,ab,kw OR 'neural network*':ti,ab,kw OR 'cnn':ti,ab,kw OR 'cnns':ti,ab,kw OR 'rnn':ti,ab,kw OR 'rnns':ti,ab,kw OR 'lstm':ti,ab,kw OR 'dnn':ti,ab,kw OR 'dnns':ti,ab,kw OR 'transformer':ti,ab,kw OR 'support vector machine':ti,ab,kw OR 'svm':ti,ab,kw OR 'decision tree*':ti,ab,kw OR 'random forest*':ti,ab,kw OR 'xgboost':ti,ab,kw OR 'catboost':ti,ab,kw OR 'adaboost':ti,ab,kw OR 'knn':ti,ab,kw OR 'k-nearest neighbors':ti,ab,kw OR 'naive bayes':ti,ab,kw OR 'feature extraction':ti,ab,kw OR 'feature engineering':ti,ab,kw OR 'principal component analysis':ti,ab,kw OR 'pca':ti,ab,kw OR 'dimension reduction':ti,ab,kw OR 'linear discriminant':ti,ab,kw OR 'lda':ti,ab,kw OR 'signal processing':ti,ab,kw OR 'data preprocessing':ti,ab,kw OR 'data processing':ti,ab,kw OR 'data analysis':ti,ab,kw OR 'data analyses':ti,ab,kw OR 'classification':ti,ab,kw OR 'classifier':ti,ab,kw OR 'regression':ti,ab,kw OR 'regressor*':ti,ab,kw OR 'prediction':ti,ab,kw OR 'predictor*':ti,ab,kw OR 'pattern analysis':ti,ab,kw OR 'pattern analyses':ti,ab,kw OR 'time-series analysis':ti,ab,kw OR 'time-series analyses':ti,ab,kw OR 'statistical model':ti,ab,kw OR 'statistical learning':ti,ab,kw OR 'threshold*':ti,ab,kw OR 'rule-based':ti,ab,kw OR 'real-time analysis':ti,ab,kw OR 'real-time analyses':ti,ab,kw OR 'algorithm':ti,ab,kw OR 'algorithms':ti,ab,kw

#4 #1 AND #2 AND #3 NOT 'conference abstract'/it

Number of records identified: 8326.

Catch-up search on Apr 8, 2026

Queries: same as December 4, 2025.

Number of records identified: 8658.

CINAHL

No additional filter applied during database searching.

Initial search on Jan 12, 2025

Queries:

S1 (XB("inertial" OR "inertia" OR "IMU" OR "IMUs" OR "wearable*" OR "actigraph*" OR "accelerome*" OR "acceleration" OR "magnetometer" OR "magnetometers" OR "gyroscope" OR "gyroscopes" OR "activity tracker" OR "activity trackers" OR "fitness tracker" OR "fitness trackers" OR "fitness band" OR "fitness bands" OR "body-worn sensor" OR "body-worn sensors" OR "body worn sensor" OR "body worn sensors" OR "motion sensor" OR "motion sensors" OR "motion tracker" OR "motion trackers" OR "motion capture" OR "smartwatch" OR "smart watch”)) OR (KW("inertial" OR "inertia" OR "IMU" OR "IMUs" OR "wearable*" OR "actigraph*" OR "accelerome*" OR "acceleration" OR "magnetometer" OR "magnetometers"

OR "gyroscope" OR "gyroscopes" OR "activity tracker" OR "activity trackers" OR "fitness tracker" OR "fitness trackers" OR "fitness band" OR "fitness bands" OR "body-worn sensor" OR "body-worn sensors" OR "body worn sensor" OR "body worn sensors" OR "motion sensor" OR "motion sensors" OR "motion tracker" OR "motion trackers" OR "motion capture" OR "smartwatch" OR "smart watch”)) OR (MH("inertial" OR "inertia" OR "IMU" OR "IMUs" OR "wearable*" OR "actigraph*" OR "accelerome*" OR "acceleration" OR "magnetometer" OR "magnetometers" OR "gyroscope" OR "gyroscopes" OR "activity tracker" OR "activity trackers" OR "fitness tracker" OR "fitness trackers" OR "fitness band" OR "fitness bands" OR "body-worn sensor" OR "body-worn sensors" OR "body worn sensor" OR "body worn sensors" OR "motion sensor" OR "motion sensors" OR "motion tracker" OR "motion trackers" OR "motion capture" OR "smartwatch" OR "smart watch”))

S2 (XB("prolonged posture" OR "prolonged postures" OR "sedentary" OR "prolonged sitting" OR "prolonged standing" OR "sitting time" OR "sitting posture" OR "sitting postures" OR "standing time" OR "standing posture" OR "standing postures" OR "static posture" OR "static postures" OR "static sitting" OR "static standing”) OR (KW("prolonged posture" OR "prolonged postures" OR "sedentary" OR "prolonged sitting" OR "prolonged standing" OR "sitting time" OR "sitting posture" OR "sitting postures" OR "standing time" OR "standing posture" OR "standing postures" OR "static posture" OR "static postures" OR "static sitting" OR "static standing”)) OR (MH("prolonged posture" OR "prolonged postures" OR "sedentary" OR "prolonged sitting" OR "prolonged standing" OR "sitting time" OR "sitting posture" OR "sitting postures" OR "standing time" OR "standing posture" OR "standing postures" OR "static posture" OR "static postures" OR "static sitting" OR "static standing”))

S3 (XB("machine learning" OR "deep learning" OR "neural network" OR "neural networks" OR "NN" OR "NNs" OR "CNN" OR "CNNs" OR "RNN" OR "RNNs" OR "DNN" OR "DNNs" OR "KNN" OR "k-nearest neighbors" OR "SVM" OR "support vector machine" OR "LSTM" OR "XGBoost" OR "catboost" OR "adaboost" OR "decision tree" OR "decision trees" OR "random forest" OR "random forests" OR "supervised" OR "unsupervised" OR "Naive Bayes" OR "Principal Component Analysis" OR "PCA" OR "dimension reduction" OR "linear discriminate" OR "LDA" OR "transformer" OR "reinforcement learning" OR "computer vision" OR "artificial intelligence" OR "AI" OR "unsupervised learning" OR "supervised learning" OR "data analysis" OR "data analyses" OR "feature extraction" OR "data preprocessing" OR "data processing" OR "signal processing" OR "information processing" OR "feature engineering" OR "statistical model" OR "ANOVA" OR "algorithm" OR "algorithms" OR "classification" OR "classifier" OR "time-series analysis" OR "time-series analyses" OR "real-time analysis" OR "real-time analyses" OR "sentiment analysis" OR "sentiment analyses" OR "pattern analysis" OR "pattern analyses" OR "prediction" OR "predictor" OR "predictors" OR "regression" OR "regressor" OR "regressors”)) OR (KW("machine learning" OR "deep learning" OR "neural network" OR "neural networks" OR "NN" OR "NNs" OR "CNN" OR "CNNs" OR "RNN" OR "RNNs" OR "DNN" OR "DNNs" OR "KNN" OR "k-nearest neighbors" OR "SVM" OR "support vector machine" OR "LSTM" OR "XGBoost" OR "catboost" OR "adaboost" OR "decision tree" OR "decision trees" OR "random forest" OR "random forests" OR "supervised" OR "unsupervised" OR "Naive Bayes" OR "Principal Component Analysis" OR "PCA" OR "dimension reduction" OR "linear discriminate" OR "LDA" OR "transformer" OR "reinforcement learning" OR "computer vision" OR "artificial intelligence" OR "AI" OR "unsupervised learning" OR "supervised learning" OR "data analysis" OR "data analyses" OR "feature extraction" OR "data preprocessing" OR "data processing" OR "signal processing" OR "information processing" OR "feature engineering" OR "statistical model" OR "ANOVA" OR "algorithm" OR "algorithms" OR "classification" OR "classifier" OR "time-series analysis"

OR "time-series analyses" OR "real-time analysis" OR "real-time analyses" OR "sentiment analysis" OR "sentiment analyses" OR "pattern analysis" OR "pattern analyses" OR "prediction" OR "predictor" OR "predictors" OR "regression" OR "regressor" OR "regressors”) OR (MH("machine learning" OR "deep learning" OR "neural network" OR "neural networks" OR "NN" OR "NNs" OR "CNN" OR "CNNs" OR "RNN" OR "RNNs" OR "DNN" OR "DNNs" OR "KNN" OR "k-nearest neighbors" OR "SVM" OR "support vector machine" OR "LSTM" OR "XGBoost" OR "catboost" OR "adaboost" OR "decision tree" OR "decision trees" OR "random forest" OR "random forests" OR "supervised" OR "unsupervised" OR "Naive Bayes" OR "Principal Component Analysis" OR "PCA" OR "dimension reduction" OR "linear discriminate" OR "LDA" OR "transformer" OR "reinforcement learning" OR "computer vision" OR "artificial intelligence" OR "AI" OR "unsupervised learning" OR "supervised learning" OR "data analysis" OR "data analyses" OR "feature extraction" OR "data preprocessing" OR "data processing" OR "signal processing" OR "information processing" OR "feature engineering" OR "statistical model" OR "ANOVA" OR "algorithm" OR "algorithms" OR "classification" OR "classifier" OR "time-series analysis" OR "time-series analyses" OR "real-time analysis" OR "real-time analyses" OR "sentiment analysis" OR "sentiment analyses" OR "pattern analysis" OR "pattern analyses" OR "prediction" OR "predictor" OR "predictors" OR "regression" OR "regressor" OR "regressors”))

S4 S1 AND S2 AND S3

Number of records identified: 989.

Updated search on Dec 4, 2025

Queries:

S1 (XB(accelerome* OR acceleration OR actigraph* OR activPAL OR axivity OR geneactiv OR xsens OR shimmer OR fitbit OR garmin OR polar OR "apple watch" OR smartwatch* OR wearable* OR "activity tracker*" OR "fitness tracker*" OR "fitness band*" OR inertial OR inertia OR IMU OR IMUs OR "IMU-based" OR movesense OR movisens OR "body-worn sensor*" OR "body worn sensor*" OR "motion sensor*" OR "motion tracker*" OR "motion capture") OR (KW(accelerome* OR acceleration OR actigraph* OR activPAL OR axivity OR geneactiv OR xsens OR shimmer OR fitbit OR garmin OR polar OR "apple watch" OR smartwatch* OR wearable* OR "activity tracker*" OR "fitness tracker*" OR "fitness band*" OR inertial OR inertia OR IMU OR IMUs OR "IMU-based" OR movesense OR movisens OR "body-worn sensor*" OR "body worn sensor*" OR "motion sensor*" OR "motion tracker*" OR "motion capture") OR (MH(accelerome* OR acceleration OR actigraph* OR activPAL OR axivity OR geneactiv OR xsens OR shimmer OR fitbit OR garmin OR polar OR "apple watch" OR smartwatch* OR wearable* OR "activity tracker*" OR "fitness tracker*" OR "fitness band*" OR inertial OR inertia OR IMU OR IMUs OR "IMU-based" OR movesense OR movisens OR "body-worn sensor*" OR "body worn sensor*" OR "motion sensor*" OR "motion tracker*" OR "motion capture"))

S2 (XB("prolonged posture" OR "prolonged postures" OR "prolonged sitting" OR "prolonged standing" OR sedentary OR "static posture" OR "static postures" OR "static sitting" OR "static standing" OR "sitting time" OR "standing time" OR "sedentary bout*" OR "sitting bout*" OR "standing bout*" OR "posture bout*" OR "uninterrupted sitting" OR "uninterrupted standing" OR "sustained sitting" OR "sustained standing" OR "sustained posture" OR "bout duration" OR "bout durations" OR "bout length" OR "posture duration" OR

"posture durations" OR "sit-to-stand" OR "sit to stand" OR "stand-to-sit" OR "stand to sit" OR "posture transition" OR "posture change" OR "posture monitoring" OR "posture detection" OR "posture classification" OR "posture recognition" OR "sitting posture" OR "sitting postures" OR "standing posture" OR "standing postures")) OR (KW("prolonged posture" OR "prolonged postures" OR "prolonged sitting" OR "prolonged standing" OR sedentary OR "static posture" OR "static postures" OR "static sitting" OR "static standing" OR "sitting time" OR "standing time" OR "sedentary bout*" OR "sitting bout*" OR "standing bout*" OR "posture bout*" OR "uninterrupted sitting" OR "uninterrupted standing" OR "sustained sitting" OR "sustained standing" OR "sustained posture" OR "bout duration" OR "bout durations" OR "bout length" OR "posture duration" OR "posture durations" OR "sit-to-stand" OR "sit to stand" OR "stand-to-sit" OR "stand to sit" OR "posture transition" OR "posture change" OR "posture monitoring" OR "posture detection" OR "posture classification" OR "posture recognition" OR "sitting posture" OR "sitting postures" OR "standing posture" OR "standing postures")) OR (MH("prolonged posture" OR "prolonged postures" OR "prolonged sitting" OR "prolonged standing" OR sedentary OR "static posture" OR "static postures" OR "static sitting" OR "static standing" OR "sitting time" OR "standing time" OR "sedentary bout*" OR "sitting bout*" OR "standing bout*" OR "posture bout*" OR "uninterrupted sitting" OR "uninterrupted standing" OR "sustained sitting" OR "sustained standing" OR "sustained posture" OR "bout duration" OR "bout durations" OR "bout length" OR "posture duration" OR "posture durations" OR "sit-to-stand" OR "sit to stand" OR "stand-to-sit" OR "stand to sit" OR "posture transition" OR "posture change" OR "posture monitoring" OR "posture detection" OR "posture classification" OR "posture recognition" OR "sitting posture" OR "sitting postures" OR "standing posture" OR "standing postures"))

S3 (XB("artificial intelligence" OR "AI" OR "machine learning" OR "deep learning" OR "supervised learning" OR "unsupervised learning" OR "reinforcement learning" OR "transfer learning" OR "neural network" OR "neural networks" OR "NN" OR "NNs" OR "CNN" OR "CNNs" OR "RNN" OR "RNNs" OR "LSTM" OR "DNN" OR "DNNs" OR "transformer" OR "support vector machine" OR "SVM" OR "decision tree" OR "decision trees" OR "random forest" OR "random forests" OR "XGBoost" OR "CatBoost" OR "AdaBoost" OR "KNN" OR "k-nearest neighbors" OR "Naive Bayes" OR "feature extraction" OR "feature engineering" OR "dimension reduction" OR "Principal Component Analysis" OR "PCA" OR "linear discriminant" OR "LDA" OR "data preprocessing" OR "data processing" OR "signal processing" OR "data analysis" OR "data analyses" OR "classification" OR "classifier" OR "regression" OR "regressor" OR "regressors" OR "prediction" OR "predictor" OR "predictors" OR "pattern analysis" OR "pattern analyses" OR "time-series analysis" OR "time-series analyses" OR "statistical model" OR "statistical learning" OR "threshold" OR "threshold-based" OR "rule-based" OR "real-time analysis" OR "real-time analyses" OR "algorithm" OR "algorithms") OR KW("artificial intelligence" OR "AI" OR "machine learning" OR "deep learning" OR "supervised learning" OR "unsupervised learning" OR "reinforcement learning" OR "transfer learning" OR "neural network" OR "neural networks" OR "NN" OR "NNs" OR "CNN" OR "CNNs" OR "RNN" OR "RNNs" OR "LSTM" OR "DNN" OR "DNNs" OR "transformer" OR "support vector machine" OR "SVM" OR "decision tree" OR "decision trees" OR "random forest" OR "random forests" OR "XGBoost" OR "CatBoost" OR "AdaBoost" OR "KNN" OR "k-nearest neighbors" OR "Naive Bayes" OR "feature extraction" OR "feature engineering" OR "dimension reduction" OR "Principal Component Analysis" OR "PCA" OR "linear discriminant" OR "LDA" OR "data preprocessing" OR "data processing" OR "signal processing" OR "data analysis" OR "data analyses" OR "classification" OR "classifier" OR "regression" OR "regressor" OR "regressors" OR "prediction" OR "predictor" OR "predictors"

OR "pattern analysis" OR "pattern analyses" OR "time-series analysis" OR "time-series analyses" OR "statistical model" OR "statistical learning" OR "threshold" OR "threshold-based" OR "rule-based" OR "real-time analysis" OR "real-time analyses" OR "algorithm" OR "algorithms") OR MH("artificial intelligence" OR "AI" OR "machine learning" OR "deep learning" OR "supervised learning" OR "unsupervised learning" OR "reinforcement learning" OR "transfer learning" OR "neural network" OR "neural networks" OR "NN" OR "NNs" OR "CNN" OR "CNNs" OR "RNN" OR "RNNs" OR "LSTM" OR "DNN" OR "DNNs" OR "transformer" OR "support vector machine" OR "SVM" OR "decision tree" OR "decision trees" OR "random forest" OR "random forests" OR "XGBoost" OR "CatBoost" OR "AdaBoost" OR "KNN" OR "k-nearest neighbors" OR "Naive Bayes" OR "feature extraction" OR "feature engineering" OR "dimension reduction" OR "Principal Component Analysis" OR "PCA" OR "linear discriminant" OR "LDA" OR "data preprocessing" OR "data processing" OR "signal processing" OR "data analysis" OR "data analyses" OR "classification" OR "classifier" OR "regression" OR "regressor" OR "regressors" OR "prediction" OR "predictor" OR "predictors" OR "pattern analysis" OR "pattern analyses" OR "time-series analysis" OR "time-series analyses" OR "statistical model" OR "statistical learning" OR "threshold" OR "threshold-based" OR "rule-based" OR "real-time analysis" OR "real-time analyses" OR "algorithm" OR "algorithms"))

S4 S1 AND S2 AND S3

Number of records identified: 1182.

Catch-up search on Apr 8, 2026

Queries: same as December 4, 2025.

Number of records identified: 1257.

SPORTDiscus

No additional filter applied during database searching.

Initial search on Jan 12, 2025

Queries:

S1 (TI("inertial" OR "inertia" OR "IMU" OR "IMUs" OR "wearable*" OR "actigraph*" OR "accelerome*" OR "acceleration" OR "magnetometer" OR "magnetometers" OR "gyroscope" OR "gyroscopes" OR "activity tracker" OR "activity trackers" OR "fitness tracker" OR "fitness trackers" OR "fitness band" OR "fitness bands" OR "body-worn sensor" OR "body-worn sensors" OR "body worn sensor" OR "body worn sensors" OR "motion sensor" OR "motion sensors" OR "motion tracker" OR "motion trackers" OR "motion capture" OR "smartwatch" OR "smart watch") OR AB("inertial" OR "inertia" OR "IMU" OR "IMUs" OR "wearable*" OR "actigraph*" OR "accelerome*" OR "acceleration" OR "magnetometer" OR "magnetometers" OR "gyroscope" OR "gyroscopes" OR "activity tracker" OR "activity trackers" OR "fitness tracker" OR "fitness trackers" OR "fitness band" OR "fitness bands" OR "body-worn sensor" OR "body-worn sensors" OR "body worn sensor" OR "body worn sensors" OR "motion sensor" OR "motion sensors" OR "motion tracker" OR "motion

trackers" OR "motion capture" OR "smartwatch" OR "smart watch") OR KW("inertial" OR "inertia" OR "IMU" OR "IMUs" OR "wearable*" OR "actigraph*" OR "accelerome*" OR "acceleration" OR "magnetometer" OR "magnetometers" OR "gyroscope" OR "gyroscopes" OR "activity tracker" OR "activity trackers" OR "fitness tracker" OR "fitness trackers" OR "fitness band" OR "fitness bands" OR "body-worn sensor" OR "body-worn sensors" OR "body worn sensor" OR "body worn sensors" OR "motion sensor" OR "motion sensors" OR "motion tracker" OR "motion trackers" OR "motion capture" OR "smartwatch" OR "smart watch"))

S2 TI ("prolonged posture" OR "prolonged postures" OR "sedentary" OR "prolonged sitting" OR "prolonged standing" OR "sitting time" OR "sitting posture" OR "sitting postures" OR "standing time" OR "standing posture" OR "standing postures" OR "static posture" OR "static postures" OR "static sitting" OR "static standing") OR AB ("prolonged posture" OR "prolonged postures" OR "sedentary" OR "prolonged sitting" OR "prolonged standing" OR "sitting time" OR "sitting posture" OR "sitting postures" OR "standing time" OR "standing posture" OR "standing postures" OR "static posture" OR "static postures" OR "static sitting" OR "static standing") OR KW ("prolonged posture" OR "prolonged postures" OR "sedentary" OR "prolonged sitting" OR "prolonged standing" OR "sitting time" OR "sitting posture" OR "sitting postures" OR "standing time" OR "standing posture" OR "standing postures" OR "static posture" OR "static postures" OR "static sitting" OR "static standing")

S3 TI ("machine learning" OR "deep learning" OR "neural network" OR "neural networks" OR "NN" OR "NNs" OR "CNN" OR "CNNs" OR "RNN" OR "RNNs" OR "DNN" OR "DNNs" OR "KNN" OR "k-nearest neighbors" OR "SVM" OR "support vector machine" OR "LSTM" OR "XGBoost" OR "catboost" OR "adaboost" OR "decision tree" OR "decision trees" OR "random forest" OR "random forests" OR "supervised" OR "unsupervised" OR "Naive Bayes" OR " Principal Component Analysis" OR "PCA" OR "dimension reduction" OR "linear discriminate" OR "LDA" OR "transformer" OR "reinforcement learning" OR "computer vision" OR "artificial intelligence" OR "AI" OR "unsupervised learning" OR "supervised learning" OR "data analysis" OR "data analyses" OR "feature extraction" OR "data preprocessing" OR "data processing" OR "signal processing" OR "information processing" OR "feature engineering" OR "statistical model" OR "ANOVA" OR "algorithm" OR "algorithms" OR "classification" OR "classifier" OR "time-series analysis" OR "time-series analyses" OR "real-time analysis" OR "real-time analyses" OR "sentiment analysis" OR "sentiment analyses" OR "pattern analysis" OR "pattern analyses" OR "prediction" OR "predictor" OR "predictors" OR "regression" OR "regressor" OR "regressors") OR AB ("machine learning" OR "deep learning" OR "neural network" OR "neural networks" OR "NN" OR "NNs" OR "CNN" OR "CNNs" OR "RNN" OR "RNNs" OR "DNN" OR "DNNs" OR "KNN" OR "k-nearest neighbors" OR "SVM" OR "support vector machine" OR "LSTM" OR "XGBoost" OR "catboost" OR "adaboost" OR "decision tree" OR "decision trees" OR "random forest" OR "random forests" OR "supervised" OR "unsupervised" OR "Naive Bayes" OR " Principal Component Analysis" OR "PCA" OR "dimension reduction" OR "linear discriminate" OR "LDA" OR "transformer" OR "reinforcement learning" OR "computer vision" OR "artificial intelligence" OR "AI" OR "unsupervised learning" OR "supervised learning" OR "data analysis" OR "data analyses" OR "feature extraction" OR "data preprocessing" OR "data processing" OR "signal processing" OR "information processing" OR "feature engineering" OR "statistical model" OR "ANOVA" OR "algorithm" OR "algorithms" OR "classification" OR "classifier" OR "time-series analysis" OR "time-series analyses" OR "real-time analysis" OR "real-time analyses" OR "sentiment analysis" OR "sentiment analyses" OR "pattern analysis" OR "pattern analyses" OR "prediction" OR "predictor" OR "predictors" OR "regression" OR "regressor" OR "regressors")

OR KW ("machine learning" OR "deep learning" OR "neural network" OR "neural networks" OR "NN" OR "NNs" OR "CNN" OR "CNNs" OR "RNN" OR "RNNs" OR "DNN" OR "DNNs" OR "KNN" OR "k-nearest neighbors" OR "SVM" OR "support vector machine" OR "LSTM" OR "XGBoost" OR "catboost" OR "adaboost" OR "decision tree" OR "decision trees" OR "random forest" OR "random forests" OR "supervised" OR "unsupervised" OR "Naive Bayes" OR " Principal Component Analysis" OR "PCA" OR "dimension reduction" OR "linear discriminate" OR "LDA" OR "transformer" OR "reinforcement learning" OR "computer vision" OR "artificial intelligence" OR "AI" OR "unsupervised learning" OR "supervised learning" OR "data analysis" OR "data analyses" OR "feature extraction" OR "data preprocessing" OR "data processing" OR "signal processing" OR "information processing" OR "feature engineering" OR "statistical model" OR "ANOVA" OR "algorithm" OR "algorithms" OR "classification" OR "classifier" OR "time-series analysis" OR "time-series analyses" OR "real-time analysis" OR "real-time analyses" OR "sentiment analysis" OR "sentiment analyses" OR "pattern analysis" OR "pattern analyses" OR "prediction" OR "predictor" OR "predictors" OR "regression" OR "regressor" OR "regressors")

S4 S1 AND S2 AND S3

Number of records identified: 692.

Updated search on Dec 4, 2025

Queries:

S1 TI ("accelerome*" OR "acceleration" OR "actigraph*" OR "activPAL" OR "Axivity" OR "GENEActiv" OR "Xsens" OR "Shimmer" OR "Fitbit" OR "Garmin" OR "Polar" OR "Apple Watch" OR "wearable*" OR "smartwatch" OR "smart watch" OR "activity tracker" OR "activity trackers" OR "fitness tracker" OR "fitness trackers" OR "fitness band" OR "fitness bands" OR "inertial" OR "inertia" OR "IMU" OR "IMUs" OR "IMU-based" OR "Movesense" OR "movisens" OR "body-worn sensor" OR "body-worn sensors" OR "body worn sensor" OR "body worn sensors" OR "motion sensor" OR "motion sensors" OR "motion tracker" OR "motion trackers" OR "motion capture") OR AB ("accelerome*" OR "acceleration" OR "actigraph*" OR "activPAL" OR "Axivity" OR "GENEActiv" OR "Xsens" OR "Shimmer" OR "Fitbit" OR "Garmin" OR "Polar" OR "Apple Watch" OR "wearable*" OR "smartwatch" OR "smart watch" OR "activity tracker" OR "activity trackers" OR "fitness tracker" OR "fitness trackers" OR "fitness band" OR "fitness bands" OR "inertial" OR "inertia" OR "IMU" OR "IMUs" OR "IMU-based" OR "Movesense" OR "movisens" OR "body-worn sensor" OR "body-worn sensors" OR "body worn sensor" OR "body worn sensors" OR "motion sensor" OR "motion sensors" OR "motion tracker" OR "motion trackers" OR "motion capture") OR KW ("accelerome*" OR "acceleration" OR "actigraph*" OR "activPAL" OR "Axivity" OR "GENEActiv" OR "Xsens" OR "Shimmer" OR "Fitbit" OR "Garmin" OR "Polar" OR "Apple Watch" OR "wearable*" OR "smartwatch" OR "smart watch" OR "activity tracker" OR "activity trackers" OR "fitness tracker" OR "fitness trackers" OR "fitness band" OR "fitness bands" OR "inertial" OR "inertia" OR "IMU" OR "IMUs" OR "IMU-based" OR "Movesense" OR "movisens" OR "body-worn sensor" OR "body-worn sensors" OR "body worn sensor" OR "body worn sensors" OR "motion sensor" OR "motion sensors" OR "motion tracker" OR "motion trackers" OR "motion capture")

S2 TI ("prolonged posture" OR "prolonged postures" OR "sedentary" OR "prolonged sitting" OR "prolonged standing" OR "static posture" OR "static postures" OR "static sitting" OR "static

standing" OR "sitting time" OR "standing time" OR "sedentary bout" OR "sedentary bouts" OR "sitting bout" OR "sitting bouts" OR "standing bout" OR "standing bouts" OR "posture bout" OR "posture bouts" OR "uninterrupted sitting" OR "uninterrupted standing" OR "sustained sitting" OR "sustained standing" OR "sustained posture" OR "bout duration" OR "bout durations" OR "bout length" OR "posture duration" OR "posture durations" OR "sit-to-stand" OR "sit to stand" OR "stand-to-sit" OR "stand to sit" OR "posture transition" OR "posture change" OR "posture monitoring" OR "posture detection" OR "posture classification" OR "posture recognition" OR "sitting posture" OR "sitting postures" OR "standing posture" OR "standing postures") OR AB ("prolonged posture" OR "prolonged postures" OR "sedentary" OR "prolonged sitting" OR "prolonged standing" OR "static posture" OR "static postures" OR "static sitting" OR "static standing" OR "sitting time" OR "standing time" OR "sedentary bout" OR "sedentary bouts" OR "sitting bout" OR "sitting bouts" OR "standing bout" OR "standing bouts" OR "posture bout" OR "posture bouts" OR "uninterrupted sitting" OR "uninterrupted standing" OR "sustained sitting" OR "sustained standing" OR "sustained posture" OR "bout duration" OR "bout durations" OR "bout length" OR "posture duration" OR "posture durations" OR "sit-to-stand" OR "sit to stand" OR "stand-to-sit" OR "stand to sit" OR "posture transition" OR "posture change" OR "posture monitoring" OR "posture detection" OR "posture classification" OR "posture recognition" OR "sitting posture" OR "sitting postures" OR "standing posture" OR "standing postures") OR KW ("prolonged posture" OR "prolonged postures" OR "sedentary" OR "prolonged sitting" OR "prolonged standing" OR "static posture" OR "static postures" OR "static sitting" OR "static standing" OR "sitting time" OR "standing time" OR "sedentary bout" OR "sedentary bouts" OR "sitting bout" OR "sitting bouts" OR "standing bout" OR "standing bouts" OR "posture bout" OR "posture bouts" OR "uninterrupted sitting" OR "uninterrupted standing" OR "sustained sitting" OR "sustained standing" OR "sustained posture" OR "bout duration" OR "bout durations" OR "bout length" OR "posture duration" OR "posture durations" OR "sit-to-stand" OR "sit to stand" OR "stand-to-sit" OR "stand to sit" OR "posture transition" OR "posture change" OR "posture monitoring" OR "posture detection" OR "posture classification" OR "posture recognition" OR "sitting posture" OR "sitting postures" OR "standing posture" OR "standing postures")

S3 TI ("artificial intelligence" OR "AI" OR "machine learning" OR "deep learning" OR "supervised learning" OR "unsupervised learning" OR "reinforcement learning" OR "transfer learning" OR "neural network" OR "neural networks" OR "NN" OR "NNs" OR "CNN" OR "CNNs" OR "RNN" OR "RNNs" OR "LSTM" OR "DNN" OR "DNNs" OR "transformer" OR "support vector machine" OR "SVM" OR "decision tree" OR "decision trees" OR "random forest" OR "random forests" OR "XGBoost" OR "CatBoost" OR "AdaBoost" OR "KNN" OR "k-nearest neighbors" OR "Naive Bayes" OR "feature extraction" OR "feature engineering" OR "dimension reduction" OR "Principal Component Analysis" OR "PCA" OR "linear discriminant" OR "LDA" OR "data preprocessing" OR "data processing" OR "signal processing" OR "data analysis" OR "data analyses" OR "classification" OR "classifier" OR "regression" OR "regressor" OR "regressors" OR "prediction" OR "predictor" OR "predictors" OR "pattern analysis" OR "pattern analyses" OR "time-series analysis" OR "time-series analyses" OR "statistical model" OR "statistical learning" OR "threshold" OR "threshold-based" OR "rule-based" OR "real-time analysis" OR "real-time analyses" OR "algorithm" OR "algorithms") OR AB ("artificial intelligence" OR "AI" OR "machine learning" OR "deep learning" OR "supervised learning" OR "unsupervised learning" OR "reinforcement learning" OR "transfer learning" OR "neural network" OR "neural networks" OR "NN" OR "NNs" OR "CNN" OR "CNNs" OR "RNN" OR "RNNs" OR "LSTM" OR "DNN" OR "DNNs" OR "transformer" OR "support vector

machine" OR "SVM" OR "decision tree" OR "decision trees" OR "random forest" OR "random forests" OR "XGBoost" OR "CatBoost" OR "AdaBoost" OR "KNN" OR "k-nearest neighbors" OR "Naive Bayes" OR "feature extraction" OR "feature engineering" OR "dimension reduction" OR "Principal Component Analysis" OR "PCA" OR "linear discriminant" OR "LDA" OR "data preprocessing" OR "data processing" OR "signal processing" OR "data analysis" OR "data analyses" OR "classification" OR "classifier" OR "regression" OR "regressor" OR "regressors" OR "prediction" OR "predictor" OR "predictors" OR "pattern analysis" OR "pattern analyses" OR "time-series analysis" OR "time-series analyses" OR "statistical model" OR "statistical learning" OR "threshold" OR "threshold-based" OR "rule-based" OR "real-time analysis" OR "real-time analyses" OR "algorithm" OR "algorithms") OR KW ("artificial intelligence" OR "AI" OR "machine learning" OR "deep learning" OR "supervised learning" OR "unsupervised learning" OR "reinforcement learning" OR "transfer learning" OR "neural network" OR "neural networks" OR "NN" OR "NNs" OR "CNN" OR "CNNs" OR "RNN" OR "RNNs" OR "LSTM" OR "DNN" OR "DNNs" OR "transformer" OR "support vector machine" OR "SVM" OR "decision tree" OR "decision trees" OR "random forest" OR "random forests" OR "XGBoost" OR "CatBoost" OR "AdaBoost" OR "KNN" OR "k-nearest neighbors" OR "Naive Bayes" OR "feature extraction" OR "feature engineering" OR "dimension reduction" OR "Principal Component Analysis" OR "PCA" OR "linear discriminant" OR "LDA" OR "data preprocessing" OR "data processing" OR "signal processing" OR "data analysis" OR "data analyses" OR "classification" OR "classifier" OR "regression" OR "regressor" OR "regressors" OR "prediction" OR "predictor" OR "predictors" OR "pattern analysis" OR "pattern analyses" OR "time-series analysis" OR "time-series analyses" OR "statistical model" OR "statistical learning" OR "threshold" OR "threshold-based" OR "rule-based" OR "real-time analysis" OR "real-time analyses" OR "algorithm" OR "algorithms")

S4 ((S1) AND (S2) AND (S3))

Number of records identified: 763.

Catch-up search on Apr 8, 2026

Queries: same as December 4, 2025.

Number of records identified: 815.

IEEE Xplore

No additional filter applied during database searching.

Initial search on Jan 12, 2025

Queries: (("inertial" OR "inertia" OR "IMU" OR "IMUs" OR "wearable*" OR "actigraph*" OR "accelerome*" OR "acceleration" OR "magnetometer" OR "magnetometers" OR "gyroscope" OR "gyroscopes" OR "activity tracker" OR "activity trackers" OR "fitness tracker" OR "fitness trackers" OR "fitness band" OR "fitness bands" OR "body-worn sensor" OR "body-worn sensors" OR "body worn sensor" OR "body worn sensors" OR "motion sensor" OR "motion sensors" OR "motion tracker" OR "motion trackers" OR "motion capture" OR "smartwatch" OR "smart watch") AND ("prolonged posture" OR "prolonged postures" OR "sedentary" OR "prolonged sitting" OR "prolonged standing" OR "sitting time" OR "sitting posture" OR "sitting postures" OR "standing time" OR "standing posture" OR "standing postures" OR "static posture" OR "static postures" OR "static sitting" OR "static standing") AND ("machine learning" OR "deep learning"

OR "neural network" OR "neural networks" OR "NN" OR "NNs" OR "CNN" OR "CNNs" OR "RNN" OR "RNNs" OR "DNN" OR "DNNs" OR "KNN" OR "k-nearest neighbors" OR "SVM" OR "support vector machine" OR "LSTM" OR "XGBoost" OR "catboost" OR "adaboost" OR "decision tree" OR "decision trees" OR "random forest" OR "random forests" OR "supervised" OR "unsupervised" OR "Naive Bayes" OR " Principal Component Analysis" OR "PCA" OR "dimension reduction" OR "linear discriminate" OR "LDA" OR "transformer" OR "reinforcement learning" OR "computer vision" OR "artificial intelligence" OR "AI" OR "unsupervised learning" OR "supervised learning" OR "data analysis" OR "data analyses" OR "feature extraction" OR "data preprocessing" OR "data processing" OR "signal processing" OR "information processing" OR "feature engineering" OR "statistical model" OR "ANOVA" OR "algorithm" OR "algorithms" OR "classification" OR "classifier" OR "time-series analysis" OR "time-series analyses" OR "real-time analysis" OR "real-time analyses" OR "sentiment analysis" OR "sentiment analyses" OR "pattern analysis" OR "pattern analyses" OR "prediction" OR "predictor" OR "predictors" OR "regression" OR "regressor" OR "regressors"))

Number of records identified: 679.

Updated search on Dec 4, 2025

Queries: (("accelerome*" OR "acceleration" OR "actigraph*" OR "activPAL" OR "Axivity" OR "GENEActiv" OR "Xsens" OR "Shimmer" OR "Fitbit" OR "Garmin" OR "Polar" OR "Apple Watch" OR "wearable*" OR "smartwatch" OR "smart watch" OR "activity tracker" OR "activity trackers" OR "fitness tracker" OR "fitness trackers" OR "fitness band" OR "fitness bands" OR "inertial" OR "inertia" OR "IMU" OR "IMUs" OR "IMU-based" OR "Movesense" OR "movisens" OR "body-worn sensor" OR "body-worn sensors" OR "body worn sensor" OR "body worn sensors" OR "motion sensor" OR "motion sensors" OR "motion tracker" OR "motion trackers" OR "motion capture") AND ("prolonged posture" OR "prolonged postures" OR "sedentary" OR "prolonged sitting" OR "prolonged standing" OR "static posture" OR "static postures" OR "static sitting" OR "static standing" OR "sitting time" OR "standing time" OR "sedentary bout" OR "sedentary bouts" OR "sitting bout" OR "sitting bouts" OR "standing bout" OR "standing bouts" OR "posture bout" OR "posture bouts" OR "uninterrupted sitting" OR "uninterrupted standing" OR "sustained sitting" OR "sustained standing" OR "sustained posture" OR "bout duration" OR "bout durations" OR "bout length" OR "posture duration" OR "posture durations" OR "sit-to-stand" OR "sit to stand" OR "stand-to-sit" OR "stand to sit" OR "posture transition" OR "posture change" OR "posture monitoring" OR "posture detection" OR "posture classification" OR "posture recognition" OR "sitting posture" OR "sitting postures" OR "standing posture" OR "standing postures") AND ("artificial intelligence" OR "AI" OR "machine learning" OR "deep learning" OR "supervised learning" OR "unsupervised learning" OR "reinforcement learning" OR "transfer learning" OR "neural network" OR "neural networks" OR "NN" OR "NNs" OR "CNN" OR "CNNs" OR "RNN" OR "RNNs" OR "LSTM" OR "DNN" OR "DNNs" OR "transformer" OR "support vector machine" OR "SVM" OR "decision tree" OR "decision trees" OR "random forest" OR "random forests" OR "XGBoost" OR "CatBoost" OR "AdaBoost" OR "KNN" OR "k-nearest neighbors" OR "Naive Bayes" OR "feature extraction" OR "feature engineering" OR "dimension reduction" OR "Principal Component Analysis" OR "PCA" OR "linear discriminant" OR "LDA" OR "data preprocessing" OR "data processing" OR "signal processing" OR "data analysis" OR "data analyses" OR "classification" OR "classifier" OR "regression" OR "regressor" OR "regressors" OR "prediction" OR "predictor" OR "predictors" OR "pattern analysis" OR "pattern analyses" OR "time-series analysis" OR "time-series analyses" OR "statistical model" OR "statistical learning" OR

"threshold" OR "threshold-based" OR "rule-based" OR "real-time analysis" OR "real-time analyses" OR "algorithm" OR "algorithms"))

Number of records identified: 805.

Catch-up search on Apr 8, 2026

Queries: same as December 4, 2025.

Number of records identified: 1187.

ACM Digital Library

No additional filter applied during database searching.

Initial search on Jan 12, 2025

Queries: Abstract:(("inertial" OR "inertia" OR "IMU" OR "IMUs" OR "wearable*" OR "actigraph*" OR "accelerome*" OR "acceleration" OR "magnetometer" OR "magnetometers" OR "gyroscope" OR "gyroscopes" OR "activity tracker" OR "activity trackers" OR "fitness tracker" OR "fitness trackers" OR "fitness band" OR "fitness bands" OR "body-worn sensor" OR "body-worn sensors" OR "body worn sensor" OR "body worn sensors" OR "motion sensor" OR "motion sensors" OR "motion tracker" OR "motion trackers" OR "motion capture" OR "smartwatch" OR "smart watch") AND ("prolonged posture" OR "prolonged postures" OR "sedentary" OR "prolonged sitting" OR "prolonged standing" OR "sitting time" OR "sitting posture" OR "sitting postures" OR "standing time" OR "standing posture" OR "standing postures" OR "static posture" OR "static postures" OR "static sitting" OR "static standing") AND ("machine learning" OR "deep learning" OR "neural network" OR "neural networks" OR "NN" OR "NNs" OR "CNN" OR "CNNs" OR "RNN" OR "RNNs" OR "DNN" OR "DNNs" OR "KNN" OR "k-nearest neighbors" OR "SVM" OR "support vector machine" OR "LSTM" OR "XGBoost" OR "catboost" OR "adaboost" OR "decision tree" OR "decision trees" OR "random forest" OR "random forests" OR "supervised" OR "unsupervised" OR "Naive Bayes" OR " Principal Component Analysis" OR "PCA" OR "dimension reduction" OR "linear discriminate" OR "LDA" OR "transformer" OR "reinforcement learning" OR "computer vision" OR "artificial intelligence" OR "AI" OR "unsupervised learning" OR "supervised learning" OR "data analysis" OR "data analyses" OR "feature extraction" OR "data preprocessing" OR "data processing" OR "signal processing" OR "information processing" OR "feature engineering" OR "statistical model" OR "ANOVA" OR "algorithm" OR "algorithms" OR "classification" OR "classifier" OR "time-series analysis" OR "time-series analyses" OR "real-time analysis" OR "real-time analyses" OR "sentiment analysis" OR "sentiment analyses" OR "pattern analysis" OR "pattern analyses" OR "prediction" OR "predictor" OR "predictors" OR "regression" OR "regressor" OR "regressors")) OR Title:(("inertial" OR "inertia" OR "IMU" OR "IMUs" OR "wearable*" OR "actigraph*" OR "accelerome*" OR "acceleration" OR "magnetometer" OR "magnetometers" OR "gyroscope" OR "gyroscopes" OR "activity tracker" OR "activity trackers" OR "fitness tracker" OR "fitness trackers" OR "fitness band" OR "fitness bands" OR "body-worn sensor" OR "body-worn sensors" OR "body worn sensor" OR "body worn sensors" OR "motion sensor" OR "motion sensors" OR "motion tracker" OR "motion trackers" OR "motion capture" OR "smartwatch" OR "smart watch") AND ("prolonged posture" OR "prolonged postures" OR "sedentary" OR "prolonged sitting" OR "prolonged standing" OR "sitting time" OR "sitting

posture" OR "sitting postures" OR "standing time" OR "standing posture" OR "standing postures" OR "static posture" OR "static postures" OR "static sitting" OR "static standing") AND ("machine learning" OR "deep learning" OR "neural network" OR "neural networks" OR "NN" OR "NNs" OR "CNN" OR "CNNs" OR "RNN" OR "RNNs" OR "DNN" OR "DNNs" OR "KNN" OR "k-nearest neighbors" OR "SVM" OR "support vector machine" OR "LSTM" OR "XGBoost" OR "catboost" OR "adaboost" OR "decision tree" OR "decision trees" OR "random forest" OR "random forests" OR "supervised" OR "unsupervised" OR "Naive Bayes" OR " Principal Component Analysis" OR "PCA" OR "dimension reduction" OR "linear discriminate" OR "LDA" OR "transformer" OR "reinforcement learning" OR "computer vision" OR "artificial intelligence" OR "AI" OR "unsupervised learning" OR "supervised learning" OR "data analysis" OR "data analyses" OR "feature extraction" OR "data preprocessing" OR "data processing" OR "signal processing" OR "information processing" OR "feature engineering" OR "statistical model" OR "ANOVA" OR "algorithm" OR "algorithms" OR "classification" OR "classifier" OR "time-series analysis" OR "time-series analyses" OR "real-time analysis" OR "real-time analyses" OR "sentiment analysis" OR "sentiment analyses" OR "pattern analysis" OR "pattern analyses" OR "prediction" OR "predictor" OR "predictors" OR "regression" OR "regressor" OR "regressors")) OR Keywords:(("inertial" OR "inertia" OR "IMU" OR "IMUs" OR "wearable*" OR "actigraph*" OR "accelerome*" OR "acceleration" OR "magnetometer" OR "magnetometers" OR "gyroscope" OR "gyroscopes" OR "activity tracker" OR "activity trackers" OR "fitness tracker" OR "fitness trackers" OR "fitness band" OR "fitness bands" OR "body-worn sensor" OR "body-worn sensors" OR "body worn sensor" OR "body worn sensors" OR "motion sensor" OR "motion sensors" OR "motion tracker" OR "motion trackers" OR "motion capture" OR "smartwatch" OR "smart watch") AND ("prolonged posture" OR "prolonged postures" OR "sedentary" OR "prolonged sitting" OR "prolonged standing" OR "sitting time" OR "sitting posture" OR "sitting postures" OR "standing time" OR "standing posture" OR "standing postures" OR "static posture" OR "static postures" OR "static sitting" OR "static standing") AND ("machine learning" OR "deep learning" OR "neural network" OR "neural networks" OR "NN" OR "NNs" OR "CNN" OR "CNNs" OR "RNN" OR "RNNs" OR "DNN" OR "DNNs" OR "KNN" OR "k-nearest neighbors" OR "SVM" OR "support vector machine" OR "LSTM" OR "XGBoost" OR "catboost" OR "adaboost" OR "decision tree" OR "decision trees" OR "random forest" OR "random forests" OR "supervised" OR "unsupervised" OR "Naive Bayes" OR " Principal Component Analysis" OR "PCA" OR "dimension reduction" OR "linear discriminate" OR "LDA" OR "transformer" OR "reinforcement learning" OR "computer vision" OR "artificial intelligence" OR "AI" OR "unsupervised learning" OR "supervised learning" OR "data analysis" OR "data analyses" OR "feature extraction" OR "data preprocessing" OR "data processing" OR "signal processing" OR "information processing" OR "feature engineering" OR "statistical model" OR "ANOVA" OR "algorithm" OR "algorithms" OR "classification" OR "classifier" OR "time-series analysis" OR "time-series analyses" OR "real-time analysis" OR "real-time analyses" OR "sentiment analysis" OR "sentiment analyses" OR "pattern analysis" OR "pattern analyses" OR "prediction" OR "predictor" OR "predictors" OR "regression" OR "regressor" OR "regressors"))

Number of records identified: 23.

Updated search on Dec 4, 2025

Queries: Abstract:(("accelerome*" OR "acceleration" OR "actigraph*" OR "activPAL" OR "Axivity" OR "GENEActiv" OR "Xsens" OR "Shimmer" OR "Fitbit" OR "Garmin" OR "Polar" OR "Apple Watch" OR "wearable*" OR "smartwatch" OR "smart watch" OR "activity tracker" OR

"activity trackers" OR "fitness tracker" OR "fitness trackers" OR "fitness band" OR "fitness bands" OR "inertial" OR "inertia" OR "IMU" OR "IMUs" OR "IMU-based" OR "Movesense" OR "movisens" OR "body-worn sensor" OR "body-worn sensors" OR "body worn sensor" OR "body worn sensors" OR "motion sensor" OR "motion sensors" OR "motion tracker" OR "motion trackers" OR "motion capture") AND ("prolonged posture" OR "prolonged postures" OR "sedentary" OR "prolonged sitting" OR "prolonged standing" OR "static posture" OR "static postures" OR "static sitting" OR "static standing" OR "sitting time" OR "standing time" OR "sedentary bout" OR "sedentary bouts" OR "sitting bout" OR "sitting bouts" OR "standing bout" OR "standing bouts" OR "posture bout" OR "posture bouts" OR "uninterrupted sitting" OR "uninterrupted standing" OR "sustained sitting" OR "sustained standing" OR "sustained posture" OR "bout duration" OR "bout durations" OR "bout length" OR "posture duration" OR "posture durations" OR "sit-to-stand" OR "sit to stand" OR "stand-to-sit" OR "stand to sit" OR "posture transition" OR "posture change" OR "posture monitoring" OR "posture detection" OR "posture classification" OR "posture recognition" OR "sitting posture" OR "sitting postures" OR "standing posture" OR "standing postures") AND ("artificial intelligence" OR "AI" OR "machine learning" OR "deep learning" OR "supervised learning" OR "unsupervised learning" OR "reinforcement learning" OR "transfer learning" OR "neural network" OR "neural networks" OR "NN" OR "NNs" OR "CNN" OR "CNNs" OR "RNN" OR "RNNs" OR "LSTM" OR "DNN" OR "DNNs" OR "transformer" OR "support vector machine" OR "SVM" OR "decision tree" OR "decision trees" OR "random forest" OR "random forests" OR "XGBoost" OR "CatBoost" OR "AdaBoost" OR "KNN" OR "k-nearest neighbors" OR "Naive Bayes" OR "feature extraction" OR "feature engineering" OR "dimension reduction" OR "Principal Component Analysis" OR "PCA" OR "linear discriminant" OR "LDA" OR "data preprocessing" OR "data processing" OR "signal processing" OR "data analysis" OR "data analyses" OR "classification" OR "classifier" OR "regression" OR "regressor" OR "regressors" OR "prediction" OR "predictor" OR "predictors" OR "pattern analysis" OR "pattern analyses" OR "time-series analysis" OR "time-series analyses" OR "statistical model" OR "statistical learning" OR "threshold" OR "threshold-based" OR "rule-based" OR "real-time analysis" OR "real-time analyses" OR "algorithm" OR "algorithms"))

OR Title:(("accelerome*" OR "acceleration" OR "actigraph*" OR "activPAL" OR "Axivity" OR "GENEActiv" OR "Xsens" OR "Shimmer" OR "Fitbit" OR "Garmin" OR "Polar" OR "Apple Watch" OR "wearable*" OR "smartwatch" OR "smart watch" OR "activity tracker" OR "activity trackers" OR "fitness tracker" OR "fitness trackers" OR "fitness band" OR "fitness bands" OR "inertial" OR "inertia" OR "IMU" OR "IMUs" OR "IMU-based" OR "Movesense" OR "movisens" OR "body-worn sensor" OR "body-worn sensors" OR "body worn sensor" OR "body worn sensors" OR "motion sensor" OR "motion sensors" OR "motion tracker" OR "motion trackers" OR "motion capture") AND ("prolonged posture" OR "prolonged postures" OR "sedentary" OR "prolonged sitting" OR "prolonged standing" OR "static posture" OR "static postures" OR "static sitting" OR "static standing" OR "sitting time" OR "standing time" OR "sedentary bout" OR "sedentary bouts" OR "sitting bout" OR "sitting bouts" OR "standing bout" OR "standing bouts" OR "posture bout" OR "posture bouts" OR "uninterrupted sitting" OR "uninterrupted standing" OR "sustained sitting" OR "sustained standing" OR "sustained posture" OR "bout duration" OR "bout durations" OR "bout length" OR "posture duration" OR "posture durations" OR "sit-to-stand" OR "sit to stand" OR "stand-to-sit" OR "stand to sit" OR "posture transition" OR "posture change" OR "posture monitoring" OR "posture detection" OR "posture classification" OR "posture recognition" OR "sitting posture" OR "sitting postures" OR "standing posture" OR "standing postures") AND ("artificial intelligence" OR "AI" OR "machine learning" OR "deep learning" OR "supervised learning" OR "unsupervised learning" OR "reinforcement learning" OR "transfer learning" OR

"neural network" OR "neural networks" OR "NN" OR "NNs" OR "CNN" OR "CNNs" OR "RNN" OR "RNNs" OR "LSTM" OR "DNN" OR "DNNs" OR "transformer" OR "support vector machine" OR "SVM" OR "decision tree" OR "decision trees" OR "random forest" OR "random forests" OR "XGBoost" OR "CatBoost" OR "AdaBoost" OR "KNN" OR "k-nearest neighbors" OR "Naive Bayes" OR "feature extraction" OR "feature engineering" OR "dimension reduction" OR "Principal Component Analysis" OR "PCA" OR "linear discriminant" OR "LDA" OR "data preprocessing" OR "data processing" OR "signal processing" OR "data analysis" OR "data analyses" OR "classification" OR "classifier" OR "regression" OR "regressor" OR "regressors" OR "prediction" OR "predictor" OR "predictors" OR "pattern analysis" OR "pattern analyses" OR "time-series analysis" OR "time-series analyses" OR "statistical model" OR "statistical learning" OR "threshold" OR "threshold-based" OR "rule-based" OR "real-time analysis" OR "real-time analyses" OR "algorithm" OR "algorithms"))

OR Keywords:(("accelerome*" OR "acceleration" OR "actigraph*" OR "activPAL" OR "Axivity" OR "GENEActiv" OR "Xsens" OR "Shimmer" OR "Fitbit" OR "Garmin" OR "Polar" OR "Apple Watch" OR "wearable*" OR "smartwatch" OR "smart watch" OR "activity tracker" OR "activity trackers" OR "fitness tracker" OR "fitness trackers" OR "fitness band" OR "fitness bands" OR "inertial" OR "inertia" OR "IMU" OR "IMUs" OR "IMU-based" OR "Movesense" OR "movisens" OR "body-worn sensor" OR "body-worn sensors" OR "body worn sensor" OR "body worn sensors" OR "motion sensor" OR "motion sensors" OR "motion tracker" OR "motion trackers" OR "motion capture") AND ("prolonged posture" OR "prolonged postures" OR "sedentary" OR "prolonged sitting" OR "prolonged standing" OR "static posture" OR "static postures" OR "static sitting" OR "static standing" OR "sitting time" OR "standing time" OR "sedentary bout" OR "sedentary bouts" OR "sitting bout" OR "sitting bouts" OR "standing bout" OR "standing bouts" OR "posture bout" OR "posture bouts" OR "uninterrupted sitting" OR "uninterrupted standing" OR "sustained sitting" OR "sustained standing" OR "sustained posture" OR "bout duration" OR "bout durations" OR "bout length" OR "posture duration" OR "posture durations" OR "sit-to-stand" OR "sit to stand" OR "stand-to-sit" OR "stand to sit" OR "posture transition" OR "posture change" OR "posture monitoring" OR "posture detection" OR "posture classification" OR "posture recognition" OR "sitting posture" OR "sitting postures" OR "standing posture" OR "standing postures") AND ("artificial intelligence" OR "AI" OR "machine learning" OR "deep learning" OR "supervised learning" OR "unsupervised learning" OR "reinforcement learning" OR "transfer learning" OR "neural network" OR "neural networks" OR "NN" OR "NNs" OR "CNN" OR "CNNs" OR "RNN" OR "RNNs" OR "LSTM" OR "DNN" OR "DNNs" OR "transformer" OR "support vector machine" OR "SVM" OR "decision tree" OR "decision trees" OR "random forest" OR "random forests" OR "XGBoost" OR "CatBoost" OR "AdaBoost" OR "KNN" OR "k-nearest neighbors" OR "Naive Bayes" OR "feature extraction" OR "feature engineering" OR "dimension reduction" OR "Principal Component Analysis" OR "PCA" OR "linear discriminant" OR "LDA" OR "data preprocessing" OR "data processing" OR "signal processing" OR "data analysis" OR "data analyses" OR "classification" OR "classifier" OR "regression" OR "regressor" OR "regressors" OR "prediction" OR "predictor" OR "predictors" OR "pattern analysis" OR "pattern analyses" OR "time-series analysis" OR "time-series analyses" OR "statistical model" OR "statistical learning" OR "threshold" OR "threshold-based" OR "rule-based" OR "real-time analysis" OR "real-time analyses" OR "algorithm" OR "algorithms"))

Number of records identified: 38.

Catch-up search on Apr 8, 2026

Queries: (Abstract:("accelerome*" OR "acceleration" OR "actigraph*" OR "activPAL" OR "Axivity" OR "GENEActiv" OR "Xsens" OR "Shimmer" OR "Fitbit" OR "Garmin" OR "Polar" OR "Apple Watch" OR "wearable*" OR "smartwatch" OR "smart watch" OR "activity tracker" OR "activity trackers" OR "fitness tracker" OR "fitness trackers" OR "fitness band" OR "fitness bands" OR "inertial" OR "inertia" OR "IMU" OR "IMUs" OR "IMU-based" OR "Movesense" OR "movisens" OR "body-worn sensor" OR "body-worn sensors" OR "body worn sensor" OR "body worn sensors" OR "motion sensor" OR "motion sensors" OR "motion tracker" OR "motion trackers" OR "motion capture") OR Title:("accelerome*" OR "acceleration" OR "actigraph*" OR "activPAL" OR "Axivity" OR "GENEActiv" OR "Xsens" OR "Shimmer" OR "Fitbit" OR "Garmin" OR "Polar" OR "Apple Watch" OR "wearable*" OR "smartwatch" OR "smart watch" OR "activity tracker" OR "activity trackers" OR "fitness tracker" OR "fitness trackers" OR "fitness band" OR "fitness bands" OR "inertial" OR "inertia" OR "IMU" OR "IMUs" OR "IMU-based" OR "Movesense" OR "movisens" OR "body-worn sensor" OR "body-worn sensors" OR "body worn sensor" OR "body worn sensors" OR "motion sensor" OR "motion sensors" OR "motion tracker" OR "motion trackers" OR "motion capture") OR Keyword:("accelerome*" OR "acceleration" OR "actigraph*" OR "activPAL" OR "Axivity" OR "GENEActiv" OR "Xsens" OR "Shimmer" OR "Fitbit" OR "Garmin" OR "Polar" OR "Apple Watch" OR "wearable*" OR "smartwatch" OR "smart watch" OR "activity tracker" OR "activity trackers" OR "fitness tracker" OR "fitness trackers" OR "fitness band" OR "fitness bands" OR "inertial" OR "inertia" OR "IMU" OR "IMUs" OR "IMU-based" OR "Movesense" OR "movisens" OR "body-worn sensor" OR "body-worn sensors" OR "body worn sensor" OR "body worn sensors" OR "motion sensor" OR "motion sensors" OR "motion tracker" OR "motion trackers" OR "motion capture")) AND (Abstract:("prolonged posture" OR "prolonged postures" OR "sedentary" OR "prolonged sitting" OR "prolonged standing" OR "static posture" OR "static postures" OR "static sitting" OR "static standing" OR "sitting time" OR "standing time" OR "sedentary bout" OR "sedentary bouts" OR "sitting bout" OR "sitting bouts" OR "standing bout" OR "standing bouts" OR "posture bout" OR "posture bouts" OR "uninterrupted sitting" OR "uninterrupted standing" OR "sustained sitting" OR "sustained standing" OR "sustained posture" OR "bout duration" OR "bout durations" OR "bout length" OR "posture duration" OR "posture durations" OR "sit-to-stand" OR "sit to stand" OR "stand-to-sit" OR "stand to sit" OR "posture transition" OR "posture change" OR "posture monitoring" OR "posture detection" OR "posture classification" OR "posture recognition" OR "sitting posture" OR "sitting postures" OR "standing posture" OR "standing postures") OR Title:("prolonged posture" OR "prolonged postures" OR "sedentary" OR "prolonged sitting" OR "prolonged standing" OR "static posture" OR "static postures" OR "static sitting" OR "static standing" OR "sitting time" OR "standing time" OR "sedentary bout" OR "sedentary bouts" OR "sitting bout" OR "sitting bouts" OR "standing bout" OR "standing bouts" OR "posture bout" OR "posture bouts" OR "uninterrupted sitting" OR "uninterrupted standing" OR "sustained sitting" OR "sustained standing" OR "sustained posture" OR "bout duration" OR "bout durations" OR "bout length" OR "posture duration" OR "posture durations" OR "sit-to-stand" OR "sit to stand" OR "stand-to-sit" OR "stand to sit" OR "posture transition" OR "posture change" OR "posture monitoring" OR "posture detection" OR "posture classification" OR "posture recognition" OR "sitting posture" OR "sitting postures" OR "standing posture" OR "standing postures") OR Keyword:("prolonged posture" OR "prolonged postures" OR "sedentary" OR "prolonged sitting" OR "prolonged standing" OR "static posture" OR "static postures" OR "static sitting" OR "static standing" OR "sitting time" OR "standing time" OR "sedentary bout" OR "sedentary bouts" OR "sitting bout" OR "sitting bouts" OR "standing bout" OR "standing bouts" OR "posture bout" OR "posture bouts" OR "uninterrupted sitting" OR

"uninterrupted standing" OR "sustained sitting" OR "sustained standing" OR "sustained posture" OR "bout duration" OR "bout durations" OR "bout length" OR "posture duration" OR "posture durations" OR "sit-to-stand" OR "sit to stand" OR "stand-to-sit" OR "stand to sit" OR "posture transition" OR "posture change" OR "posture monitoring" OR "posture detection" OR "posture classification" OR "posture recognition" OR "sitting posture" OR "sitting postures" OR "standing posture" OR "standing postures")) AND (Abstract:("artificial intelligence" OR "AI" OR "machine learning" OR "deep learning" OR "supervised learning" OR "unsupervised learning" OR "reinforcement learning" OR "transfer learning" OR "neural network" OR "neural networks" OR "NN" OR "NNs" OR "CNN" OR "CNNs" OR "RNN" OR "RNNs" OR "LSTM" OR "DNN" OR "DNNs" OR "transformer" OR "support vector machine" OR "SVM" OR "decision tree" OR "decision trees" OR "random forest" OR "random forests" OR "XGBoost" OR "CatBoost" OR "AdaBoost" OR "KNN" OR "k-nearest neighbors" OR "Naive Bayes" OR "feature extraction" OR "feature engineering" OR "dimension reduction" OR "Principal Component Analysis" OR "PCA" OR "linear discriminant" OR "LDA" OR "data preprocessing" OR "data processing" OR "signal processing" OR "data analysis" OR "data analyses" OR "classification" OR "classifier" OR "regression" OR "regressor" OR "regressors" OR "prediction" OR "predictor" OR "predictors" OR "pattern analysis" OR "pattern analyses" OR "time-series analysis" OR "time-series analyses" OR "statistical model" OR "statistical learning" OR "threshold" OR "threshold-based" OR "rule-based" OR "real-time analysis" OR "real-time analyses" OR "algorithm" OR "algorithms") OR Title:("artificial intelligence" OR "AI" OR "machine learning" OR "deep learning" OR "supervised learning" OR "unsupervised learning" OR "reinforcement learning" OR "transfer learning" OR "neural network" OR "neural networks" OR "NN" OR "NNs" OR "CNN" OR "CNNs" OR "RNN" OR "RNNs" OR "LSTM" OR "DNN" OR "DNNs" OR "transformer" OR "support vector machine" OR "SVM" OR "decision tree" OR "decision trees" OR "random forest" OR "random forests" OR "XGBoost" OR "CatBoost" OR "AdaBoost" OR "KNN" OR "k-nearest neighbors" OR "Naive Bayes" OR "feature extraction" OR "feature engineering" OR "dimension reduction" OR "Principal Component Analysis" OR "PCA" OR "linear discriminant" OR "LDA" OR "data preprocessing" OR "data processing" OR "signal processing" OR "data analysis" OR "data analyses" OR "classification" OR "classifier" OR "regression" OR "regressor" OR "regressors" OR "prediction" OR "predictor" OR "predictors" OR "pattern analysis" OR "pattern analyses" OR "time-series analysis" OR "time-series analyses" OR "statistical model" OR "statistical learning" OR "threshold" OR "threshold-based" OR "rule-based" OR "real-time analysis" OR "real-time analyses" OR "algorithm" OR "algorithms") OR Keyword:("artificial intelligence" OR "AI" OR "machine learning" OR "deep learning" OR "supervised learning" OR "unsupervised learning" OR "reinforcement learning" OR "transfer learning" OR "neural network" OR "neural networks" OR "NN" OR "NNs" OR "CNN" OR "CNNs" OR "RNN" OR "RNNs" OR "LSTM" OR "DNN" OR "DNNs" OR "transformer" OR "support vector machine" OR "SVM" OR "decision tree" OR "decision trees" OR "random forest" OR "random forests" OR "XGBoost" OR "CatBoost" OR "AdaBoost" OR "KNN" OR "k-nearest neighbors" OR "Naive Bayes" OR "feature extraction" OR "feature engineering" OR "dimension reduction" OR "Principal Component Analysis" OR "PCA" OR "linear discriminant" OR "LDA" OR "data preprocessing" OR "data processing" OR "signal processing" OR "data analysis" OR "data analyses" OR "classification" OR "classifier" OR "regression" OR "regressor" OR "regressors" OR "prediction" OR "predictor" OR "predictors" OR "pattern analysis" OR "pattern analyses" OR "time-series analysis" OR "time-series analyses" OR "statistical model" OR "statistical learning" OR "threshold" OR "threshold-based" OR "rule-based" OR "real-time analysis" OR "real-time analyses" OR "algorithm" OR "algorithms"))

Revision of Boolean logic structure:

- Previous structure used on Jan 12, 2025 and Dec 4, 2025:
  Abstract:(concept 1 AND concept 2 AND concept 3)
  OR Title:(concept 1 AND concept 2 AND concept 3)
  OR Keyword:(concept 1 AND concept 2 AND concept 3)
- Revised structure used on Apr 8, 2026:
  (Abstract:(concept 1) OR Title:(concept 1) OR Keyword:(concept 1))
  AND (Abstract:(concept 2) OR Title:(concept 2) OR Keyword:(concept 2))
  AND (Abstract:(concept 3) OR Title:(concept 3) OR Keyword:(concept 3))
- Rationale: The revised structure includes a greater number of results and ensures consistency with the search logic used in other databases.
- The search terms are same as December 4, 2025.

Number of records identified: 59.

EI

No additional filter applied during database searching.

Initial search on Jan 12, 2025

Queries:

#1 (((inertial OR inertia OR imu OR imus OR wearable* OR actigraph* OR accelerome* OR acceleration OR magnetometer OR magnetometers OR gyroscope OR gyroscopes OR "activity tracker" OR "activity trackers" OR "fitness tracker" OR "fitness trackers" OR "fitness band" OR "fitness bands" OR "body-worn sensor" OR "body-worn sensors" OR "body worn sensor" OR "body worn sensors" OR "motion sensor" OR "motion sensors" OR "motion tracker" OR "motion trackers" OR "motion capture" OR smartwatch OR "smart watch") WN TI) OR ((inertial OR inertia OR imu OR imus OR wearable* OR actigraph* OR accelerome* OR acceleration OR magnetometer OR magnetometers OR gyroscope OR gyroscopes OR "activity tracker" OR "activity trackers" OR "fitness tracker" OR "fitness trackers" OR "fitness band" OR "fitness bands" OR "body-worn sensor" OR "body-worn sensors" OR "body worn sensor" OR "body worn sensors" OR "motion sensor" OR "motion sensors" OR "motion tracker" OR "motion trackers" OR "motion capture" OR smartwatch OR "smart watch") WN AB) OR ((inertial OR inertia OR imu OR imus OR wearable* OR actigraph* OR accelerome* OR acceleration OR magnetometer OR magnetometers OR gyroscope OR gyroscopes OR "activity tracker" OR "activity trackers" OR "fitness tracker" OR "fitness trackers" OR "fitness band" OR "fitness bands" OR "body-worn sensor" OR "body-worn sensors" OR "body worn sensor" OR "body worn sensors" OR "motion sensor" OR "motion sensors" OR "motion tracker" OR "motion trackers" OR "motion capture" OR smartwatch OR "smart watch") WN KY))

#2 (((sedentary OR "prolonged posture" OR "prolonged postures" OR "prolonged sitting" OR "prolonged standing" OR "sitting time" OR "sitting posture" OR "sitting postures" OR "standing time" OR "standing posture" OR "standing postures" OR "static posture" OR "static postures" OR "static sitting" OR "static standing") WN TI) OR ((sedentary OR "prolonged posture" OR "prolonged postures" OR "prolonged sitting" OR "prolonged standing" OR "sitting time" OR "sitting posture" OR "sitting postures" OR "standing time" OR "standing posture" OR "standing postures" OR "static posture" OR "static postures" OR "static sitting"

OR "static standing") WN AB) OR ((sedentary OR "prolonged posture" OR "prolonged postures" OR "prolonged sitting" OR "prolonged standing" OR "sitting time" OR "sitting posture" OR "sitting postures" OR "standing time" OR "standing posture" OR "standing postures" OR "static posture" OR "static postures" OR "static sitting" OR "static standing") WN KY))

#3 ((("machine learning" OR "deep learning" OR "neural network" OR "neural networks" OR nn OR nns OR cnn OR cnns OR rnn OR rnns OR dnn OR dnns OR knn OR "k-nearest neighbors" OR svm OR "support vector machine" OR lstm OR xgboost OR catboost OR adaboost OR "decision tree" OR "decision trees" OR "random forest" OR "random forests" OR supervised OR unsupervised OR "naive bayes" OR "principal component analysis" OR pca OR "dimension reduction" OR "linear discriminate" OR lda OR transformer OR "reinforcement learning" OR "computer vision" OR "artificial intelligence" OR ai OR "unsupervised learning" OR "supervised learning" OR "data analysis" OR "data analyses" OR "feature extraction" OR "data preprocessing" OR "data processing" OR "signal processing" OR "information processing" OR "feature engineering" OR "statistical model" OR anova OR algorithm OR algorithms OR classification OR classifier OR "time-series analysis" OR "time-series analyses" OR "real-time analysis" OR "real-time analyses" OR "sentiment analysis" OR "sentiment analyses" OR "pattern analysis" OR "pattern analyses" OR prediction OR predictor OR predictors OR regression OR regressor OR regressors) WN TI) OR (("machine learning" OR "deep learning" OR "neural network" OR "neural networks" OR nn OR nns OR cnn OR cnns OR rnn OR rnns OR dnn OR dnns OR knn OR "k-nearest neighbors" OR svm OR "support vector machine" OR lstm OR xgboost OR catboost OR adaboost OR "decision tree" OR "decision trees" OR "random forest" OR "random forests" OR supervised OR unsupervised OR "naive bayes" OR "principal component analysis" OR pca OR "dimension reduction" OR "linear discriminate" OR lda OR transformer OR "reinforcement learning" OR "computer vision" OR "artificial intelligence" OR ai OR "unsupervised learning" OR "supervised learning" OR "data analysis" OR "data analyses" OR "feature extraction" OR "data preprocessing" OR "data processing" OR "signal processing" OR "information processing" OR "feature engineering" OR "statistical model" OR anova OR algorithm OR algorithms OR classification OR classifier OR "time-series analysis" OR "time-series analyses" OR "real-time analysis" OR "real-time analyses" OR "sentiment analysis" OR "sentiment analyses" OR "pattern analysis" OR "pattern analyses" OR prediction OR predictor OR predictors OR regression OR regressor OR regressors) WN AB) OR (("machine learning" OR "deep learning" OR "neural network" OR "neural networks" OR nn OR nns OR cnn OR cnns OR rnn OR rnns OR dnn OR dnns OR knn OR "k-nearest neighbors" OR svm OR "support vector machine" OR lstm OR xgboost OR catboost OR adaboost OR "decision tree" OR "decision trees" OR "random forest" OR "random forests" OR supervised OR unsupervised OR "naive bayes" OR "principal component analysis" OR pca OR "dimension reduction" OR "linear discriminate" OR lda OR transformer OR "reinforcement learning" OR "computer vision" OR "artificial intelligence" OR ai OR "unsupervised learning" OR "supervised learning" OR "data analysis" OR "data analyses" OR "feature extraction" OR "data preprocessing" OR "data processing" OR "signal processing" OR "information processing" OR "feature engineering" OR "statistical model" OR anova OR algorithm OR algorithms OR classification OR classifier OR "time-series analysis" OR "time-series analyses" OR "real-time analysis" OR "real-time analyses" OR "sentiment analysis" OR "sentiment analyses" OR "pattern analysis" OR "pattern analyses" OR prediction OR predictor OR predictors OR regression OR regressor OR regressors) WN KY))

#4 #1 AND #2 AND #3

Number of records identified: 438.

Catch-up search on Apr 8, 2026

#1 (("accelerome*" OR "acceleration" OR "actigraph*" OR "activPAL" OR "Axivity" OR "GENEActiv" OR "Xsens" OR "Shimmer" OR "Fitbit" OR "Garmin" OR "Polar" OR "Apple Watch" OR "wearable*" OR "smartwatch" OR "smart watch" OR "activity tracker" OR "activity trackers" OR "fitness tracker" OR "fitness trackers" OR "fitness band" OR "fitness bands" OR "inertial" OR "inertia" OR "IMU" OR "IMUs" OR "IMU-based" OR "Movesense" OR "movisens" OR "body-worn sensor" OR "body-worn sensors" OR "body worn sensor" OR "body worn sensors" OR "motion sensor" OR "motion sensors" OR "motion tracker" OR "motion trackers" OR "motion capture") WN TI) OR (("accelerome*" OR "acceleration" OR "actigraph*" OR "activPAL" OR "Axivity" OR "GENEActiv" OR "Xsens" OR "Shimmer" OR "Fitbit" OR "Garmin" OR "Polar" OR "Apple Watch" OR "wearable*" OR "smartwatch" OR "smart watch" OR "activity tracker" OR "activity trackers" OR "fitness tracker" OR "fitness trackers" OR "fitness band" OR "fitness bands" OR "inertial" OR "inertia" OR "IMU" OR "IMUs" OR "IMU-based" OR "Movesense" OR "movisens" OR "body-worn sensor" OR "body-worn sensors" OR "body worn sensor" OR "body worn sensors" OR "motion sensor" OR "motion sensors" OR "motion tracker" OR "motion trackers" OR "motion capture") WN AB) OR (("accelerome*" OR "acceleration" OR "actigraph*" OR "activPAL" OR "Axivity" OR "GENEActiv" OR "Xsens" OR "Shimmer" OR "Fitbit" OR "Garmin" OR "Polar" OR "Apple Watch" OR "wearable*" OR "smartwatch" OR "smart watch" OR "activity tracker" OR "activity trackers" OR "fitness tracker" OR "fitness trackers" OR "fitness band" OR "fitness bands" OR "inertial" OR "inertia" OR "IMU" OR "IMUs" OR "IMU-based" OR "Movesense" OR "movisens" OR "body-worn sensor" OR "body-worn sensors" OR "body worn sensor" OR "body worn sensors" OR "motion sensor" OR "motion sensors" OR "motion tracker" OR "motion trackers" OR "motion capture") WN KY)

#2 ((("prolonged posture" OR "prolonged postures" OR "sedentary" OR "prolonged sitting" OR "prolonged standing" OR "static posture" OR "static postures" OR "static sitting" OR "static standing" OR "sitting time" OR "standing time" OR "sedentary bout" OR "sedentary bouts" OR "sitting bout" OR "sitting bouts" OR "standing bout" OR "standing bouts" OR "posture bout" OR "posture bouts" OR "uninterrupted sitting" OR "uninterrupted standing" OR "sustained sitting" OR "sustained standing" OR "sustained posture" OR "bout duration" OR "bout durations" OR "bout length" OR "posture duration" OR "posture durations" OR "sit-to-stand" OR "sit to stand" OR "stand-to-sit" OR "stand to sit" OR "posture transition" OR "posture change" OR "posture monitoring" OR "posture detection" OR "posture classification" OR "posture recognition" OR "sitting posture" OR "sitting postures" OR "standing posture" OR "standing postures") WN TI) OR (("prolonged posture" OR "prolonged postures" OR "sedentary" OR "prolonged sitting" OR "prolonged standing" OR "static posture" OR "static postures" OR "static sitting" OR "static standing" OR "sitting time" OR "standing time" OR "sedentary bout" OR "sedentary bouts" OR "sitting bout" OR "sitting bouts" OR "standing bout" OR "standing bouts" OR "posture bout" OR "posture bouts" OR "uninterrupted sitting" OR "uninterrupted standing" OR "sustained sitting" OR "sustained standing" OR "sustained posture" OR "bout duration" OR "bout durations" OR "bout length" OR "posture duration" OR "posture durations" OR "sit-to-stand" OR "sit to stand" OR "stand-to-sit" OR "stand to sit" OR "posture transition" OR "posture change" OR "posture monitoring" OR "posture detection" OR "posture classification" OR "posture recognition" OR

"sitting posture" OR "sitting postures" OR "standing posture" OR "standing postures") WN AB) OR (("prolonged posture" OR "prolonged postures" OR "sedentary" OR "prolonged sitting" OR "prolonged standing" OR "static posture" OR "static postures" OR "static sitting" OR "static standing" OR "sitting time" OR "standing time" OR "sedentary bout" OR "sedentary bouts" OR "sitting bout" OR "sitting bouts" OR "standing bout" OR "standing bouts" OR "posture bout" OR "posture bouts" OR "uninterrupted sitting" OR "uninterrupted standing" OR "sustained sitting" OR "sustained standing" OR "sustained posture" OR "bout duration" OR "bout durations" OR "bout length" OR "posture duration" OR "posture durations" OR "sit-to-stand" OR "sit to stand" OR "stand-to-sit" OR "stand to sit" OR "posture transition" OR "posture change" OR "posture monitoring" OR "posture detection" OR "posture classification" OR "posture recognition" OR "sitting posture" OR "sitting postures" OR "standing posture" OR "standing postures") WN KY))

#3 ((("artificial intelligence" OR "AI" OR "machine learning" OR "deep learning" OR "supervised learning" OR "unsupervised learning" OR "reinforcement learning" OR "transfer learning" OR "neural network" OR "neural networks" OR "NN" OR "NNs" OR "CNN" OR "CNNs" OR "RNN" OR "RNNs" OR "LSTM" OR "DNN" OR "DNNs" OR "transformer" OR "support vector machine" OR "SVM" OR "decision tree" OR "decision trees" OR "random forest" OR "random forests" OR "XGBoost" OR "CatBoost" OR "AdaBoost" OR "KNN" OR "k-nearest neighbors" OR "Naive Bayes" OR "feature extraction" OR "feature engineering" OR "dimension reduction" OR "Principal Component Analysis" OR "PCA" OR "linear discriminant" OR "LDA" OR "data preprocessing" OR "data processing" OR "signal processing" OR "data analysis" OR "data analyses" OR "classification" OR "classifier" OR "regression" OR "regressor" OR "regressors" OR "prediction" OR "predictor" OR "predictors" OR "pattern analysis" OR "pattern analyses" OR "time-series analysis" OR "time-series analyses" OR "statistical model" OR "statistical learning" OR "threshold" OR "threshold-based" OR "rule-based" OR "real-time analysis" OR "real-time analyses" OR "algorithm" OR "algorithms") WN TI) OR (("artificial intelligence" OR "AI" OR "machine learning" OR "deep learning" OR "supervised learning" OR "unsupervised learning" OR "reinforcement learning" OR "transfer learning" OR "neural network" OR "neural networks" OR "NN" OR "NNs" OR "CNN" OR "CNNs" OR "RNN" OR "RNNs" OR "LSTM" OR "DNN" OR "DNNs" OR "transformer" OR "support vector machine" OR "SVM" OR "decision tree" OR "decision trees" OR "random forest" OR "random forests" OR "XGBoost" OR "CatBoost" OR "AdaBoost" OR "KNN" OR "k-nearest neighbors" OR "Naive Bayes" OR "feature extraction" OR "feature engineering" OR "dimension reduction" OR "Principal Component Analysis" OR "PCA" OR "linear discriminant" OR "LDA" OR "data preprocessing" OR "data processing" OR "signal processing" OR "data analysis" OR "data analyses" OR "classification" OR "classifier" OR "regression" OR "regressor" OR "regressors" OR "prediction" OR "predictor" OR "predictors" OR "pattern analysis" OR "pattern analyses" OR "time-series analysis" OR "time-series analyses" OR "statistical model" OR "statistical learning" OR "threshold" OR "threshold-based" OR "rule-based" OR "real-time analysis" OR "real-time analyses" OR "algorithm" OR "algorithms") WN AB) OR (("artificial intelligence" OR "AI" OR "machine learning" OR "deep learning" OR "supervised learning" OR "unsupervised learning" OR "reinforcement learning" OR "transfer learning" OR "neural network" OR "neural networks" OR "NN" OR "NNs" OR "CNN" OR "CNNs" OR "RNN" OR "RNNs" OR "LSTM" OR "DNN" OR "DNNs" OR "transformer" OR "support vector machine" OR "SVM" OR "decision tree" OR "decision trees" OR "random forest" OR "random forests" OR "XGBoost" OR "CatBoost" OR "AdaBoost" OR "KNN" OR "k-nearest neighbors" OR "Naive Bayes" OR "feature extraction" OR "feature engineering" OR "dimension reduction" OR "Principal Component Analysis" OR "PCA" OR "linear discriminant" OR "LDA"

OR "data preprocessing" OR "data processing" OR "signal processing" OR "data analysis" OR "data analyses" OR "classification" OR "classifier" OR "regression" OR "regressor" OR "regressors" OR "prediction" OR "predictor" OR "predictors" OR "pattern analysis" OR "pattern analyses" OR "time-series analysis" OR "time-series analyses" OR "statistical model" OR "statistical learning" OR "threshold" OR "threshold-based" OR "rule-based" OR "real-time analysis" OR "real-time analyses" OR "algorithm" OR "algorithms") WN KY))

#4 #1 AND #2 AND #3

Number of records identified: 1372.
