# Supplementary material for: A systematic review of prolonged posture monitoring with accelerometer-based wearable sensors
Source: PLOS Digit Health. 2026 Sep 15;5(9):e0001538. doi: 10.1371/journal.pdig.0001538 (PMC13577576; doi:10.1371/journal.pdig.0001538)
Supplement: Supplementary file 4 [file pdig.0001538.s006.pdf]

Category	Sub-category	First author and reference number	O'Dolan et al. [31]	Tjurin et al. [32]	Bellettiere et al. [33]	Carlson et al. [13]	Kuster et al. [9]	Colomer et al. [34]	Lögdal et al. [14]	Renaud et al. [27]	Brakenridge et al. [35]	Hamer et al. [36]	Kerr et al. [37]	Evans et al. [38]	Hartikainen et al. [39]	Greenwood-Hickman et al. [15]	Count	Percentage
**Prolonged posture characteristics**	Monitored prolonged posture	Sitting	X	X	X	X	X	X	X	X	X	X	X	X	X	X	14	100%
		Standing															0	0%
	Prolonged sitting/standing bout threshold	5 min					X										1	7.1%
		10 min					X										1	7.1%
		20 min	X										X				2	14.3%
		30 min	X		X	X				X	X		X	X		X	8	57.1%
		60 min								X		X					2	14.3%
		Reported multiple bout durations without defining prolonged bouts		X				X	X						X		4	28.6%
**Data collection**	Measurement environment	Unrestricted free-living settings	X	X	X	X	X	X		X	X	X	X			X	11	78.6%
		Free-living in occupational contexts	X					X	X	X	X			X			6	42.9%
		Free-living in educational contexts													X		1	7.1%
	Number of participants analyzed	< 30	X											X			2	14.3%
		[30, 100)		X			X						X				3	21.4%
		[100, 500)				X		X	X	X	X				X		6	42.9%
		≥ 500			X							X				X	3	21.4%
	Population (health status)	healthy subjects / did not specify	X	X	X	X	X	X	X	X	X	X		X	X	X	13	92.9%
		Patients / frail older adults						X					X			X	3	21.4%
	Population (age group)	Children / adolescents				X									X		2	14.3%
		Adults (18-65)	X	X	X		X	X	X	X	X	X	X	X			11	78.6%
		Older adults (65+)			X								X			X	3	21.4%
	Sensor type	3 axis accelerometer	X	X	X	X	X	X	X	X	X	X	X		X	X	13	92.9%
		Did not specify the model type												X			1	7.1%
	Reference criterion	activPAL			X	X	X	X		X	X	X	X	X		X	10	71.4%
		Self-report diary / log	X		X		X	X	X	X	X	X		X	X	X	11	78.6%
		Other reference							X						X		2	14.3%
		Did not explicitly mention		X													1	7.1%
	Wear protocol duration	< 7 days												X	X		2	14.3%
		≥ 7 days	X	X	X	X	X	X	X	X	X	X	X			X	12	85.7%
	Sampling rates	20 Hz					X			X		X					3	21.4%
		25 Hz							X								1	7.1%
		30 Hz			X	X	X						X			X	5	35.7%
		100 Hz		X											X		2	14.3%
		Not explicitly mentioned	X					X			X			X			4	28.6%
**Data preparation**	Signal extraction and preprocessing	Proprietary software packages / validated processing tools	X		X		X	X	X	X	X	X		X	X	X	11	78.6%
		Other approaches / additional (pre)processing		X		X	X	X	X		X	X	X	X	X	X	11	78.6%
	Wear-time, sleep, and invalid-data handling	Diary-based	X		X		X	X	X	X	X	X		X	X	X	11	78.6%
		Algorithm-based		X	X	X	X			X		X	X			X	8	57.1%
	Segmentation and epoch selection	Event-based segmentation	X					X		X	X	X		X			6	42.9%
		Fixed-window epoches		X	X	X							X		X	X	6	42.9%
		Other methods					X		X								2	14.3%
	Feature extraction	Manually engineered features		X			X						X				3	21.4%
		Outcome-based features	X					X	X	X	X	X		X	X		8	57.1%
		Automatically learned features			X	X										X	3	21.4%
**Data analysis**	Primary analytical approach	Non-machine learning	X					X	X	X	X	X		X	X		8	57.1%
		Machine learning		X	X	X	X						X			X	6	42.9%
	Timing	Real-time modeling															0	0%
		Post-hoc modeling	X	X	X	X	X	X	X	X	X	X	X	X	X	X	14	100%
**User feedback**	User feedback	User feedback	X							X	X			X			4	28.6%
**Reporting items**	Codes availability	Cited the open source libraries / models / tools								X		X					2	14.3%
		Original codes available online			X	X	X									X	4	28.6%
		No statement on codes availability	X	X				X	X		X		X	X	X		8	57.1%
	Dataset availability	Publicially available										X					1	7.1%
		Upon request	X	X		X			X	X					X		6	42.9%
		Restricted									X						1	7.1%
		No statement on dataset availability			X		X	X					X	X		X	6	42.9%
	Protocol / trial registration	Published protocol /trial registration	X					X		X	X			X			5	35.7%
		Not available or applicable		X	X	X	X		X			X	X		X	X	9	64.3%
**Study category**	Study category	Interventional studies	X							X	X			X			4	28.6%
		Method development and validation studies			X	X	X						X			X	5	35.7%
		Observational / feasibility studies		X				X	X			X			X		5	35.7%
		**Year of publication (20XX)**	18	19	22	22	20	22	25	20	16	20	18	12	22	21		
