# Supplementary material for: A systematic review of prolonged posture monitoring with accelerometer-based wearable sensors
Source: PLOS Digit Health. 2026 Sep 15;5(9):e0001538. doi: 10.1371/journal.pdig.0001538 (PMC13577576; doi:10.1371/journal.pdig.0001538)
Supplement: Supplementary file 5 [file pdig.0001538.s008.pdf]

a) Overall impression

This manuscript presents a systematic review of accelerometer-based wearable approaches for *prolonged posture monitoring*, organised as a "sensing-to-feedback" pipeline (data collection → preprocessing → analysis → feedback). The topic is timely for digital health and the pipeline framing is genuinely useful for readers who want to design end-to-end systems rather than isolated classifiers. The review is clearly written and (based on what is provided) follows PRISMA 2020 with a registered protocol in PROSPERO database and supporting files.

However, several issues require attention before the work would fully meet PLOS Digital Health expectations around open science, reproducibility, and complete systematic review reporting. The biggest gaps relate to (i) data/materials availability for the review itself, (ii) full transparency of database-specific search strategies and protocol availability, and (iii) clarity/consistency in reporting (e.g., number of databases, language restrictions).

b) Fit for PLOS Digital Health

Overall fit: good, because the review addresses digital measurement of health-relevant behaviours (wearables, algorithms, and translation to feedback), and explicitly highlights real-world deployment barriers (heterogeneity, limited feedback implementations, privacy considerations).

To align even more tightly with PLOS Digital Health's publication criteria (rigour, evidence for conclusions, clear utility/accessibility, and appropriate open science practice), the manuscript should strengthen reproducibility and "usable outputs" (share full extracted dataset + structured summary tables + clearer protocol/search transparency). (PLOS Digital Health reviewer guidelines)

c) Major comments

1) Data availability and open-science compliance (high priority)

The Data Availability Statement indicates that only a subset of extracted data is provided, and the full extraction form is "available upon request".

PLOS policy expects the *minimal dataset* needed to replicate findings to be publicly available at publication, with restrictions only when ethically/legal necessary. For a systematic review, the extracted data, risk-of-bias judgements, and analysis tables are typically not sensitive and should be shared (e.g., as supporting files or in a repository with a DOI). (PLOS Digital Health data availability policy)

What to change:

- Deposit the *complete* extraction sheet (all included studies, all extracted fields listed in Table 1) plus the full risk-of-bias scoring per study/domain (not only a figure summary).
- If any element truly cannot be shared, state the specific restriction and a non-author-controlled access mechanism, consistent with PLOS guidance. (PLOS Digital Health data availability policy)

2) Protocol availability and reporting

The manuscript states that the protocol is registered in the PROSPERO registry under registration number (protocol record ID) CRD42025637387. PLOS Digital Health submission guidance for systematic reviews asks authors to indicate whether a protocol exists and, if so, provide it as supporting information and include the registry record number in the abstract. (PLOS Digital Health submission guidelines)

What to change:

- Add the protocol registration number/record ID CRD42025637387 on PROSPERO to the abstract (it currently appears only in the main text).
- Provide the protocol as a supporting file (e.g., exported from PROSPERO database / dated protocol document) and describe any deviations from the registered protocol.

3) Search strategy transparency (database-by-database)

Supporting Information 2 provides the Web of Science strategy and states syntax was adapted for other databases.

For reproducibility, systematic reviews should provide the full strategies (or at minimum the exact final queries) for **each** database searched, including limits/filters used. PLOS also expects robust, transparent reporting in systematic reviews. (PLOS Digital Health submission guidelines; PLOS Digital Health reviewer guidelines)

What to change:

- Provide complete search strings for all databases listed (PubMed/MEDLINE, EMBASE, CINAHL, SPORTDiscus, IEEE Xplore, ACM, Scopus, ProQuest, etc.), along with the exact run dates for the initial and updated searches.
- The manuscript states the initial search was conducted on **12 January 2025** and that a **refined search strategy** was re-run on **4 December 2025**. This implies the search strings and/or limits were modified between the initial and updated searches, yet only one set of search terms/strategy is provided. Please explicitly report (i) the **original** search strategy used in January, (ii) the **refined** strategy used in December, and (iii) a brief description of **what was changed and why** (e.g., added/removed keywords, revised Boolean logic, altered subject headings, added filters, changes in databases searched). For full reproducibility, provide the exact final queries per database for both runs (or clearly state that the strategy was unchanged and remove "refined" if no refinement occurred). Please also report the **number of records retrieved per query and per database** for the January search and the December update (before de-duplication), ideally in a supplemental table alongside the exact queries and run dates for each database.
- The authors state that the updated search was not re-run in Engineering Village (EI) because the initial EI results were all duplicates. Given the 11-month interval between the initial search (January) and the update (December), please justify the assumption that EI would again yield only duplicates. Specifically, report (i) the number of EI records retrieved initially and the proportion identified as duplicates, (ii) the basis for concluding EI indexing/coverage relevant to this topic would not change over the update period, and (iii) whether a limited EI update search (or a spot-check) was conducted to confirm no new eligible records were added during those 11 months.

4) Internal consistency: number of databases and language restrictions

- The abstract states that records were identified across **nine** scientific databases, whereas the Methods list **ten** databases searched. Please clarify this discrepancy explicitly by stating that **ten databases were searched initially**, but records contributing to the final set came from **nine**, because **Engineering Village yielded only duplicates** and was therefore not re-run (and/or did not contribute unique records after de-duplication). Please ensure the **abstract, methods, and PRISMA flow/summary counts** are consistent and unambiguous.
- The Methods state that the updated search was re-run with "no restrictions on language", but the eligibility criteria require full-text articles published in **English**, and the limitations also state an English-only restriction. Please correct or explain this inconsistency. If the searches were conducted without language limits but inclusion was restricted to English at the screening/full-text stage, state this explicitly and ensure it aligns with the protocol; as the

protocol specified English-only, clarify that searching without language limits represents a deviation and, if so, justify it.

What to change:

- Harmonise the database count everywhere (abstract, methods, PRISMA flow narrative).
- Correct the "no language restrictions" statement, or explain that the initial retrieval was unrestricted but inclusion was English-only, and justify why the search was unrestricted with respect to language.

5) PRISMA completeness: excluded full-text studies and reasons

In the PRISMA checklist, item 16b ("cite studies that might appear to meet inclusion criteria, but were excluded, and explain why") is marked "Not applicable".

The PRISMA flow diagram appropriately reports the **numbers and categories** of full-text exclusions (n=126). However, PRISMA checklist item **16b** relates to **citing** excluded studies that might appear to meet inclusion criteria and explaining why they were excluded.

What to change:

- Please either (i) provide a supplementary list of excluded full-text articles (at least the borderline/near-miss papers) with the primary exclusion reason, **or** (ii) revise the PRISMA checklist entry (16b) to accurately reflect what was (and was not) reported and justify why a citation list was not provided.

6) Risk-of-bias / quality assessment tool needs more operational detail

You created a tailored tool based on QUADAS-AI with five binary domains (0/1).

Supporting Information 3 is currently very high-level (one question per domain) and does not provide sufficient scoring guidance or decision rules, and the manuscript does not report inter-rater agreement (e.g., kappa) for the risk-of-bias assessment.

There is also a *narrative tension* where reference criteria are described as "often under-reported", yet "methodological transparency" is scored 100% across studies. This requires clarification of what "methodological transparency" covered and how borderline cases were handled.

What to change:

- Expand the tool into an operational rubric: what counts as "Yes/No" for each domain, examples, and how you handled unclear reporting.
- Provide per-study domain-level ratings in a table (not just aggregated percentages / a heatmap figure).
- Report reviewer agreement and how conflicts were resolved.

7) Strength of conclusions vs. heterogeneity (avoid over-claiming)

You state that deep learning "demonstrated superior performance" and that ML "consistently outperformed" threshold-based approaches.

Given that you did not perform meta-analysis and emphasise substantial heterogeneity, these statements should be qualified carefully (e.g., "in the included comparisons where evaluated", "in specific datasets/settings", "limited head-to-head evidence").

What to change:

- Where making comparative performance claims, point readers to the exact studies and summarise the comparison context (sensor placement, population, ground truth, window

length, outcome definition). A structured table of performance outcomes by task would greatly help (even if qualitative).

d) Minor comments

1. **Terminology and copy-editing:**

- Correct the phrase “with no restrictions language” (missing “on”).
- Ensure consistent spelling of “behaviour/behavior” throughout (journal style may accept either, but consistency matters).
- Consider tightening definitions around “prolonged posture monitoring” vs “sedentary behaviour monitoring” early, and ensure inclusion criteria operationalise that distinction clearly.

2. **Support files labelling:** Supporting Information 2 is described as the “final complete Web of Science search strategy”; consider retitling it explicitly as “Web of Science strategy (and list of databases searched)” unless you add all database queries.
3. **Reproducibility markers:** Fig 3 includes elements like “codes available” and “dataset availability” across included studies. Consider defining precisely what counted as “available” vs “by request” and whether this was verified at extraction time.

e) Recommendations

Recommendation to the editor: Major revision.

Rationale: the manuscript is relevant and potentially impactful for PLOS Digital Health, but requires revisions to meet key expectations around **open science** (share the full extracted dataset and risk-of-bias table), **systematic review reproducibility** (full database search strings, protocol availability), and **consistency/clarity of reporting** (database count and language restriction statements). (PLOS Digital Health reviewer guidelines; PLOS Digital Health data availability policy; PLOS Digital Health submission guidelines)

If the authors address the items in Major Comments, the review would be much more reusable to the community and better aligned with PLOS Digital Health criteria on methodological rigour, evidence support, and open science. (PLOS Digital Health reviewer guidelines)
