# Supplementary material for: A systematic review of prolonged posture monitoring with accelerometer-based wearable sensors
Source: PLOS Digit Health. 2026 Sep 15;5(9):e0001538. doi: 10.1371/journal.pdig.0001538 (PMC13577576; doi:10.1371/journal.pdig.0001538)
Supplement: Supplementary file 6 [file pdig.0001538.s010.pdf]

Dear Editor and Reviewers,

We sincerely thank you for your careful evaluation of our manuscript and for the constructive comments. The feedback was highly valuable in helping us clarify the scope of the review, strengthen the methodological reporting, and better distinguish prolonged posture monitoring from broader sedentary behaviour monitoring.

In response, we have substantially revised the manuscript. The main revisions include:

- clarifying the operational definition of prolonged posture and its distinction from sedentary behaviour;
- re-extracting in the databases to ensure a timely review;
- re-screening the included studies using the revised eligibility criteria;
- restructuring the Results and Discussion to separate descriptive findings from interpretation;
- expanding the risk-of-bias assessment with operational rubrics, per-study judgments, and inter-rater agreement;
- preparing extensive supplements to ensure transparency;
- adding structured summaries of algorithm performance and validation context;
- strengthening the implications for future research and digital health practice.

Below, we provide a point-by-point response to each comment. Reviewer comments are shown in bold, followed by our responses and a summary of the corresponding manuscript changes.

Editors & Reviewer 2 (highlighted in light orange in the manuscript)

Major comment 1: Data availability and open-science compliance (high priority)

The Data Availability Statement indicates that only a subset of extracted data is provided, and the full extraction form is “available upon request”.

PLOS policy expects the minimal dataset needed to replicate findings to be publicly available at publication, with restrictions only when ethically/legal necessary. For a systematic review, the extracted data, risk-of-bias judgements, and analysis tables are typically not sensitive and should be shared (e.g., as supporting files or in a repository with a DOI). (PLOS Digital Health data availability policy)

What to change:

- Deposit the complete extraction sheet (all included studies, all extracted fields listed in Table 1) plus the full risk-of-bias scoring per study/domain (not only a figure summary).
- If any element truly cannot be shared, state the specific restriction and a non-author-controlled access mechanism, consistent with PLOS guidance. (PLOS Digital Health data availability policy)

Response

Thank you for the suggestions, we have provided the complete extraction sheets and risk-of-bias scoring in supporting information (page 30):

S4 File. Excluded studies. List of studies excluded during full-text screening, with primary reasons for exclusion.

S5 File. Data extraction. Complete data extraction for all 14 included studies.

S7 File Risk of bias assessment operational rubrics and per-study judgment. This supplement provides the operational rubrics applied in the risk of bias assessment, and the per-item judgments made by both reviewers for each study.

Major comment 2: Protocol availability and reporting

The manuscript states that the protocol is registered in the PROSPERO registry under registration number (protocol record ID) CRD42025637387. PLOS Digital Health submission guidance for systematic reviews asks authors to indicate whether a protocol exists and, if so, provide it as supporting information and include the registry record number in the abstract. (PLOS Digital Health submission guidelines)

What to change:

- Add the protocol registration number/record ID CRD42025637387 on PROSPERO to the abstract (it currently appears only in the main text).
- Provide the protocol as a supporting file (e.g., exported from PROSPERO database / dated protocol document) and describe any deviations from the registered protocol.

Response

Thank you for your suggestion. We have revised the manuscript to enhance protocol transparency in accordance with PLOS Digital Health guidelines.

We have now included the PROSPERO registration number (CRD42025637387) in the **abstract**:

PROSPERO registration: CRD42025637387.

Based on the revisions, we updated the PROSPERO protocol on April 18, 2026, to ensure alignment with our latest revision. Following the suggestions we received, we revised the **Materials and Methods - Protocol Registration** section to include a citation to PROSPERO (page 5, lines 123-129) and introduced the latest protocol version as supporting information (S2 File):

"The review protocol is registered with the International Prospective Register of Systematic Reviews (PROSPERO) under ID CRD42025637387. The initial registration was submitted on February 5, 2025, and last updated on April 18, 2026 [25]. Updates were made in accordance with the manuscript revisions, and update notes are accessible in the PROSPERO registration. The most recent protocol registration (version 5.0) is provided in S2 File."

[25] Liao M, Huang K, Libeert E. A Systematic Review of Prolonged Posture Monitoring with Accelerometer-Based Wearable Sensors; 2025. CRD42025637387. PROSPERO 2025. Available from: https://www.crd.york.ac.uk/PROSPERO/view/CRD42025637387.

Accordingly, we have added the **S2 File** to the **Supporting information** section (page 30):

"**S2 File PROSPERO registration.** Study protocol registered in PROSPERO (CRD42025637387), last updated on April 18, 2026 (Version 5.0)."

We believe these revisions improve transparency and reproducibility of the review.

Major comment 3: Search strategy transparency (database-by-database)

Supporting Information 2 provides the Web of Science strategy and states syntax was adapted for other databases.

For reproducibility, systematic reviews should provide the full strategies (or at minimum the exact final queries) for each database searched, including limits/filters used. PLOS also expects robust, transparent reporting in systematic reviews. (PLOS Digital Health submission guidelines; PLOS Digital Health reviewer guidelines)

What to change:

- Provide complete search strings for all databases listed (PubMed/MEDLINE, EMBASE, CINAHL, SPORTDiscus, IEEE Xplore, ACM, Scopus, ProQuest, etc.), along with the exact run dates for the initial and updated searches.
- The manuscript states the initial search was conducted on 12 January 2025 and that a refined search strategy was re-run on 4 December 2025. This implies the search strings and/or limits were modified between the initial and updated searches, yet only one set of search terms/strategy is provided. Please explicitly report (i) the original search strategy used in January, (ii) the refined strategy used in December, and (iii) a brief description of what was changed and why (e.g., added/removed keywords, revised Boolean logic, altered subject headings, added filters, changes in databases searched). For full reproducibility, provide the exact final queries per database for both runs (or clearly state that the strategy was unchanged and remove “refined” if no refinement occurred). Please also report the number of records retrieved per query and per database for the January search and the December update (before de-duplication), ideally in a supplemental table alongside the exact queries and run dates for each database.
- The authors state that the updated search was not re-run in Engineering Village (EI) because the initial EI results were all duplicates. Given the 11-month interval between the initial search (January) and the update (December), please justify the assumption that EI would again yield only duplicates. Specifically, report (i) the number of EI records retrieved initially and the proportion identified as duplicates, (ii) the basis for concluding EI indexing/coverage relevant to this topic would not change over the update period, and (iii) whether a limited EI update search (or a spot-check) was conducted to confirm no new eligible records were added during those 11 months.

Response

We fully acknowledge the importance of transparent and reproducible reporting of search strategies. Accordingly, we have revised **S3 File** (previously S2 File) to provide comprehensive database-specific search strategies and increased transparency regarding search updates.

Specifically, we have made the following revisions:

1. Database-specific search strategies: We now provide complete search strings for all ten databases. Each includes database-specific syntax, Boolean operators, and any applied limits to ensure reproducibility.
2. Clear reporting of search dates: We now explicitly report the run dates for all three searches: the initial search on January 12, 2025, the updated search on December 4, 2025, and the catch-up search on April 8, 2026.
3. Original and refined search strategies: In S3 File, we now describe all refinements to the search strategy and the rationale for these refinements. These changes were implemented to improve sensitivity in response to reviewer feedback from a previous submission.
4. Records retrieved per database: In S3 File, we have added Table I, which reports the number of records retrieved per database for each search run (January 2025, December 2025, and April 2026) prior to deduplication.
5. Engineering Village (EI) clarification: Engineering Village was not re-searched in December 2025 because its results overlapped with those of other databases in the initial search. To ensure consistency and completeness, it was re-searched during the catch-up search, and the retrieved records are now reported.

Besides, in **Materials and methods – Information source and search strategy** section, we have added more detailed descriptions of the procedures (page 5, lines 130 – 165). We believe these revisions enhance transparency and reproducibility in accordance with PLOS Digital Health and systematic review reporting guidelines.

Major comment 4: Internal consistency: number of databases and language restrictions

- The abstract states that records were identified across nine scientific databases, whereas the Methods list ten databases searched. Please clarify this discrepancy explicitly by stating that ten databases were searched initially, but records contributing to the final set came from nine, because Engineering Village yielded only duplicates and was therefore not re-run (and/or did not contribute unique records after de-duplication). Please ensure the abstract, methods, and PRISMA flow/summary counts are consistent and unambiguous.
- The Methods state that the updated search was re-run with “no restrictions on language”, but the eligibility criteria require full-text articles published in English, and the limitations also state an English-only restriction. Please correct or explain this inconsistency. If the searches were conducted without language limits but inclusion was restricted to English at the screening/full-text stage, state this explicitly and ensure it aligns with the protocol; as the protocol specified English-only, clarify that searching without language limits represents a deviation and, if so, justify it.

What to change:

- Harmonise the database count everywhere (abstract, methods, PRISMA flow narrative).
- Correct the "no language restrictions" statement, or explain that the initial retrieval was unrestricted but inclusion was English-only, and justify why the search was unrestricted with respect to language.

Response

Thank you for highlighting these inconsistencies. We have revised the manuscript to improve clarity and ensure consistency across the abstract, Methods, and PRISMA flow diagram.

- Database count: In the initial search in January 2025, we included Engineering Village, while in the updated search in December 2025, Engineering Village was not re-run because the initial Engineering Village results showed substantial overlap with other databases. However, to ensure completeness and transparency, Engineering Village was subsequently included in the April 8, 2026 catch-up search, as reflected in the revised PRISMA flow diagram. We clarified that ten databases were searched in total, including Engineering Village. The abstract, Methods, and PRISMA flow diagram have been revised to report this consistently.
    - **Abstract**:

      "Ten databases, including Web of Science, Scopus, ProQuest Central, PubMed/MEDLINE, EMBASE, CINAHL, SPORTDiscus, IEEE Xplore, ACM Digital Library, and Engineering Village, were searched."

      "From 27,334 records, 14 studies were included."

    - **Materials and methods -- Information source and search strategy** (page 5, lines 140-143, 151-155):

      "The initial literature retrieval was conducted on January 12, 2025, in ten databases: Web of Science (SCI-EXPANDED, SSCI, AHCI, CPCI-S, CPCI-SSH, ESCI), Scopus, ProQuest Central, PubMed (including MEDLINE), EMBASE, CINAHL, SPORTDiscus, IEEE Xplore, ACM Full-text Collection, and Engineering Village (EI). "

      The refined search strategy was re-executed on December 4, 2025, across all databases except EI, as its results were duplicated in the other nine databases. To ensure completeness and maintain a timely review, an additional catch-up search was conducted on April 8, 2026, across all ten databases used in the initial search in January 2025, including EI. "

    - PRISMA flow diagram (**Fig 2**)
    - **S3 File** description (page 30)

"This supplement presents the full search strategies used for each database, including Web of Science, Scopus, ProQuest Central, PubMed, Embase, CINAHL, SPORTDiscus, IEEE Xplore, ACM Digital Library, and Engineering Village (EI). It provides the detailed strategies for the initial search (January 12, 2025), the updated search (December 4, 2025), and the catch-up search (April 8, 2026), the number of records retrieved from each database before deduplication, and the modifications in each search."

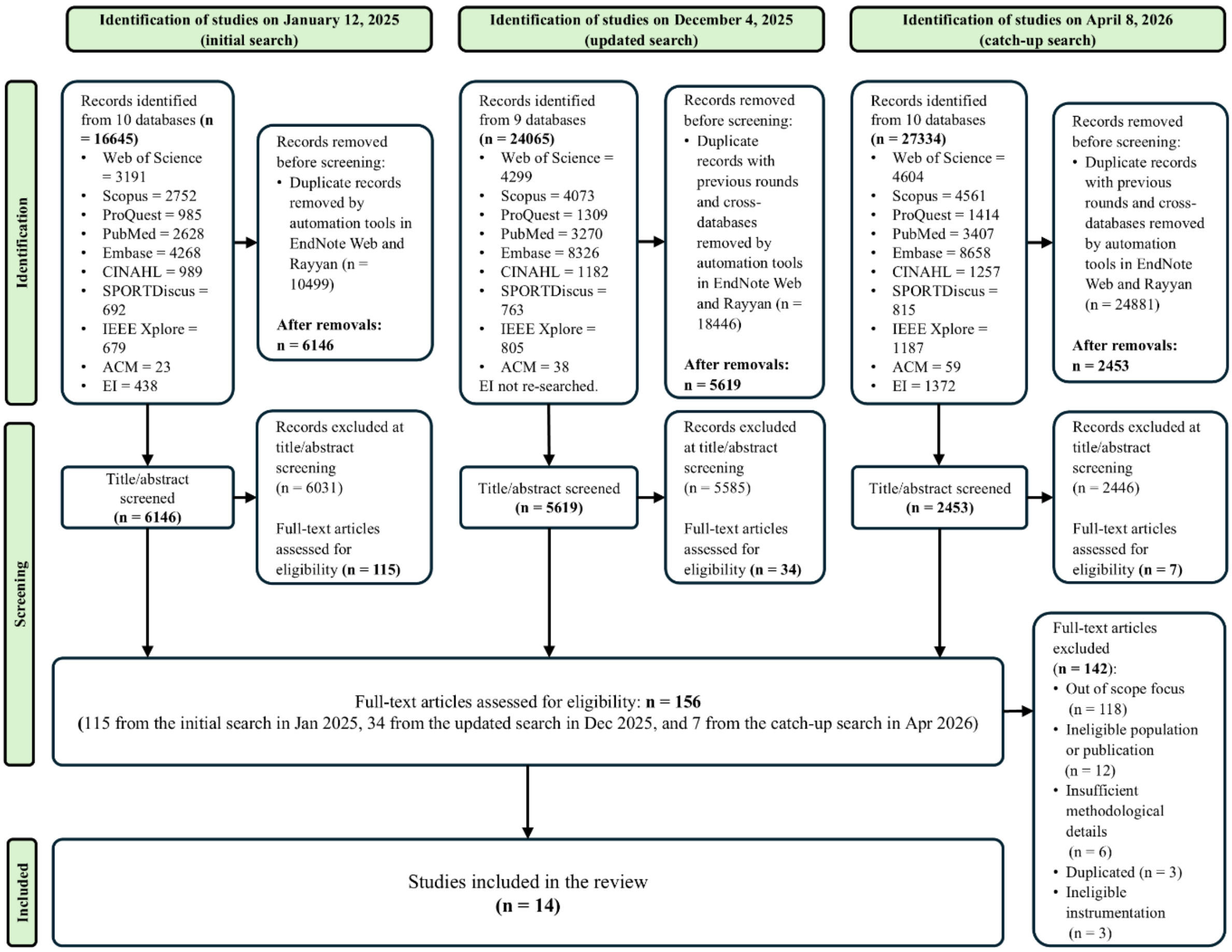

Fig 2 PRISMA flowchart of the results from the literature search.

- Language limit: We also clarified that no language limits were applied during database searching in order to maximize search sensitivity and reduce the risk of missing relevant studies. While, in consistent with the eligibility criteria and the registered protocol (PROSPERO CRD42025637387), only full-text articles published in English were eligible for inclusion and the ineligible articles were excluded manually during title/abstract screening. We have refined the description in **Materials and methods - Information source and search strategy** section (page 5, lines 160-165):

"All retrieved records from each search round were screened using the same eligibility criteria described in the following subsection. No language or publication date restrictions were applied during database searching. Articles published in languages other than English were excluded during title and abstract screening. In addition, reference lists of relevant reviews and included studies were screened to identify potentially eligible articles."

We believe these revisions harmonize the database reporting and clarify the language restriction policy across the abstract, Methods, and PRISMA diagram.

Major comment 5: PRISMA completeness: excluded full-text studies and reasons

In the PRISMA checklist, item 16b ("cite studies that might appear to meet inclusion criteria, but were excluded, and explain why") is marked "Not applicable".

The PRISMA flow diagram appropriately reports the numbers and categories of full-text exclusions (n=126). However, PRISMA checklist item 16b relates to citing excluded studies that might appear to meet inclusion criteria and explaining why they were excluded.

What to change:

- Please either (i) provide a supplementary list of excluded full-text articles (at least the borderline/near-miss papers) with the primary exclusion reason, or (ii) revise the PRISMA checklist entry (16b) to accurately reflect what was (and was not) reported and justify why a citation list was not provided.

Response

We have provided a supplementary list of excluded full-text articles with the primary exclusion reason in **S4 File**, the PRISMA checklist item 16b (**S1 File**) has been changed accordingly.

Major comment 6: Risk-of-bias / quality assessment tool needs more operational detail

You created a tailored tool based on QUADAS-AI with five binary domains (0/1).

Supporting Information 3 is currently very high-level (one question per domain) and does not provide sufficient scoring guidance or decision rules, and the manuscript does not report inter-rater agreement (e.g., kappa) for the risk-of-bias assessment.

There is also a narrative tension where reference criteria are described as "often under-reported", yet "methodological transparency" is scored 100% across studies. This requires clarification of what "methodological transparency" covered and how borderline cases were handled.

What to change:

- Expand the tool into an operational rubric: what counts as "Yes/No" for each domain, examples, and how you handled unclear reporting.

- Provide per-study domain-level ratings in a table (not just aggregated percentages / a heatmap figure).
- Report reviewer agreement and how conflicts were resolved.

Response

After refining the definition of prolonged postures, the inclusion/exclusion criteria have been revised. Accordingly, we re-conducted the screening, and the included articles were changed. As a consequence, we now apple the validated tools instead of a self-developed tool. To ensure the transparency, we have provided the operational rubric and the per-study per-item rating and rationale in **S7 File**.

Major comment 7: Strength of conclusions vs. heterogeneity (avoid over-claiming)

You state that deep learning "demonstrated superior performance" and that ML "consistently outperformed" threshold-based approaches.

Given that you did not perform meta-analysis and emphasise substantial heterogeneity, these statements should be qualified carefully (e.g., "in the included comparisons where evaluated", "in specific datasets/settings", "limited head-to-head evidence").

What to change:

- Where making comparative performance claims, point readers to the exact studies and summarise the comparison context (sensor placement, population, ground truth, window length, outcome definition). A structured table of performance outcomes by task would greatly help (even if qualitative).

Response

Thank you for this important comment. We have completely revised the manuscript to avoid over-claiming and now qualify all comparative performance statements. To make the comparison more transparent and straightforward, we added a structured summary table **(Table 2, page 20)** of machine learning studies. The table reports the population, test sensor and placement, reference criterion, model, analytical task, and key reported performance metrics for each study. This allows readers to interpret performance claims in context rather than as pooled or generalizable estimates. We also revised the **Results, Discussion, Abstract, and Conclusion** to emphasize that no meta-analysis was performed and that external validation is required before deployment in individual-level digital health applications.

Minor comment 1: Terminology and copy-editing

- Correct the phrase "with no restrictions language" (missing "on").
- Ensure consistent spelling of "behaviour/behavior" throughout (journal style may accept either, but consistency matters).
- Consider tightening definitions around "prolonged posture monitoring" vs "sedentary behaviour monitoring" early, and ensure inclusion criteria operationalise that distinction clearly.

Response

- Following the suggestion of Major comment 5, we have revised the corresponding part, this phrase has been replaced (lines 161 – 163, page 5):

  "No language or publication date restrictions were applied during database searching. Articles published in languages other than English were excluded during title and abstract screening."

- We have checked all the spellings and kept consistent with "behaviour". Similarly, we have checked all the spellings are in British English.
- We have defined the "prolonged posture monitoring" and "sedentary behavior monitoring" in the introduction and updated the inclusion criteria accordingly to reflect this distinction:
  - Definition of prolonged posture monitoring in this study (lines 31-33, page 2):

    "Given the wide range of duration thresholds, to maximize the inclusion of relevant studies, this systematic review operationally defines prolonged posture as a sitting or standing bout lasting at least 10 minutes without interruption during wake time."

  - Definition of sedentary behaviour introduced in the **Introduction** (lines 11-13):

    "The Sedentary Behaviour Research Network defines sedentary behaviour as any waking behaviour characterized by an energy expenditure ⩽1.5 metabolic equivalents while in a sitting, reclining, or lying posture [4]."

    [4] Tremblay MS, Aubert S, Barnes JD, Saunders TJ, Carson V, Latimer-Cheung AE, et al. Sedentary behavior research network (SBRN)–terminology consensus project process and outcome. International journal of behavioral nutrition and physical activity. 2017;14(1):75. doi:10.1186/s12966-017-0525-8.

  - **Inclusion criteria** item 1 (lines 170-174, page 6):

    "Monitor prolonged sitting and/or standing bouts:

    The study focused on or reported the monitoring of prolonged sitting and/or standing bouts during wake time. For the purposes of this review, prolonged posture is defined as uninterrupted sitting and/or standing bouts lasting at least 10 minutes."

Minor comment 2: Support files labelling

Supporting Information 2 is described as the “final complete Web of Science search strategy”; consider retitling it explicitly as “Web of Science strategy (and list of databases searched)” unless you add all database queries.

Response

Following the revision based on Major comment 3, we have revised Supporting Information 2 to include the complete search strategies for all ten databases searched, rather than only the Web of Science strategy. The supporting file now provides database-specific queries, run dates, and retrieved record counts for Web of Science, Scopus, ProQuest Central, PubMed, Embase, CINAHL, SPORTDiscus, IEEE Xplore, ACM Digital Library, and Engineering Village (EI). The file title and description in **Supporting information** section in the manuscript have been updated accordingly:

S3 File. Full search strategies for all databases. This supplement presents the full search strategies used for each database, including Web of Science, Scopus, ProQuest Central, PubMed, Embase, CINAHL, SPORTDiscus, IEEE Xplore, ACM Digital Library, and Engineering Village (EI). It provides the detailed strategies for the initial search (January 12, 2025), the updated search (December 4, 2025), and the catch-up search (April 8, 2026), the number of records retrieved from each database before deduplication, and the modifications in each search.

Minor comment 3: Reproducibility markers

Fig 3 includes elements like “codes available” and “dataset availability” across included studies. Consider defining precisely what counted as “available” vs “by request” and whether this was verified at extraction time.

Response

During the revision, we have replaced Fig 3 to **S6 File** and renamed the categories (see below). We carefully verified the articles and added (verified) in the data extraction (**S5 File**) with links to the resources. For example, we noted in S5 File – General Information - Reporting items for Tjurin et al.: 'Open-sourced code and implementation vignettes available at https://adalabucsd.github.io/DeepPostures/ (verified). No published protocol/trial registration reported in the manuscript.'

S6 File categories:

**Reporting items**	Codes availability	Cited the open source libraries / models / tools
		Original codes available online
		No statement on codes availability
	Dataset availability	Publicially available
		Upon request
		Restricted
		No statement on dataset availability
	Protocol / trial registration	Published protocol /trial registration
		Not available or applicable

Reviewer 1 (highlighted in blue in the manuscript)

Introduction

Comment 1

The definition of “prolonged” is not clear enough. The authors need to specify a time range for prolonged postures (e.g., 10 to 30 minutes) as the focus of this review, and align this definition with guidelines from the Sedentary Behavior Research Network (SBRN). In addition, it is needed to introduce the definitions of prolonged sitting or standing used in previous studies, and compare them to highlight the focus of the current review.

Response

Thank you for this helpful comment. We agree that the term "prolonged"' was used inconsistently in the literature and that the review needed a clearer operational definition. We revised the Introduction and Eligibility criteria to explicitly distinguish the SBRN definition of sedentary behaviour from the bout-based definition used in this review. Because SBRN does not specify a single universal duration threshold for "prolonged" sitting or standing, we operationalized prolonged posture for this review as uninterrupted sitting or standing during wake time lasting at least 10 minutes.

We also expanded the Introduction to summarize thresholds used in previous studies, including 5, 10, 20, 30, and 60 minutes, and clarified that the focus of this review is not a single universal threshold but the monitoring of uninterrupted posture bouts that meet or exceed a minimum 10-minute duration. This lower-bound threshold was selected to capture studies that explicitly operationalized prolonged posture while excluding generic sedentary behaviour or posture-classification studies without prolonged-bout reporting. The Results section now compares the definitions used across included studies, and the Discussion explicitly identifies definitional heterogeneity as a central barrier to comparability and digital health translation.

- **Introduction**:

"Studies have used different duration thresholds to characterise prolonged sitting or standing, ranging from 5 or 10 minutes to 20, 30, or 60 minutes, depending on the study objective and application [5, 6, 8–12]." (lines 24-26, page 2)

"Given the wide range of duration thresholds, to maximise the inclusion of relevant studies, this systematic review operationally defines prolonged posture as a sitting or standing bout lasting at least 10 minutes without interruption during wake time. Although some studies have used shorter thresholds, such as 5 minutes [9], 10 minutes is considered more appropriate for representing prolonged posture in the context of this review. The focus is on sitting and standing, as these postures are most directly associated with occupational and daily-life musculoskeletal exposures." (lines 31 – 37, pages 2-3)

- **Results - Prolonged posture characteristics and reported outcomes** (lines 348-356, page 10):

"The threshold for defining a prolonged bout varied across studies, with ⩾ 30 minutes of uninterrupted sitting being the most frequently used criterion, appearing in eight studies [13, 15, 26, 30, 32, 34, 36, 37]. Other thresholds included ⩾ 5 and ⩾ 10 minutes [9], ⩾ 20 minutes [30, 36], and ⩾ 60 minutes [26, 35]. Three studies did not specify exact thresholds for prolonged bouts but instead reported multiple bout duration categories [31, 33, 38]. Notably, one study categorized uninterrupted bouts for both sitting and time on feet into bins of: <1, 1 to 2, 2 to 3, 3 to 4, 4 to 5, 5 to 10, 10 to 30, 30 to 60, and >60 minutes, and further grouped them as short (⩽ 5 min), moderate (>5 to ⩽ 30 min), and long (>30 min) [14]."

Methods and Results

Comment 1

The literature search yields over 24k records from 10 databases, but only 23 studies are found to be eligible for inclusion. This large discrepancy may stem from overly broad search terms; for example, “data analysis” is too general and unnecessary for targeting data analysis methods. Thus, the search strategy can be refined to narrow down the relevant records more efficiently.

Response

Thank you for this thoughtful comment. We recognize that the number of retrieved records is high relative to the included studies, and we addressed this during the development of our search strategies. To ensure methodological rigor and comprehensive database coverage, we consulted experienced biomedical engineering librarians and colleagues with engineering and clinical expertise. Because this systematic review aims to cover the entire sensing-to-feedback pipeline and facilitate interdisciplinary collaboration, we included a broad range of potentially relevant terms to minimize the risk of missing relevant studies, especially in interdisciplinary areas such as wearable sensing and digital health, where terminology is heterogeneous and inconsistently reported.

We included broad terms such as “data analysis” and “data analyses” to capture studies that describe analytical approaches without explicitly referencing artificial intelligence or machine learning, reducing the risk of missing eligible studies. To evaluate the impact of these terms, we removed them from the search strategy on April 8, 2026. For example, in Embase, results decreased from 8,658 to 8,542 (1.3%); in Web of Science, from 4,604 to 4,391 (4.6%); in Scopus, from 4,561 to 4,367 (4.3%); and in PubMed, from 3,407 to 3,196 (6.2%). This marginal change suggests these terms were not the primary contributors to the high number of retrieved records.

We consider that this high retrieval volume mainly reflects the interdisciplinary scope of the topic and the large overlap across ten databases. As shown in the PRISMA flow diagram, 27,334 records were initially retrieved from ten databases. Of these, 153 full-text articles were assessed for eligibility, and 14 studies met the inclusion criteria. This stepwise reduction demonstrates the use of strict eligibility criteria rather than insufficient search specificity. Nevertheless, we have analyzed this as a limitation in the **Discussion – Limitations** (lines 1176-1184, page 29):

"The search strategy was intentionally designed to prioritise high sensitivity over high specificity. The large number of records identified, relative to the few studies meeting the inclusion criteria,

reflects the broad application of the term “sedentary”, which covers a wider range of topics, including non-postural sedentary behaviours, screen time, and self-report research, compared to the narrower focus on prolonged posture monitoring in this review. This approach was chosen because prolonged posture monitoring is not consistently indexed as a distinct topic, and prioritising sensitivity reduces the risk of omitting relevant studies, even if it results in retrieving many irrelevant records."

In response to the comments received during this revision, we conducted a catch-up search on April 8, 2026, to ensure the manuscript remains current. To maintain consistency across search rounds and avoid introducing additional variation to further strategy changes, we used the same refined search terms as in December. We also standardized the Boolean logic structure to (title(concept 1) OR abstract(concept 1) OR keyword(concept 1)) AND (title(concept 2) OR abstract(concept 2) OR keyword(concept 2)) AND (title(concept 3) OR abstract(concept 3) OR keyword(concept 3)) across all databases to keep consistency and to include more studies.

We have provided detailed explanation on our database searching in **Materials and methods – Information source and search strategy** section. In addition, we clarified the search rationale and provided the complete database-specific search strategies used in all search rounds in the updated **S3 File**.

We hope this response addresses the concerns.

Comment 2

While various sensors (e.g., activPAL, ActiGraph) are discussed, their accuracy in monitoring prolonged postures across different populations (e.g., healthy adults, older adults, clinical groups) and settings is not clearly compared. The analytical depth of this review can be improved if the authors use examples from the included studies to support such comparisons.

Response

Thank you for this comment. We agree that the previous version described sensor use but did not sufficiently compare how monitoring performance differed across populations and settings.

We revised the **Results** section to provide a more structured comparison of accelerometer-based monitoring approaches, especially for the validation and algorithm-development studies where quantitative performance metrics were reported. The revisions include:

- **Data collection - Reference criterion** (lines 476 – 481, page 13):

  "In general, across the reviewed studies, thigh-worn activPAL most frequently served as the reference criterion, while hip- or waist-worn accelerometers, particularly ActiGraph devices, were assessed as alternative measurement tools. As quantitative performance was influenced by the entire analytical pipeline rather than sensor hardware alone, comparative accuracy across populations and settings is presented in the Data analysis section."

- **Data analysis – machine learning methods**: we added a structured table (**Table 2**, page 22) summarizing the machine learning and validation studies by population, setting, test sensor and placement, reference criterion, analytical model, monitoring task, and key performance outcomes. This table now allows readers to compare, for example, waist-worn ActiGraph validation in office workers, hip-worn ActiGraph validation in breast cancer survivors, CHAP validation in older adults, CHAP-Adult validation in adults aged 35 years and older, and CHAP-child validation in children.

- **Discussion**: we emphasized that sensor outputs from thigh-, hip-, and waist-worn configurations should not be considered interchangeable unless validated against a common reference criterion in the target population:

"The current evidence base is limited in both population and setting coverage. Most included studies have been conducted in Western, adult, office-based populations. Without inclusive validation, digital health systems may produce outputs that appear precise but are inaccurate for intended users, particularly when algorithms are developed or validated in narrow populations and then applied across demographic, clinical, or contextual groups with different movement patterns [59–61]. Future studies should validate prolonged posture monitoring in populations where deployment is anticipated, including children, older adults, clinical groups, and occupations involving prolonged standing." (lines 997 – 1005, page 25)

"Practitioners should assess whether the device and placement are appropriate for the user population and the intended clinical or ergonomic application before implementing prolonged posture metrics in practice." (lines 1019 – 1021, page 25)

- **Future works**: we recommended external validation across children, older adults, clinical groups, and standing-intensive occupational settings (lines 1202 – 1204, page 29):

"The evidence base should be expanded beyond Western, office-based, adult populations to include children, older adults, clinical groups, standing-intensive workers, and non-Western settings."

Comment 3

Similarly, multiple algorithms (e.g., statistical methods, classical machine learning, deep learning) are used to detect, assess, classify, and predict prolonged postures, but their performance is not quantitatively assessed. The authors may use a more structured way to summarize the performance of different models.

Response

Thank you for this comment. We revised the entire **Data analysis** section and added a structured performance table (**Table 2, page 22**) for the studies that reported quantitative algorithm performance. The table now summarizes each study by population, test sensor and placement, reference criterion, analytical model, task, and key reported performance metrics.

We did not perform a pooled quantitative comparison because the included studies differed substantially in analytical task, population, sensor placement, reference criterion, preprocessing pipeline, window length, bout definition, and performance metric. For example, some studies evaluated sitting/non-sitting classification, others evaluated multi-class posture classification, sit-to-stand transition detection, prolonged-bout estimation, or derived sitting-pattern variables. Reported metrics also differed, including sensitivity, specificity, PPV, balanced accuracy, mean absolute percent error, bias, correlation, and agreement. Therefore, a meta-analysis or single quantitative ranking of algorithms would not be appropriate.

Instead, we now provide a structured qualitative comparison of model performance by task and context. We also revised the narrative to avoid over-claiming, stating that CHAP-based deep-learning models showed strong reported performance in the specific datasets and tasks where they were evaluated, while emphasizing that external validation is needed before generalizing these models to other populations, postures, devices, or real-time feedback applications.

Discussion

Comment 1

Much of the content in the Discussion section repeats information already presented in the Results. Please avoid restating descriptive findings, and focus on interpreting the results.

Response

Thank you for this comment. We agree and have substantially revised the Discussion to reduce repetition of Results. Descriptive details, including study counts, thresholds, sensor types, and model performance metrics, are now primarily reported in the Results and supplementary tables. The Discussion has been refocused on interpretation and implications, following the four-stage sensing-to-feedback pipeline. Each subsection now addresses what the findings mean for future research and digital health practice, including sensor-selection trade-offs, the effect of preprocessing choices on bout detection, application-specific validation requirements, and the gap between post-hoc monitoring and real-time feedback. Interpretive statements that were previously included in the Results have also been moved to the Discussion where appropriate.

Comment 2

The authors need to provide implications of the current findings for future research and practice in digital health, in particular, how the findings can be used to guide researchers and healthcare practitioners for their future work.

Response

Thank you for this important comment. We agree that the previous Discussion did not sufficiently translate the findings into implications for future research and practice. We have revised the **Discussion** to explicitly address how the findings can guide researchers, digital health developers, healthcare practitioners, and occupational health professionals. Specifically, we added practical implications throughout the four-stage sensing-to-feedback pipeline.

For **data collection**, we now discuss how sensor placement should be selected according to the monitoring objective and target population, and we caution that outputs from thigh-, hip-, and waist-worn devices should not be treated as interchangeable without population-specific validation:

"Risk of bias assessments highlight the importance of selecting an appropriate criterion." (lines 984 – 985, page 25)

"While these issues do not invalidate free-living monitoring, they highlight the need for transparent reporting of wear instructions, valid-wear criteria, attachment procedures, and data quality checks." (lines 994 – 996, page 25)

"The current evidence base is limited in both population and setting coverage. Most included studies have been conducted in Western, adult, office-based populations. Without inclusive validation, digital health systems may produce outputs that appear precise but are inaccurate for intended users, particularly when algorithms are developed or validated in narrow populations and then applied across demographic, clinical, or contextual groups with different movement patterns [59–61]. Future studies should validate prolonged posture monitoring in populations where deployment is anticipated, including children, older adults, clinical groups, and occupations involving prolonged standing." (lines 997 – 1005, page 25)

"Sampling rate should be treated as a deliberate design choice rather than a technical detail." (lines 1006 – 1007, page 25)

"Researchers should select and report sensor configurations that align with the monitoring objective." (lines 1015 – 1016, page 25)

"Practitioners should assess whether the device and placement are appropriate for the user population and the intended clinical or ergonomic application before implementing prolonged posture metrics in practice." (lines 1019 – 1021, page 25)

For **data preparation**, we highlight the need for reproducible reporting of sleep/non-wear handling, synchronization, epoching, feature extraction, and bout-construction rules, because these choices directly influence prolonged-bout estimates:

"Studies should therefore specify how sleep, non-wear, water-based activities, and partial wear were handled." (lines 1035 – 1036, page 26)

"Future validation work should report device initialisation, synchronisation, and drift checking as standard." (lines 1040 – 1041, page 26)

"For real-time feedback, the window therefore needs to balance sensitivity to emerging prolonged bouts with resistance to false alerts from brief signal fluctuations." (lines 1047 – 1049, page 26)

"The choice of feature extraction method should therefore align with the specific application, for example, transparency is crucial for clinical interpretation, while higher model complexity can be justified for predictive tasks when supported by validated data and appropriate interpretability methods [73, 74]." (lines 1058 – 1061, page 26)

"Studies should document their pipelines in reproducible detail, including software, parameters, synchronisation, sleep and non-wear handling, epoching, feature extraction, and bout

construction rules. For real-time applications, it is also important to distinguish preprocessing steps that are feasible online from those that are only possible post-hoc." (lines 1068 – 1072, page 26)

For **data analysis**, we emphasize that analytical performance should be assessed according to the intended decision context, distinguishing group-level exposure assessment from individual-level real-time feedback or clinical use:

"Measurement error may be acceptable for estimating group-level exposure if it is quantified and handled appropriately, whereas individual-level monitoring and real-time feedback require higher precision because errors can directly trigger inappropriate or missed interventions [75–77]." (lines 1077 – 1081, page 27)

"Consequently, validation studies should report performance separately for posture classification, transition timing, total sitting time, bout duration, and prolonged bout accumulation." (lines 1086 – 1088, page 27)

"External validation in the intended target population is therefore essential before deploying such models for individual-level digital health applications [59, 75, 76]." (lines 1094 – 1096, page 27)

"While several studies provided accuracy or error metrics, essential elements such as equivalence testing, Bland–Altman plots, limits of agreement, pre-specified validity thresholds, and categorical agreement statistics were often missing." (lines 1097 – 1100, page 27)

When complex models are implemented, providing open-source code, reporting subgroup performance, and documenting failure cases can enhance transparency, trust, and independent evaluation [73, 76]." (lines 1105 – 1107, page 27)

"In practical applications, analytical methods should align with the specific decision-making context." (lines 1108 – 1109, page 27)

"Therefore, analytical performance should be assessed in relation to the intended action, rather than relying exclusively on statistical metrics." (lines 1115 – 1117, page 27)

"Future work should prioritise application-specific validation of bout-level outcomes, transition timing, subgroup robustness, and real-time feasibility." (lines 1119 – 1120, page 27)

For **user feedback**, we clarify that monitoring alone does not constitute intervention, and that future digital health systems should evaluate feedback timing, personalization, acceptability, notification fatigue, and unintended posture substitution.

"Future studies should evaluate user acceptance, the risk of notification fatigue, long-term engagement, and any unintended shifts to alternative postures. The criteria for triggering feedback needs to be clearly justified, since acceptable bout lengths differ between epidemiological surveillance, workplace prevention, rehabilitation, and self-management contexts, and should be adaptable to individual requirements." (lines 1140 – 1144, page 28)

We also rewrote the **Future works** section to provide concrete directions: standing-specific bout monitoring, clearer definitions of "prolonged"', task-specific validation metrics, real-time feasibility testing, validation in diverse populations and settings, and transparent data-preparation pipelines. These revisions make the implications of the review more actionable for future research and for digital health or occupational health practice.

Reviewer 3 (highlighted in green in the manuscript)

Major comment 1 - Mixing prolonged posture and sedentary behaviour monitoring

The authors initially distinguish clearly between these two concepts; however, in the Results and Discussion they are used somewhat interchangeably. If studies on sedentary behaviour were critically evaluated and considered as posture monitoring for the purpose of this review, this should be explicitly justified. A clear separation between sedentary behaviour, including the energy expenditure component (e.g. line 11), and posture monitoring must be maintained. The inclusion criteria (line 160) and exclusion criteria 1.2 (line 204) should not permit the inclusion of sedentary behaviour studies.

Response

Thank you for this important comment. We agree that sedentary behaviour monitoring and prolonged posture monitoring must remain conceptually distinct. We have revised the manuscript to maintain this distinction consistently throughout the **Introduction, Eligibility criteria, Results, Discussion, Abstract, and Conclusion**.

In the revised **Introduction**, prolonged posture monitoring is defined for this review as the accelerometer-based detection or reporting of uninterrupted sitting and/or standing bouts lasting at least 10 minutes during wake time.

"Given the wide range of duration thresholds, to maximise the inclusion of relevant studies, this systematic review operationally defines prolonged posture as a sitting or standing bout lasting at least 10 minutes without interruption during wake time." (lines 31-33, page 2)

We also revised the **eligibility criteria** to clarify that studies were included only when they reported or enabled interpretation of posture-specific prolonged sitting and/or standing bouts. Sedentary behaviour studies were included only if the sedentary outcome was explicitly posture-specific, for example sitting or sitting/lying derived from a posture-sensitive sensor such as activPAL, and reported as uninterrupted bouts meeting the review threshold. Studies reporting only total sedentary time, accelerometer-count sedentary time, or sedentary bouts without separating sitting/lying posture from generic low-intensity behaviour were excluded.

"The study focused on or reported the monitoring of prolonged sitting and/or standing bouts during wake time. For the purposes of this review, prolonged posture is defined as uninterrupted sitting and/or standing bouts lasting at least 10 minutes." (lines 171 – 174, page 6)

"Out-of-scope focus, including studies that monitored only bouts shorter than 10 minutes, studies of sedentary behaviour or physical activity without specified postures, posture classification studies lacking prolonged bouts, and sleep studies." (lines 193 – 195, page 6)

Major comment 2 – Evidence on health effects

Evidence on the negative health effects or relevance of prolonged posture monitoring is limited, most available evidence concerns sedentary behaviour. Please update accordingly.

Response

Thank you for this comment. We agree that the health-effect evidence should be described more cautiously. We revised the **Introduction** and **Discussion** to distinguish evidence on sedentary behaviour from evidence specifically concerning prolonged posture.

We also clarified that review focus is to synthesize how accelerometer-based wearable sensors have been used to monitor uninterrupted sitting and standing bouts:

"A comprehensive synthesis of current methodological approaches is therefore needed to guide future research." (lines 29 – 30, page 2)

"The study focused on or reported the monitoring of prolonged sitting and/or standing bouts during wake time. For the purposes of this review, prolonged posture is defined as uninterrupted sitting and/or standing bouts lasting at least 10 minutes." (lines 171 – 174, page 6)

We retained references to prolonged standing only where supported by occupational or musculoskeletal literature, and we qualified these statements as evidence of relevance rather than definitive causal evidence.

"Most of the evidence on health effects concerns total sedentary behaviour or sedentary bout accumulation rather than posture-specific monitoring. Nevertheless, bout duration and interruption patterns are increasingly recognised as relevant dimensions of sedentary exposure [5, 6]. Additionally, occupational prolonged standing has been associated with musculoskeletal symptoms and fatigue, although this evidence base is more limited and context-specific than the broader sedentary behaviour literature [7, 8]. These findings highlight the importance of monitoring uninterrupted sitting and standing bouts alongside total sedentary time." (lines 13 – 21, page 2)

These revisions better align the health rationale with the scope of the review.

Major comment 3 – Scope of included studies

The review includes only studies with substantial contributions to methodological development, including reference criteria (e.g. line 155; Figure 1). Epidemiological studies using wearables are not included. Thus, the manuscript does not review the current state of the art in monitoring, but rather advances in method development. This distinction requires clarification throughout the manuscript.

Response

Thank you for this comment. We agree that the previous wording could be interpreted as claiming to summarize all epidemiological applications of wearable sedentary behaviour monitoring. We have clarified the scope throughout the manuscript. The review is now framed as a methodological synthesis of accelerometer-based prolonged posture monitoring, rather than as a comprehensive review of all epidemiological studies using wearable sensors.

We revised the **Introduction, Objectives, Eligibility criteria, Results, Discussion, and Conclusion** to emphasize that eligible studies had to provide sufficient methodological detail on how uninterrupted sitting and/or standing bouts were monitored. Studies using wearables only to report broad sedentary behaviour outcomes, total sedentary time, or non-posture-specific activity metrics were not eligible. Therefore, the review synthesizes current methodological practices and technical approaches across the sensing-to-feedback pipeline, including data collection, data preparation, data analysis, validation, and feedback, rather than estimating the epidemiological prevalence or health effects of prolonged posture. Below provides a selection of some revisions:

"Given the wide range of duration thresholds, to maximise the inclusion of relevant studies, this systematic review operationally defines prolonged posture as a sitting or standing bout lasting at least 10 minutes without interruption during wake time. Although some studies have used shorter thresholds, such as 5 minutes [9], 10 minutes is considered more appropriate for representing prolonged posture in the context of this review. The focus is on sitting and standing, as these postures are most directly associated with occupational and daily-life musculoskeletal exposures. The focus is on sitting and standing, as these postures are most directly associated with occupational and daily-life musculoskeletal exposures." (lines 31 – 37, pages 2 – 3)

"Monitor prolonged sitting and/or standing bouts

The study focused on or reported the monitoring of prolonged sitting and/or standing bouts during wake time. For the purposes of this review, prolonged posture is defined as uninterrupted sitting and/or standing bouts lasting at least 10 minutes." (lines 170 – 174, page 6)

"Out-of-scope focus, including studies that monitored only bouts shorter than 10 minutes, studies of sedentary behaviour or physical activity without specified postures, posture classification studies lacking prolonged bouts, and sleep studies." (lines 193 – 195, page 6)

Major comment 4 – Results versus discussion

Several sections of the Results already contain interpretations, despite the presence of a separate Discussion section. Interpretative statements should be moved to the Discussion (e.g.

lines 371-376, 387-388, 397-399, 427-429, 460-462, 472ff, ...). In addition, many interpretations are not data driven and extend beyond the scope of this review (see also my detailed comments).

Response

Thank you for this comment. We agree that several parts of the Results contained interpretive statements that were better suited to the Discussion. According to the new screening process, we revised the Results and Discussion sections completely.

Specifically, we revised the Results subsections on prolonged posture characteristics, data collection, data preparation, data analysis, and user feedback. Statements interpreting the implications of heterogeneity, generalizability, sensor choice, reference criteria, and translation to feedback were either removed from the Results or moved to the corresponding Discussion subsections. For example, the Results now reports the observed variation in bout thresholds, sensor placement, reference criteria, sampling rates, preprocessing, and analytical methods, while the Discussion interprets what these variations mean for comparability, validation, and digital health implementation.

We also removed or softened statements that extended beyond the included evidence. In particular, broad conclusions about commercial devices, real-time feedback, clinical deployment, and general superiority of specific algorithm families were revised to be more cautious and explicitly tied to the included studies.

Major comment 5 – Focus of discussion

A substantial part of the Discussion appears to result from not strictly applying the inclusion and exclusion criteria (e.g. lines 738ff, 783ff), as well as from including development and refinement studies in which standardisation is not the primary objective, in contrast to epidemiological studies (e.g. line 744ff). Moreover, the Discussion introduces many additional topics and does not clearly refer back to the Introduction.

Response

Thank you for this comment. We agree and have rewritten the **Discussion**. The revised Discussion now follows the four research sub-questions introduced in the Introduction and is limited to implications arising from the included studies. We clarified that this review is a methodological synthesis of accelerometer-based prolonged posture monitoring, rather than a review of all epidemiological wearable-sensor studies. We also distinguish between different study purposes: observational and intervention studies show how prolonged sitting bout metrics have been applied, whereas validation and algorithm-development studies inform measurement and analytical performance. Material that extended beyond the review scope, including broad claims about commercial devices, clinical deployment, real-time systems, and algorithm superiority, has been removed or qualified.

Detailed comments

Abstract

How could prolonged posture monitoring go beyond general sedentary behaviour recognition, it is a part of it, no?

Response

Thank you for this comment. We agree that the wording in the **Abstract** could be misunderstood. We revised the Abstract to avoid saying that prolonged posture monitoring simply “goes beyond” sedentary behaviour recognition without explanation.

"Unlike generic sedentary behaviour monitoring based on energy expenditure, prolonged posture monitoring requires posture-specific detection and uninterrupted bout segmentation, yet current accelerometer-based approaches remain heterogeneous."

Line 3-10

Presented evidence is rather old (2016), partially questionnaire based, for sedentary behaviour (not posture monitoring), and specific for patient population (COPD). Is there more established evidence for healthy people available?

Response

Thank you for this comment. We revised the Background to use broader and more current evidence from general adult and worker populations, and we clarified that this evidence concerns sedentary behaviour and sedentary-bout accumulation rather than posture-specific prolonged posture monitoring. Specifically, we now cite WHO physical activity and sedentary behaviour guidelines, recent population-level evidence on insufficient physical activity, and an accelerometry-based systematic review of sedentary behaviour in adult workers. We also revised the wording to avoid implying that evidence on sedentary behaviour is identical to evidence on posture-specific prolonged sitting or standing.

"For health and well-being, the World Health Organisation (WHO) recommends that adults limit sedentary time and replace it with physical activity of any intensity [1]. Despite these recommendations, sedentary behaviour constitutes a substantial proportion of adult waking hours. A recent accelerometry-based systematic review found that adult workers accumulated an average of 9.22 hours per day of sedentary behaviour [2]. Globally, insufficient physical activity remains highly prevalent, with nearly 1.8 billion adults, corresponding to 31% of the global adult population, not meeting recommended levels in 2022 [3]." (lines 3-10, page 2)

[1] Bull FC, Al-Ansari SS, Biddle S, Borodulin K, Buman MP, Cardon G, et al. World Health Organization 2020 guidelines on physical activity and sedentary behaviour. British journal of sports medicine. 2020;54(24):1451-62. doi:10.1136/bjsports-2020-102955.

[2] Park S, Lee S, Woo S, Webster-Dekker K, Chen W, Veliz P, et al. Sedentary behaviors and physical activity of the working population measured by accelerometry: a systematic review and meta-analysis. BMC public health. 2024;24(1):2123. doi:10.1186/s12889-024-19449-y.

[3] Strain T, Flaxman S, Guthold R, Semenova E, Cowan M, Riley LM, et al. National, regional, and global trends in insufficient physical activity among adults from 2000 to 2022: a pooled analysis of 507 population-based surveys with 5· 7 million participants. The Lancet Global Health. 2024;12(8):e1232-43. doi:10.1016/S2214-109X(24)00150-5.

Line 20-21

“researchers are increasingly recognizing the importance of prolonged posture monitoring.” Based on?

Response

We have reframed the Introduction, and the related paragraph is now written as (lines 13-20, page 2):

"Most of the evidence on health effects concerns total sedentary behaviour or sedentary bout accumulation rather than posture-specific monitoring. Nevertheless, bout duration and interruption patterns are increasingly recognised as relevant dimensions of sedentary exposure [5, 6]. Additionally, occupational prolonged standing has been associated with musculoskeletal symptoms and fatigue, although this evidence base is more limited and context-specific than the broader sedentary behaviour literature [7, 8]. These findings highlight the importance of monitoring uninterrupted sitting and standing bouts alongside total sedentary time."

[5] Park JH, Moon JH, Kim HJ, Kong MH, Oh YH. Sedentary lifestyle: overview of updated evidence of potential health risks. Korean journal of family medicine. 2020;41(6):365. doi:10.4082/kjfm.20.0165.

[6] Honda T, Chen S, Yonemoto K, Kishimoto H, Chen T, Narazaki K, et al. Sedentary bout durations and metabolic syndrome among working adults: a prospective cohort study. BMC public health. 2016;16(1):888. doi:10.1186/s12889-016-3570-3.

[7] Waters TR, Dick RB. Evidence of health risks associated with prolonged standing at work and intervention effectiveness. Rehabilitation Nursing. 2015;40(3):148-65. doi:10.1002/rnj.166.

[8] Coenen P, Parry S, Willenberg L, Shi JW, Romero L, Blackwood DM, et al. Associations of prolonged standing with musculoskeletal symptoms—A systematic review of laboratory studies. Gait Posture. 2017;58:310-8. doi:10.1016/j.gaitpost.2017.08.024.

Line 23-25

Please clarify “temporal continuity rather than intensity variation.”

Response

Thank you for this comment. The sentence has been removed. The **Introduction** has been restructured to clarify the distinction directly: sedentary behaviour is defined according to SBRN by low energy expenditure while sitting, reclining, or lying, whereas prolonged posture monitoring in this review refers to uninterrupted sitting or standing bouts during wake time.

"The Sedentary Behaviour Research Network defines sedentary behaviour as any waking behaviour characterized by an energy expenditure $\leqslant$1.5 metabolic equivalents while in a sitting, reclining, or lying posture [4]." (lines 11-13, page 2)

[4] Tremblay MS, Aubert S, Barnes JD, Saunders TJ, Carson V, Latimer-Cheung AE, et al. Sedentary behavior research network (SBRN)–terminology consensus project process and outcome.

International journal of behavioral nutrition and physical activity. 2017;14(1):75. doi:10.1186/s12966-017-0525-8.

"Given the wide range of duration thresholds, to maximize the inclusion of relevant studies, this systematic review operationally defines prolonged posture as a sitting or standing bout lasting at least 10 minutes without interruption during wake time." (lines 31-33, page 2)

Line 31-32

This statement might need a reference.

Response

This statement has been revised during the restructuring of the **Introduction** (lines 37-39, page 3):

"Prolonged lying or reclining postures during sleep are excluded, since distinguishing in-bed sedentary behaviour from sleep presents distinct physiological and methodological challenges [16]."

[16] Barone Gibbs B, Kline CE. When does sedentary behavior become sleep? A proposed framework for classifying activity during sleep-wake transitions. International Journal of Behavioral Nutrition and Physical Activity. 2018;15(1):81. doi:10.1186/s12966-018-0712-2.

Line 52-52

Studies vary widely in feedback strategies, but only 2 were included, right?

Response

We agree that the previous wording overemphasized feedback strategies and was not aligned with the primary monitoring focus of the review. We have removed the statement about variability in feedback strategies from this part of the Introduction. Feedback is now addressed only where directly relevant, in the Results and Discussion sections on user feedback.

The revised text now focuses on methodological heterogeneity in monitoring approaches (lines 52 – 55, page 3):

"Despite these technological developments, current approaches remain methodologically diverse. As noted previously, studies differ in sensor selection, placement, wear protocols, data preparation, and analysis methods for quantifying prolonged posture."

Line 89-90

“few have examined how methodological decisions across these stages influence real-world deployment or clinical translational potential.” Is there any study who has investigated this topic?

Response

We have revised this statement (lines 85-88, page 4):

"Notably, to our best knowledge, none of the previous reviews has synthesised the full sensing-to-feedback pipeline, from data collection and preparation to analytical methods and, where applicable, feedback-related features."

Line 144

What standardized SBRN terminology device names were used?

Response

We have included the used SBRN terminologies and rephrased the corresponding section (lines 143 – 149, page 5):

"To enhance search sensitivity, during the update search in December 2025, we refined the search strategy. Specifically, we included behaviour and posture-related terminology defined by SBRN (“sedentary bout” and “standing bout”) and terms commonly used in posture monitoring literature (e.g., “sitting bout”, “uninterrupted sitting”, “sit to stand”) [4, 9, 26]. Furthermore, we identified device-specific terms from the wearable sensor literature and preliminary search results, such as “activPAL”, “ActiGraph”, “Axivity”, “GENEActiv”, “Movesense”, and “movisens”."

Line 163

What are insights into? A typical epidemiological study mentions placement and how they process data, but they don’t have a reference criteria. Please elaborate.

Response

Thank you for this comment. We agree that the previous wording was unclear. The phrase “provide insights into” has been removed. We revised the inclusion criteria to state explicitly what methodological information was required for eligibility. A study did not need to include an independent reference criterion unless it was a validation or algorithm-development study. However, all included studies had to provide enough information to determine how prolonged sitting and/or standing bouts were monitored.

Specifically, eligible studies were required to report, at minimum, the population, sensor type and anatomical placement, measurement protocol, and the method used to derive prolonged-posture outcomes. We also clarified that epidemiological or observational studies will only be excluded if they reported total sedentary time or generic sedentary bouts without posture-specific sitting or standing information.

"The study focused on or reported the monitoring of prolonged sitting and/or standing bouts during wake time." (Inclusion criteria item 1, lines 171-172, page 6)

"The study provided adequate detail to allow interpretation of the prolonged posture monitoring, which, at a minimum, includes: (i) population; (ii) sensor type and anatomical placement; (iii) measurement protocol, such as wear duration and tasks; and (iv) methods used to derive prolonged posture outcomes.." (Inclusion criteria item 3, lines 181-184)

"Out-of-scope focus, including studies that monitored only bouts shorter than 10 minutes, studies of sedentary behaviour or physical activity without specified postures, posture classification studies lacking prolonged bouts, and sleep studies." (Exclusion criteria item 1, lines 191-193)

Line 197 to 228

There is no need to repeat the opposite of the inclusion criteria as exclusion criteria.

Response

We agree and have simplified the exclusion criteria to avoid repeating the inverse of the inclusion criteria. The revised exclusion criteria now list only the main categories of studies that were excluded to clarify the scope of the review with representative examples of exclusion.

Exclusion criteria (lines 190 – 201)

Studies that did not meet all the inclusion criteria were excluded. The following are examples of exclusion:

1. Out-of-scope focus, including studies that monitored only bouts shorter than 10 minutes, studies of sedentary behaviour or physical activity without specified postures, posture classification studies lacking prolonged bouts, and sleep studies.
2. Ineligible instrumentation, defined as posture detection relying on non-wearable or non-accelerometer-based methods.
3. Insufficient methodological detail on how the prolonged postures were monitored.
4. Ineligible population or publication, including non-human studies, non-English full texts, inaccessible full texts, and publications that were not peer-reviewed or otherwise did not meet eligibility requirements.

Line 248

I suggest to reference the excluded studies (full-text stage) in the manuscript or an appendix file for the interested reader.

Response

We have provided an extensive list of the excluded studies in the full-text state with primary reason for exclusion in **S4 File**. This is also mentioned in the manuscript (lines 289-290):

"A comprehensive list of excluded studies, along with the rationale for exclusion, is available in S4 File."

Line 257

I suggest to focus on tools used and not present tools not used.

Response

We agree and have revised the **risk-of-bias methods** to focus only on the appraisal tools that were actually used. The revised text now states that design-specific tools were selected according to study type: WEAR-BOT for wearable-sensor validation or algorithm-development studies, the JBI checklist for analytical cross-sectional studies, and the JBI checklist for randomized controlled trials. (lines 243-248, pages 7-8)

"Given the heterogeneity of study designs, quality and risk of bias were assessed with design-specific tools. Method development and validation studies were evaluated using the WEArable Technology Risk of Bias and Objectivity Tool (WEAR-BOT) validity checklist [27]. Observational studies were appraised with the Joanna Briggs Institute (JBI) checklist for analytical cross-sectional studies [28], and intervention studies were assessed with the JBI critical appraisal checklist for randomised controlled trials [29]."

[27] Carrier B, Bunn JA, Eschbach C, Reece JD, Welk GJ, Dolezal BA, et al. The WEAR-BOT checklist: A risk of bias tool for evaluating validity and reliability research in wearable technology. PLOS ONE. 2026 02;21(2):1-23. doi:10.1371/journal.pone.0338014.

[28] Moola S, Munn Z, Tufanaru C, Aromataris E, Sears K, Sfetcu R, et al. Chapter 7: Systematic reviews of etiology and risk. In: Aromataris E, Munn Z, editors. JBI Manual for Evidence Synthesis. JBI; 2020. Available from https://synthesismanual.jbi.global. Available from: https://synthesismanual.jbi.global.

[29] Barker TH, Stone JC, Sears K, Klugar M, Tufanaru C, Leonardi-Bee J, et al. The revised JBI critical appraisal tool for the assessment of risk of bias for randomized controlled trials. JBI evidence synthesis. 2023;21(3):494-506. doi:10.11124/JBIES-22-00430.

Figure 3

This is confusing. Please include reference numbers of the manuscript, not the appendix. Same for fig 4

Response

Thank you for this comment. We revised the figures to avoid appendix-only numbering. The original Figure 3 has been moved to **S6 File** due to space and readability constraints. Both the supplementary version and the figure retained in the main manuscript now include first author names and manuscript reference numbers. The visual labels use the data extraction index numbers (from 1 to 14) only to maintain figure clarity, and an index-to-reference mapping is provided within the **Figure 4** (the current Figure 3):

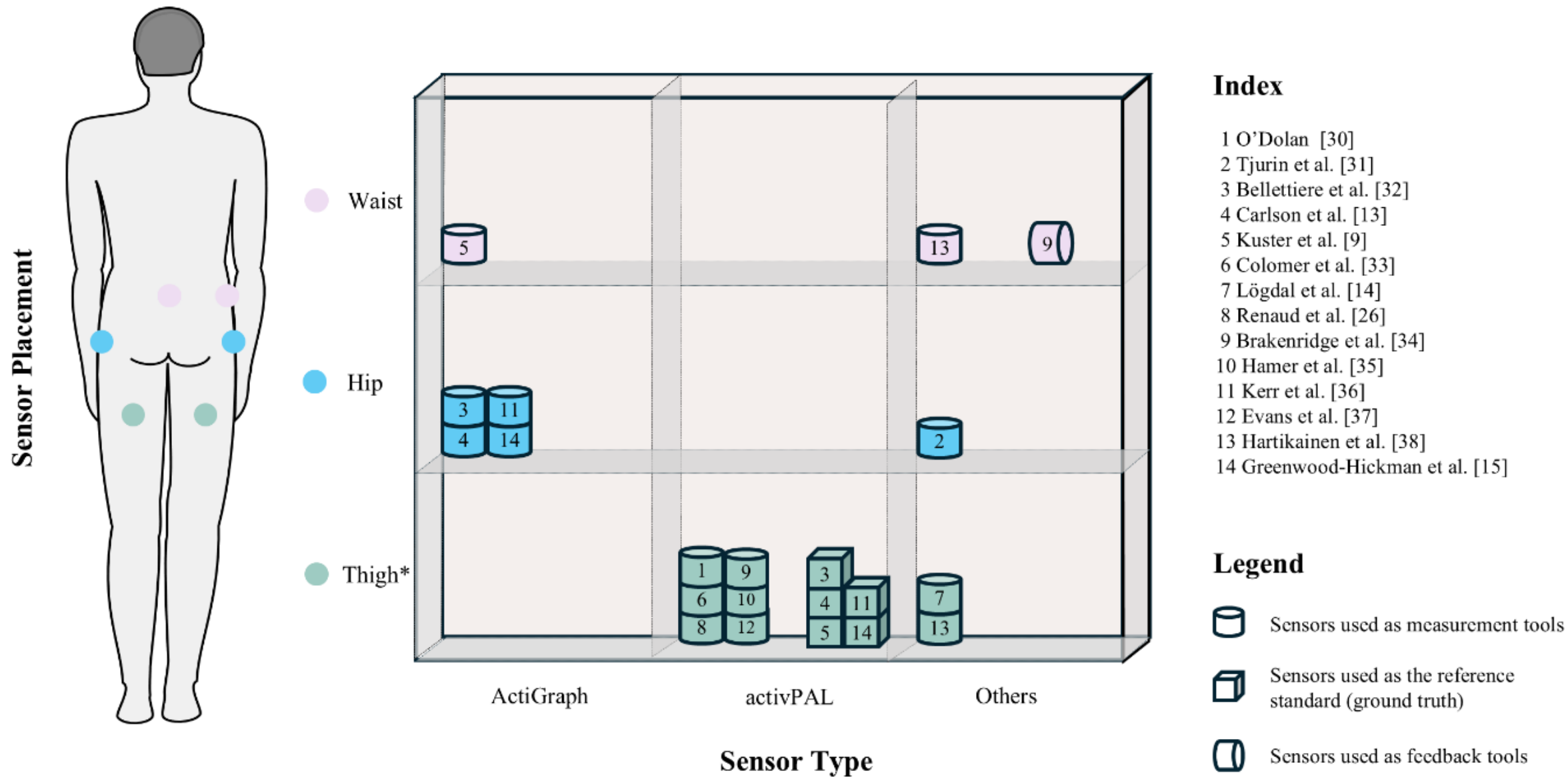

Fig 3. Distribution of studies by sensor placement and sensor type. The index corresponds to the index presented in the data extraction form in S5 File. Numbers in brackets following author names indicate reference numbers. In the visualisation, sensor placement is indicated by colour, sensor type by layering, and sensor role by shape. The legend specifies that vertical cylinders represent sensors used as measurement tools, cubes indicate sensors used as reference standards (ground truth), and horizontal cylinders represent sensors used as feedback tools. Thigh-mounted sensors are located at the midline of the anterior aspect of the thigh.*

Line 339

activPAL is not an inclinometer.

Response

We have revised the **Results** section completely, and this is no longer applicable.

Line 341-342

Again, sedentary behaviour is not part of this review, please exclude.

Response

Thank you for this comment. This issue is no longer applicable in the revised manuscript. We have revised the **Results** and **eligibility criteria** to maintain a clear distinction between sedentary behaviour and posture-specific prolonged sitting/standing monitoring. Studies or outcomes reporting sedentary behaviour without posture-specific sitting or standing bout information were excluded from the review and are no longer discussed as part of the included evidence. Where the term "sedentary"' remains, it is used only when it reflects the terminology of an included study and refers to posture-derived sitting or sitting/lying bouts measured with a posture-sensitive device. The text has been revised to avoid presenting generic sedentary behaviour monitoring as part of the review scope.

Line 359

It is unclear what the authors mean with “As demonstrated in Fig 4, sensor placement varied according to study objectives, device design, and validation strategies.” There is no such information on objectives, design, and validation included in figure 4

Response

We agree that the previous wording was not accurate. We have revised the sentence to describe only what is presented in the figure:

"Fig 3 presents the distribution of studies by sensor placement and sensor type." (lines 447 – 448, page 12)

Line 366

Pairing it by Bluetooth?

Response

This issue is no longer applicable in the revised manuscript.

Line 368-370

The added value of this sentence is unclear, no association is presented here.

Response

This issue is no longer applicable in the revised manuscript.

Entire result section

When talking about the single studies, always reference them for transparency, e.g. line 386-387.

Response

Thank you for the comment, we have revised the manuscript to always ensure there is reference.

Line 436-437

these studies should probably not have been included, should they?

Response

Thank you for this comment. In response to this and related comments, we revised the inclusion and exclusion criteria to more strictly separate posture-specific prolonged sitting/standing monitoring from generic sedentary-behaviour monitoring. We then re-screened all full-text articles against the final eligibility criteria.

As a result, studies were retained only if they reported or enabled interpretation of uninterrupted sitting and/or standing bouts of at least 10 minutes during wake time using body-worn

accelerometer-based sensors. Studies reporting only total sedentary time, generic sedentary bouts, or activity-classification outcomes without posture-specific prolonged sitting or standing bout information were excluded. The **Results**, **PRISMA flow diagram**, and **in/excluded-study supplement (S4 File, S5 File)** have been updated accordingly.

Line 451

The sampling rate is not a part of preprocessing

Response

Thank you for the comment, we have revised the manuscript and presented sampling rate in the **data collection** stage instead. (lines 509-519, page 14)

Line 456

Please show the evidence why sampling rate is crucial

Response

We added references and revised the text to clarify why sampling rate matters in the **discussion** (lines 1006-1012):

"Sampling rate should be treated as a deliberate design choice rather than a technical detail. Sampling rates affect the processing and comparability of raw accelerometer data, including activity-count generation and cross-device comparability [62, 63]. It also influences the trade-off between signal resolution, classification performance, data volume, and battery consumption. Lower sampling rates can reduce storage and power demands, but the acceptable rate depends on the target behaviour, sensor placement, and analytical model [64, 65]." "Sampling rate should be treated as a deliberate design choice rather than a technical detail. Sampling rates affect the processing and comparability of raw accelerometer data, including activity-count generation and cross-device comparability [62, 63]. It also influences the trade-off between signal resolution, classification performance, data volume, and battery consumption. Lower sampling rates can reduce storage and power demands, but the acceptable rate depends on the target behaviour, sensor placement, and analytical model [64, 65]."

[62] Brønd JC, Arvidsson D. Sampling frequency affects the processing of Actigraph raw acceleration data to activity counts. Journal of applied physiology. 2016;120(3):362-9. doi:10.1152/japplphysiol.00628.2015.

[63] Lettink A, van Wieringen WN, Altenburg TM, Chinapaw MJM, van Hees VT. Inter-Brand, -Dynamic Range, and -Sampling Rate Comparability of Raw Accelerometer Data as Used in Physical Behavior Research. Journal for the Measurement of Physical Behaviour. 2024;7(1):jmpb.2024-0003. doi:10.1123/jmpb.2024-0003.

[64] Small S, Khalid S, Dhiman P, Chan S, Jackson D, Doherty A, et al. Impact of Reduced Sampling Rate on Accelerometer-Based Physical Activity Monitoring and Machine Learning Activity Classification. Journal for the Measurement of Physical Behaviour. 2021;4(4):298 310. doi:10.1123/jmpb.2020-0061.

[65] Yamane T, Kimura M, Morita M. Effects of sampling frequency on human activity recognition with machine learning aiming at clinical applications. Sensors. 2025;25(12):3780. doi:10.3390/s25123780.

Line 468

How dynamic are these posture transitions that they need 50 to 100 Hz?

Response

Thank you for this comment. This issue is no longer applicable in the revised manuscript. We removed the statement implying that posture transitions require 50-100 Hz sampling. (lines 510 – 519, page 14)

Line 481

Common but no study included used it?

Response

This issue is no longer applicable in the revised manuscript. The entire **Results** of stage 2 (lines 520 – 720, pages 14-18) has been restructured, and the revised text now focuses only on methods used in the included studies.

Line 487

Are denoising and filtering considered independent topics or not? This is unclear.

Response

This issue is no longer applicable in the revised manuscript. Based on the characteristics of the latest included studies, we restructured the Stage 2 and renamed it as Data preparation, then divided it into 4 subsections: signal extraction and preprocessing, wear-time, sleep, and invalid-data handling, segmentation and epoching, and feature extraction. This topic is now under the subsection 'signal extraction and preprocessing' (lines 530-562, pages 14-15).

Line 758-761

Is there evidence for the fact that adherence to wear and ActiGraph is higher than wearing an activPAL?

Response

This issue is no longer applicable in the revised manuscript.

Line 768

this is not needed for the activPAL, why should it be needed for other sensors?

Response

This issue is no longer applicable in the revised manuscript.

Line 775

this review is about posture detection, not about asymmetry, and micro-adjustments... see also 805 or 825

Response

We have removed all the irrelevant **discussions** and focus solely on prolonged posture monitoring.

Line 777

Is it needed to repeat this information, it is also available from sensor manufacturers?

Response

We have completely rewritten the **discussion** and this issue is no longer applicable in the revised manuscript.

Line 783

The review is about development studies, what is the expectation? (A major methodological challenge is the lack of standardization in sensor placement)

Response

Thank you for this comment. We agree that the previous statement was too broad and did not sufficiently account for the purpose of method development studies. Based on the new results, we have revised the Discussion completely. The relevant part is now written as:

"In summary, data collection choices in prolonged posture monitoring are design decisions that influence validity, usability, and generalisability throughout the research process. Researchers should select and report sensor configurations that align with the monitoring objective. Whether wrist-worn devices, which impose a lower user burden and are widely used in consumer wearables, can support prolonged posture monitoring remains an open question, as no included study evaluated this configuration. Practitioners should assess whether the device and placement are appropriate for the user population and the intended clinical or ergonomic application before implementing prolonged posture metrics in practice." (lines 1013-1021)

Line 791

Is there an ask for self-reported reference criteria here?

Response

This issue is no longer applicable in the revised **discussion**.

Line 793

not part of this review topic.

Response

We have revised the **Discussion** thoroughly and removed all the irrelevant topics in the discussion.

Line 808

the focus of this review is on prolonged postures...

Response

We have revised the sentences and focus on prolonged postures.

Line 813

the mix of intermediate and outcome features is somewhat confusing. It would help if these topics are presented separately.

Response

The **Discussion** section has been revised completely and this issue is no longer applicable. The Discussion on features is now written as:

"Feature extraction decisions affect both analytical performance and interpretability. Manually engineered features offer transparency and may generalise well across some activity-recognition domains, but they require domain expertise and careful feature design [47]. Proprietary software outputs are convenient, but they can limit transparency and reproducibility when the underlying algorithms are inaccessible or updated without full methodological disclosure [71]. Deep learning models can learn features directly from raw wearable-sensor signals and have advanced human activity recognition, but they typically require large labelled datasets and careful validation [72]. The choice of feature extraction method should therefore align with the specific application, for example, transparency is crucial for clinical interpretation, while higher model complexity can be justified for predictive tasks when supported by validated data and appropriate interpretability methods [73, 74].

[47] Bento N, Rebelo J, Barandas M, Carreiro AV, Campagner A, Cabitza F, et al. Comparing handcrafted features and deep neural representations for domain generalization in human activity recognition. Sensors. 2022;22(19):7324. doi:10.3390/s22197324.

[71] Giurgiu M, von Haaren-Mack B, Fiedler J, Woll S, Burchartz A, Kolb S, et al. The wearable landscape: Issues pertaining to the validation of the measurement of 24-h physical activity, sedentary, and sleep behavior assessment. Journal of Sport and Health Science. 2025;14:101006. doi:10.1016/j.jshs.2024.101006.

[72] Zhang S, Li Y, Zhang S, Shahabi F, Xia S, Deng Y, et al. Deep learning in human activity recognition with wearable sensors: A review on advances. Sensors. 2022;22(4):1476. doi:10.3390/s22041476.

[73] Ahmad MA, Eckert C, Teredesai A. Interpretable Machine Learning in Healthcare. In: Proceedings of the 2018 ACM International Conference on Bioinformatics, Computational Biology, and Health Informatics. BCB '18. New York, NY, USA: Association for Computing Machinery; 2018. p. 559–560. doi:10.1145/3233547.3233667.

[74] Linardatos P, Papastefanopoulos V, Kotsiantis S. Explainable ai: A review of machine learning interpretability methods. Entropy. 2020;23(1):18. doi:10.3390/e23010018.

Line 832

ML is only the default for development studies, epidemiological studies still most often use cut-points, no?

Response

We have revised the related section and it now reads as:

"The reviewed studies employed analytical methods for two primary purposes: applying posture metrics as epidemiological or intervention outcomes, and developing algorithms for posture classification and bout estimation. This distinction is important because the required level of accuracy depends on the intended use." (lines 1074 – 1077, page 27)

Line 836

performance is no review topic, is it?

Response

We have revised the **Discussion** section completely and this issue is no longer applicable.

Line 862

Why should the reviewed field (studies with reference criteria) implement feedback, a different field might need to be reviewed regarding this topic, no?

Response

Thank you for your comment. We agree that validation and method-development studies are intended to establish measurement or algorithmic performance, and should not be expected to implement feedback. We have revised the **Discussion** to clarify this and to avoid suggesting that feedback is required in all included studies.

In the revised manuscript, we discuss user feedback as a translational stage in the sensing-to-feedback pipeline, not as a criterion for methodological quality. We note that prolonged posture monitoring can serve epidemiological or evaluative purposes without user feedback. However, a

key digital health goal is to translate monitoring outputs into timely, actionable support for behaviour change. This approach aligns with just-in-time adaptive intervention frameworks, which focus on delivering appropriate support based on a person's changing state. Accordingly, we present feedback as a future digital health application and a current gap in translating monitoring into intervention, rather than as an expectation for validation studies:

"Prolonged posture monitoring may serve epidemiological or evaluative goals without providing user feedback. On the other hand, as defined by WHO, translating the latest data, research, and evidence into action is a key objective for advancing digital health and innovation [79]. Therefore, this review examines how studies converted monitoring outputs into timely, actionable support for behaviour change. This approach aligns with just-in-time adaptive intervention frameworks, which focus on delivering appropriate support at the right moment based on an individual's changing state [77]. However, none of the reviewed studies progressed from validated posture classification to adaptive, individualized feedback. Instead, the four intervention studies used fixed timers or reminders rather than real-time detection. This feedback gap likely results from unresolved measurement challenges in Stages 1 to 3." (lines 1122-1132, page 28)

[77] Nahum-Shani I, Smith SN, Spring BJ, Collins LM, Witkiewitz K, Tewari A, et al. Just-in-time adaptive interventions (JITAIs) in mobile health: key components and design principles for ongoing health behavior support. Annals of behavioral medicine. 2016:1-17. doi:10.1007/s12160-016-9830-8.

[79] World Health Organization. Digital Health; 2026. Accessed: 2026-05-01. https://www.who.int/health-topics/digital-health#tab=tab_2.

Future works

most recommendations are not based on the results of this review, please revise.

Response

We have revised the **Future works** section (page 29) completely to make the suggestions based on our results.

"Future research should prioritize the following directions, in line with the four-stage sensing-to-feedback pipeline:

1 Prolonged standing should be treated as a distinct monitoring target. Research should include dedicated validation in standing-intensive occupations and report bout-level standing outcomes alongside sitting metrics.

2 Greater consistency is needed in the operational definition of "prolonged", including explicit justification of bout duration, interruption rules, tolerance, and wake time definitions.

3 Validation reporting should include separate performance metrics for posture classification, transition detection, total time, bout duration, and prolonged bout accumulation, in addition to overall classification accuracy. It should also incorporate equivalence testing, Bland–Altman limits of agreement, and pre-specified validity thresholds.

4 It is necessary to evaluate whether algorithms validated in post-hoc analyses can operate with sufficient latency and reliability for real-time bout detection and closed-loop feedback.

5 The evidence base should be expanded beyond Western, office-based, adult populations to include children, older adults, clinical groups, standing-intensive workers, and non-Western settings.

6 Data preparation pipelines should be reported in sufficient detail to ensure reproducibility. This includes software, parameters, synchronization, sleep and non-wear handling, epoching, and bout-construction rules, supporting cross-study comparison and the transition to scalable digital health systems."

Conclusion

most conclusions are not supported by the reviewed studies, please revise.

Response

We have revised the **Conclusion** to ensure it is based on the reviewed studies.

"This systematic review demonstrates the technical feasibility of accelerometer-based monitoring of prolonged sitting, particularly when validated algorithms are applied to thigh or hip-worn sensors. Nevertheless, the field remains heterogeneous, with considerable variation in definitions of “prolonged”, inconsistent data preparation and analytical methods, and limited progress in translating post-hoc classification into real-time feedback. Prolonged standing has received little attention as a distinct monitoring target, and the current evidence base is largely derived from Western, office-based, adult populations. Overcoming these limitations will require comprehensive technical reporting, inclusive validation, interpretable analytics, and sustained interdisciplinary collaboration to transition prolonged posture monitoring from a technically feasible research tool to an evidence-based, scalable digital health solution."

Line 955

are you asking for standardized calibration study designs?

Response

This issue is no longer applicable in the revised **conclusion**. The relevant sentence (lines 1217 – 1220) now reads as:

"Overcoming these limitations will require comprehensive technical reporting, inclusive validation, interpretable analytics, and sustained interdisciplinary collaboration to transition prolonged posture monitoring from a technically feasible research tool to an evidence-based, scalable digital health solution."

Journal Requirements

Comment 1

Please amend your detailed Financial Disclosure statement. This is published with the article. It must therefore be completed in full sentences and contain the exact wording you wish to be published.

I. Please clarify all sources of financial support for your study. List the grants, grant numbers, and organizations that funded your study, including funding received from your institution. Please note that suppliers of material support, including research materials, should be recognized in the Acknowledgements section rather than in the Financial Disclosure.
II. State the initials, alongside each funding source, of each author to receive each grant. For example: "This work was supported by the National Institutes of Health (####### to AM; ###### to CJ) and the National Science Foundation (###### to AM)."
III. State what role the funders took in the study. If the funders had no role in your study, please state: "The funders had no role in study design, data collection and analysis, decision to publish, or preparation of the manuscript."
IV. If any authors received a salary from any of your funders, please state which authors and which funders.

Response

Thank you for your reminder. We have updated the **financial disclosure statement** in the manuscript to include the funding sources, grant numbers, and recipient author initials in complete sentences for publication. We have also clarified the funders' roles and stated that no author received a salary from them.

"This research was supported by the China Scholarship Council (No.202208090040 to M.L. and No.202406020168 to K.H.) and the Academic Excellence Foundation of BUAA for PhD Students (to K.H.). These funding sources provided only scholarship support. The funders had no role in study design, data collection and analysis, decision to publish, or preparation of the manuscript. No author received a salary from any of these funders."

Comment 2

We ask that a manuscript source file is provided at Revision. Please upload your manuscript file as a .doc, .docx, .rtf or .tex.

Response

We have prepared the manuscript file in .tex format.

Comment 3

Please upload separate figure files in .tif or .eps format. Also, remove the figures from your manuscript file but keep the legends.

For more information about figure files please see our guidelines:

https://journals.plos.org/digitalhealth/s/figures

https://journals.plos.org/digitalhealth/s/figures#loc-file-requirements

Response

We have prepared the figures in .tif format and made sure they meet the journal's requirements.

Comment 4

As required by our policy on Data Availability, please ensure your manuscript or supplementary information includes the following:

- A numbered table of all studies identified in the literature search, including those that were excluded from the analyses.
- For every excluded study, the table should list the reason(s) for exclusion.
- If any of the included studies are unpublished, include a link (URL) to the primary source or detailed information about how the content can be accessed.
- A table of all data extracted from the primary research sources for the systematic review and/or meta-analysis. The table must include the following information for each study:
    - Name of data extractors and date of data extraction
    - Confirmation that the study was eligible to be included in the review.
    - All data extracted from each study for the reported systematic review and/or meta-analysis that would be needed to replicate your analyses.
- If data or supporting information were obtained from another source (e.g. correspondence with the author of the original research article), please provide the source of data and dates on which the data/information were obtained by your research group.
- If applicable for your analysis, a table showing the completed risk of bias and quality/certainty assessments for each study or outcome. Please ensure this is provided for each domain or parameter assessed. For example, if you used the Cochrane risk-of-bias tool for randomized trials, provide answers to each of the signalling questions for each study. If you used GRADE to assess certainty of evidence, provide judgements about each of the quality of evidence factor. This should be provided for each outcome.
- An explanation of how missing data were handled.

This information can be included in the main text, supplementary information, or relevant data repository. Please note that providing these underlying data is a requirement for publication in this journal, and if these data are not provided your manuscript might be rejected.

Response

We have prepared extensive supporting information files to meet the journal requirement, the files include:

- The PRISMA checklist (S1 File)
- The latest version of PROSPERO registration (S2 File)
- Full search strategies for all databases in all rounds (S3 File)

- The list of excluded studies in full-text screening with primary reasons of exclusion (S4 File)
- The complete data extraction form of included studies (S5 File)
- An visual overview of study characteristics (S6 File)
- Risk of bias assessment operational rubrics and per-study per-item judgment of both reviewers (S7 File)

Comment 5

In the online submission form, you indicated that "This systematic review is based on data extracted from previously published articles. All sources are cited within the manuscript. A subset of the extracted data is provided in the supporting information, and the entire data extraction form compiled for this systematic review is available upon request from the corresponding authors.".

All PLOS journals now require all data underlying the findings described in their manuscript to be freely available to other researchers, either

1. In a public repository,
2. Within the manuscript itself, or
3. Uploaded as supplementary information.

This policy applies to all data except where public deposition would breach compliance with the protocol approved by your research ethics board. If your data cannot be made publicly available for ethical or legal reasons (e.g., public availability would compromise patient privacy), please explain your reasons by return email and your exemption request will be escalated to the editor for approval. Your exemption request will be handled independently and will not hold up the peer review process, but will need to be resolved should your manuscript be accepted for publication. One of the Editorial team will then be in touch if there are any issues.

Response

We have updated our manuscripts and all data are now provided in supporting information:

S1 File. PRISMA checklist.

S2 File. PROSPERO registration. Study protocol registered in PROSPERO (CRD42025637387), last updated on April 18, 2026 (Version 5.0).

S3 File. Full search strategies for all databases. This supplement presents the full search strategies used for each database, including Web of Science, Scopus, ProQuest Central, PubMed, Embase, CINAHL, SPORTDiscus, IEEE Xplore, ACM Digital Library, and Engineering Village (EI). It provides the detailed strategies for the initial search (January 12, 2025), the updated search (December 4, 2025), and the catch-up search (April 8, 2026), the number of records retrieved from each database before deduplication, and the modifications in each search.

S4 File. Excluded studies. List of studies excluded during full-text screening, with primary reasons for exclusion.

S5 File. Data extraction. Complete data extraction for all 14 included studies.

S6 File. Visual overview of study characteristics. This supplement provides a visual summary of the methodological characteristics of the 14 included studies across the sensing-to-feedback pipeline. Key aspects covered include data collection, data preparation, data analysis, user feedback, reproducibility-related reporting items, and study category.

S7 File. Risk of bias assessment operational rubrics and per-study judgments. This supplement provides the operational rubrics applied in the risk-of-bias assessment, and the per-item judgments made by both reviewers for each study.

Comment 6

Some material included in your submission may be copyrighted. According to PLOS's copyright policy, authors who use figures or other material (e.g., graphics, clipart, maps) from another author or copyright holder must demonstrate or obtain permission to publish this material under the Creative Commons Attribution 4.0 International (CC BY 4.0) License used by PLOS journals. Please closely review the details of PLOS's copyright requirements here: PLOS Licenses and Copyright. If you need to request permissions from a copyright holder, you may use PLOS's Copyright Content Permission form.

Please respond directly to this email or email the journal office and provide any known details concerning your material's license terms and permissions required for reuse, even if you have not yet obtained copyright permissions or are unsure of your material's copyright compatibility.

Potential Copyright Issues:

a. Figures 1 and 4: Please confirm whether you drew the images / clip-art within the figure panels by hand. If you did not draw the images, please provide (a) a link to the source of the images or icons and their license / terms of use; or (b) written permission from the copyright holder to publish the images or icons under our CC-BY 4.0 license. Alternatively, you may replace the images with open source alternatives. See these open source resources you may use to replace images / clip-art:
   - https://commons.wikimedia.org
   - https://openclipart.org/

Response

Thank you for the remarks. We have replaced the icons with open-source alternatives. The links of these icons are as follows:

Fig 1:

- Icon for stage 1 (data collection): https://openclipart.org/detail/100999/analog-to-digital
- Icon for stage 2 (data preparation): https://openclipart.org/detail/305652/data-filter
- Icon for stage 3 (data analysis): https://openclipart.org/detail/200086/primary-knetload
- Icon for stage 4 (user feedback): https://openclipart.org/detail/279779/button-contact-us

Male body figure used in Fig 3 (previously Fig 4): https://openclipart.org/detail/14572/male-body-silhouette-back

Closing

We thank the Editors and Reviewers for your thoughtful and constructive feedback. We believe that our revisions have largely enhanced the clarity, methodological transparency, and practical relevance of the manuscript. Specifically, the revised version more clearly defines the scope of prolonged posture monitoring, avoids overgeneralization from heterogeneous evidence, and offers more actionable guidance for future accelerometer-based monitoring and digital health research. We hope that the revised manuscript now meets your expectations.

Sincerely,
Meixing Liao (on behalf of all co-authors)
