# Supplementary material for: A systematic review of prolonged posture monitoring with accelerometer-based wearable sensors
Source: PLOS Digit Health. 2026 Sep 15;5(9):e0001538. doi: 10.1371/journal.pdig.0001538 (PMC13577576; doi:10.1371/journal.pdig.0001538)
Supplement: Peer review history

# Peer review: Decision Letter 0

**DOI:** 10.1371/journal.pdig.0001538.r001

**Authors:** Nicole Li-Jessen, Josephine Nanyonjo

5 Mar 2026

Response to Reviewers Revised Manuscript with Track Changes Manuscript **Journal Requirements:**

1. Please amend your detailed Financial Disclosure statement. This is published with the article. It must therefore be completed in full sentences and contain the exact wording you wish to be published.

i. Please clarify all sources of financial support for your study. List the grants, grant numbers, and organizations that funded your study, including funding received from your institution. Please note that suppliers of material support, including research materials, should be recognized in the Acknowledgements section rather than in the Financial Disclosure.

ii. State the initials, alongside each funding source, of each author to receive each grant. For example: "This work was supported by the National Institutes of Health (####### to AM; ###### to CJ) and the National Science Foundation (###### to AM)."

iii. State what role the funders took in the study. If the funders had no role in your study, please state: “The funders had no role in study design, data collection and analysis, decision to publish, or preparation of the manuscript.”

iv. If any authors received a salary from any of your funders, please state which authors and which funders.

2. We ask that a manuscript source file is provided at Revision. Please upload your manuscript file as a .doc, .docx, .rtf or .tex.

3. Please upload separate figure files in .tif or .eps format. Also, remove the figures from your manuscript file but keep the legends.

For more information about figure files please see our guidelines:

https://journals.plos.org/digitalhealth/s/figures

https://journals.plos.org/digitalhealth/s/figures#loc-file-requirements

4. As required by our policy on Data Availability, please ensure your manuscript or supplementary information includes the following:

A numbered table of all studies identified in the literature search, including those that were excluded from the analyses.

For every excluded study, the table should list the reason(s) for exclusion.

If any of the included studies are unpublished, include a link (URL) to the primary source or detailed information about how the content can be accessed.

A table of all data extracted from the primary research sources for the systematic review and/or meta-analysis. The table must include the following information for each study:

Name of data extractors and date of data extraction

Confirmation that the study was eligible to be included in the review.

All data extracted from each study for the reported systematic review and/or meta-analysis that would be needed to replicate your analyses.

If data or supporting information were obtained from another source (e.g. correspondence with the author of the original research article), please provide the source of data and dates on which the data/information were obtained by your research group.

If applicable for your analysis, a table showing the completed risk of bias and quality/certainty assessments for each study or outcome.  Please ensure this is provided for each domain or parameter assessed. For example, if you used the Cochrane risk-of-bias tool for randomized trials, provide answers to each of the signalling questions for each study. If you used GRADE to assess certainty of evidence, provide judgements about each of the quality of evidence factor. This should be provided for each outcome.

An explanation of how missing data were handled.

This information can be included in the main text, supplementary information, or relevant data repository. Please note that providing these underlying data is a requirement for publication in this journal, and if these data are not provided your manuscript might be rejected.

5. In the online submission form, you indicated that “This systematic review is based on data extracted from previously published articles. All sources are cited within the manuscript. A subset of the extracted data is provided in the supporting information, and the entire data extraction form compiled for this systematic review is available upon request from the corresponding authors.”.

All PLOS journals now require all data underlying the findings described in their manuscript to be freely available to other researchers, either

1. In a public repository,

2. Within the manuscript itself, or

3. Uploaded as supplementary information.

This policy applies to all data except where public deposition would breach compliance with the protocol approved by your research ethics board. If your data cannot be made publicly available for ethical or legal reasons (e.g., public availability would compromise patient privacy), please explain your reasons by return email and your exemption request will be escalated to the editor for approval. Your exemption request will be handled independently and will not hold up the peer review process, but will need to be resolved should your manuscript be accepted for publication. One of the Editorial team will then be in touch if there are any issues.

6. Some material included in your submission may be copyrighted. According to PLOS’s copyright policy, authors who use figures or other material (e.g., graphics, clipart, maps) from another author or copyright holder must demonstrate or obtain permission to publish this material under the Creative Commons Attribution 4.0 International (CC BY 4.0) License used by PLOS journals. Please closely review the details of PLOS’s copyright requirements here: PLOS Licenses and Copyright. If you need to request permissions from a copyright holder, you may use PLOS's Copyright Content Permission form.

Please respond directly to this email or email the journal office and provide any known details concerning your material's license terms and permissions required for reuse, even if you have not yet obtained copyright permissions or are unsure of your material's copyright compatibility.

Potential Copyright Issues:

a. Figures 1 and 4: Please confirm whether you drew the images / clip-art within the figure panels by hand. If you did not draw the images, please provide (a) a link to the source of the images or icons and their license / terms of use; or (b) written permission from the copyright holder to publish the images or icons under our CC-BY 4.0 license. Alternatively, you may replace the images with open source alternatives. See these open source resources you may use to replace images / clip-art:

- https://commons.wikimedia.org

- https://openclipart.org/

**Additional Editor Comments (if provided):** **Reviewers' Comments:**

**Comments to the Author**

1. Does this manuscript meet PLOS Digital Health’s publication criteria?

Reviewer #1: Partly

Reviewer #2: Partly

Reviewer #3: Partly

**********

2. Has the statistical analysis been performed appropriately and rigorously?

Reviewer #1: Yes

Reviewer #2: N/A

Reviewer #3: N/A

**********

3. Have the authors made all data underlying the findings in their manuscript fully available (please refer to the Data Availability Statement at the start of the manuscript PDF file)?

The PLOS Data policy

Reviewer #1: No

Reviewer #2: No

Reviewer #3: Yes

**********

4. Is the manuscript presented in an intelligible fashion and written in standard English?

Reviewer #1: Yes

Reviewer #2: Yes

Reviewer #3: Yes

**********

Reviewer #1: This systematic review addresses a critical gap in digital health by focusing on prolonged posture monitoring via accelerometer-based wearable sensors. The study follows the PRISMA guideline and has a registered protocol, which shows the reliability of the methods. However, revisions are needed to improve the clarity of the manuscript.

Introduction

- The definition of “prolonged” is not clear enough. The authors need to specify a time range for prolonged postures (e.g., 10 to 30 minutes) as the focus of this review, and align this definition with guidelines from the Sedentary Behavior Research Network (SBRN). In addition, it is needed to introduce the definitions of prolonged sitting or standing used in previous studies, and compare them to highlight the focus of the current review.

Methods and Results

- The literature search yields over 24k records from 10 databases, but only 23 studies are found to be eligible for inclusion. This large discrepancy may stem from overly broad search terms; for example, “data analysis” is too general and unnecessary for targeting data analysis methods. Thus, the search strategy can be refined to narrow down the relevant records more efficiently.

- While various sensors (e.g., activPAL, ActiGraph) are discussed, their accuracy in monitoring prolonged postures across different populations (e.g., healthy adults, older adults, clinical groups) and settings is not clearly compared. The analytical depth of this review can be improved if the authors use examples from the included studies to support such comparisons.

- Similarly, multiple algorithms (e.g., statistical methods, classical machine learning, deep learning) are used to detect, assess, classify, and predict prolonged postures, but their performance is not quantitatively assessed. The authors may use a more structured way to summarize the performance of different models.

Discussion

- Much of the content in the Discussion section repeats information already presented in the Results. Please avoid restating descriptive findings, and focus on interpreting the results.

- The authors need to provide implications of the current findings for future research and practice in digital health, in particular, how the findings can be used to guide researchers and healthcare practitioners for their future work.

Reviewer #2: a) Overall impression

This manuscript presents a systematic review of accelerometer-based wearable approaches for prolonged posture monitoring, organised as a “sensing-to-feedback” pipeline (data collection → preprocessing → analysis → feedback). The topic is timely for digital health and the pipeline framing is genuinely useful for readers who want to design end-to-end systems rather than isolated classifiers. The review is clearly written and (based on what is provided) follows PRISMA 2020 with a registered protocol in PROSPERO database and supporting files.

However, several issues require attention before the work would fully meet PLOS Digital Health expectations around open science, reproducibility, and complete systematic review reporting. The biggest gaps relate to (i) data/materials availability for the review itself, (ii) full transparency of database-specific search strategies and protocol availability, and (iii) clarity/consistency in reporting (e.g., number of databases, language restrictions).

b) Fit for PLOS Digital Health

Overall fit: good, because the review addresses digital measurement of health-relevant behaviours (wearables, algorithms, and translation to feedback), and explicitly highlights real-world deployment barriers (heterogeneity, limited feedback implementations, privacy considerations).

To align even more tightly with PLOS Digital Health’s publication criteria (rigour, evidence for conclusions, clear utility/accessibility, and appropriate open science practice), the manuscript should strengthen reproducibility and “usable outputs” (share full extracted dataset + structured summary tables + clearer protocol/search transparency). (PLOS Digital Health reviewer guidelines)

c) Major comments

1) Data availability and open-science compliance (high priority)

The Data Availability Statement indicates that only a subset of extracted data is provided, and the full extraction form is “available upon request”.

PLOS policy expects the minimal dataset needed to replicate findings to be publicly available at publication, with restrictions only when ethically/legal necessary. For a systematic review, the extracted data, risk-of-bias judgements, and analysis tables are typically not sensitive and should be shared (e.g., as supporting files or in a repository with a DOI). (PLOS Digital Health data availability policy)

What to change:

• Deposit the complete extraction sheet (all included studies, all extracted fields listed in Table 1) plus the full risk-of-bias scoring per study/domain (not only a figure summary).

• If any element truly cannot be shared, state the specific restriction and a non-author-controlled access mechanism, consistent with PLOS guidance. (PLOS Digital Health data availability policy)

2) Protocol availability and reporting

The manuscript states that the protocol is registered in the PROSPERO registry under registration number (protocol record ID) CRD42025637387. PLOS Digital Health submission guidance for systematic reviews asks authors to indicate whether a protocol exists and, if so, provide it as supporting information and include the registry record number in the abstract. (PLOS Digital Health submission guidelines)

What to change:

• Add the protocol registration number/record ID CRD42025637387 on PROSPERO to the abstract (it currently appears only in the main text).

• Provide the protocol as a supporting file (e.g., exported from PROSPERO database / dated protocol document) and describe any deviations from the registered protocol.

3) Search strategy transparency (database-by-database)

Supporting Information 2 provides the Web of Science strategy and states syntax was adapted for other databases.

For reproducibility, systematic reviews should provide the full strategies (or at minimum the exact final queries) for each database searched, including limits/filters used. PLOS also expects robust, transparent reporting in systematic reviews. (PLOS Digital Health submission guidelines; PLOS Digital Health reviewer guidelines)

What to change:

• Provide complete search strings for all databases listed (PubMed/MEDLINE, EMBASE, CINAHL, SPORTDiscus, IEEE Xplore, ACM, Scopus, ProQuest, etc.), along with the exact run dates for the initial and updated searches.

• The manuscript states the initial search was conducted on 12 January 2025 and that a refined search strategy was re-run on 4 December 2025. This implies the search strings and/or limits were modified between the initial and updated searches, yet only one set of search terms/strategy is provided. Please explicitly report (i) the original search strategy used in January, (ii) the refined strategy used in December, and (iii) a brief description of what was changed and why (e.g., added/removed keywords, revised Boolean logic, altered subject headings, added filters, changes in databases searched). For full reproducibility, provide the exact final queries per database for both runs (or clearly state that the strategy was unchanged and remove “refined” if no refinement occurred). Please also report the number of records retrieved per query and per database for the January search and the December update (before de-duplication), ideally in a supplemental table alongside the exact queries and run dates for each database.

• The authors state that the updated search was not re-run in Engineering Village (EI) because the initial EI results were all duplicates. Given the 11-month interval between the initial search (January) and the update (December), please justify the assumption that EI would again yield only duplicates. Specifically, report (i) the number of EI records retrieved initially and the proportion identified as duplicates, (ii) the basis for concluding EI indexing/coverage relevant to this topic would not change over the update period, and (iii) whether a limited EI update search (or a spot-check) was conducted to confirm no new eligible records were added during those 11 months.

4) Internal consistency: number of databases and language restrictions

• The abstract states that records were identified across nine scientific databases, whereas the Methods list ten databases searched. Please clarify this discrepancy explicitly by stating that ten databases were searched initially, but records contributing to the final set came from nine, because Engineering Village yielded only duplicates and was therefore not re-run (and/or did not contribute unique records after de-duplication). Please ensure the abstract, methods, and PRISMA flow/summary counts are consistent and unambiguous.

• The Methods state that the updated search was re-run with “no restrictions on language”, but the eligibility criteria require full-text articles published in English, and the limitations also state an English-only restriction. Please correct or explain this inconsistency. If the searches were conducted without language limits but inclusion was restricted to English at the screening/full-text stage, state this explicitly and ensure it aligns with the protocol; as the protocol specified English-only, clarify that searching without language limits represents a deviation and, if so, justify it.

What to change:

• Harmonise the database count everywhere (abstract, methods, PRISMA flow narrative).

• Correct the “no language restrictions” statement, or explain that the initial retrieval was unrestricted but inclusion was English-only, and justify why the search was unrestricted with respect to language.

5) PRISMA completeness: excluded full-text studies and reasons

In the PRISMA checklist, item 16b (“cite studies that might appear to meet inclusion criteria, but were excluded, and explain why”) is marked “Not applicable”.

The PRISMA flow diagram appropriately reports the numbers and categories of full-text exclusions (n=126). However, PRISMA checklist item 16b relates to citing excluded studies that might appear to meet inclusion criteria and explaining why they were excluded.

What to change:

• Please either (i) provide a supplementary list of excluded full-text articles (at least the borderline/near-miss papers) with the primary exclusion reason, or (ii) revise the PRISMA checklist entry (16b) to accurately reflect what was (and was not) reported and justify why a citation list was not provided.

6) Risk-of-bias / quality assessment tool needs more operational detail

You created a tailored tool based on QUADAS-AI with five binary domains (0/1).

Supporting Information 3 is currently very high-level (one question per domain) and does not provide sufficient scoring guidance or decision rules, and the manuscript does not report inter-rater agreement (e.g., kappa) for the risk-of-bias assessment.

There is also a narrative tension where reference criteria are described as “often under-reported”, yet “methodological transparency” is scored 100% across studies. This requires clarification of what “methodological transparency” covered and how borderline cases were handled.

What to change:

• Expand the tool into an operational rubric: what counts as “Yes/No” for each domain, examples, and how you handled unclear reporting.

• Provide per-study domain-level ratings in a table (not just aggregated percentages / a heatmap figure).

• Report reviewer agreement and how conflicts were resolved.

7) Strength of conclusions vs. heterogeneity (avoid over-claiming)

You state that deep learning “demonstrated superior performance” and that ML “consistently outperformed” threshold-based approaches.

Given that you did not perform meta-analysis and emphasise substantial heterogeneity, these statements should be qualified carefully (e.g., “in the included comparisons where evaluated”, “in specific datasets/settings”, “limited head-to-head evidence”).

What to change:

• Where making comparative performance claims, point readers to the exact studies and summarise the comparison context (sensor placement, population, ground truth, window length, outcome definition). A structured table of performance outcomes by task would greatly help (even if qualitative).

d) Minor comments

1. Terminology and copy-editing:

• Correct the phrase “with no restrictions language” (missing “on”).

• Ensure consistent spelling of “behaviour/behavior” throughout (journal style may accept either, but consistency matters).

• Consider tightening definitions around “prolonged posture monitoring” vs “sedentary behaviour monitoring” early, and ensure inclusion criteria operationalise that distinction clearly.

2. Support files labelling: Supporting Information 2 is described as the “final complete Web of Science search strategy”; consider retitling it explicitly as “Web of Science strategy (and list of databases searched)” unless you add all database queries.

3. Reproducibility markers: Fig 3 includes elements like “codes available” and “dataset availability” across included studies. Consider defining precisely what counted as “available” vs “by request” and whether this was verified at extraction time.

e) Recommendations

Recommendation to the editor: Major revision.

Rationale: the manuscript is relevant and potentially impactful for PLOS Digital Health, but requires revisions to meet key expectations around open science (share the full extracted dataset and risk-of-bias table), systematic review reproducibility (full database search strings, protocol availability), and consistency/clarity of reporting (database count and language restriction statements). (PLOS Digital Health reviewer guidelines; PLOS Digital Health data availability policy; PLOS Digital Health submission guidelines)

If the authors address the items in Major Comments, the review would be much more reusable to the community and better aligned with PLOS Digital Health criteria on methodological rigour, evidence support, and open science. (PLOS Digital Health reviewer guidelines)

Reviewer #3: I thank the authors for this timely and relevant review addressing prolonged posture monitoring using wearable devices, a topic that has received less attention than sedentary behaviour monitoring in recent years. The manuscript provides a structured synthesis of the literature, including risk of bias assessment, and organises the results along key methodological stages: data collection, preprocessing, analysis, and feedback.

Major Comments:

Mixing prolonged posture and sedentary behaviour monitoring: The authors initially distinguish clearly between these two concepts; however, in the Results and Discussion they are used somewhat interchangeably. If studies on sedentary behaviour were critically evaluated and considered as posture monitoring for the purpose of this review, this should be explicitly justified. A clear separation between sedentary behaviour, including the energy expenditure component (e.g. line 11), and posture monitoring must be maintained. The inclusion criteria (line 160) and exclusion criteria 1.2 (line 204) should not permit the inclusion of sedentary behaviour studies.

Evidence on health effects: Evidence on the negative health effects or relevance of prolonged posture monitoring is limited, most available evidence concerns sedentary behaviour. Please update accordingly.

Scope of included studies: The review includes only studies with substantial contributions to methodological development, including reference criteria (e.g. line 155; Figure 1). Epidemiological studies using wearables are not included. Thus, the manuscript does not review the current state of the art in monitoring, but rather advances in method development. This distinction requires clarification throughout the manuscript.

Results versus discussion: Several sections of the Results already contain interpretations, despite the presence of a separate Discussion section. Interpretative statements should be moved to the Discussion (e.g. lines 371-376, 387-388, 397-399, 427-429, 460-462, 472ff, ...). In addition, many interpretations are not data driven and extend beyond the scope of this review (see also my detailed comments).

Focus of discussion: A substantial part of the Discussion appears to result from not strictly applying the inclusion and exclusion criteria (e.g. lines 738ff, 783ff), as well as from including development and refinement studies in which standardisation is not the primary objective, in contrast to epidemiological studies (e.g. line 744ff). Moreover, the Discussion introduces many additional topics and does not clearly refer back to the Introduction.

Detailed comments

Abstract: how could prolonged posture monitoring go beyond general sedentary behaviour recognition, it is a part of it, no?

Line 3-10: Presented evidence is rather old (2016), partially questionnaire based, for sedentary behaviour (not posture monitoring), and specific for patient population (COPD). Is there more established evidence for healthy people available?

Line 20-21: “researchers are increasingly recognizing the importance of prolonged posture monitoring.” Based on?

Line 23-25: Please clarify “temporal continuity rather than intensity variation.”

Line 31-32: This statement might need a reference.

Line 52-52: Studies vary widely in feedback strategies, but only 2 were included, right?

Line 89-90: “few have examined how methodological decisions across these stages influence real-world deployment or clinical translational potential.” Is there any study who has investigated this topic?

Line 144: What standardized SBRN terminology device names were used?

Line 163: What are insights into? A typical epidemiological study mentions placement and how they process data, but they don’t have a reference criteria. Please elaborate.

Line 197 to 228: There is no need to repeat the opposite of the inclusion criteria as exclusion criteria.

Line 248: I suggest to reference the excluded studies (full-text stage) in the manuscript or an appendix file for the interested reader.

Line 257 ff: I suggest to focus on tools used and not present tools not used.

Figure 3: This is confusing. Please include reference numbers of the manuscript, not the appendix. Same for fig 4

Line 339: activPAL is not an inclinometer.

Line 341-342: Again, sedentary behaviour is not part of this review, please exclude.

Line 359: It is unclear what the authors mean with “As demonstrated in Fig 4, sensor placement varied according to study objectives, device design, and validation strategies.” There is no such information on objectives, design, and validation included in figure 4

Line 366: Pairing it by Bluetooth?

Line 368-370: The added value of this sentence is unclear, no association is presented here.

Entire result section: When talking about the single studies, always reference them for transparency, e.g. line 386-387.

Lien 436-437: these studies should probably not have been included, should they?

Line 451: The sampling rate is not a part of preprocessing

Line 456: Please show the evidence why sampling rate is crucial

Line 468: How dynamic are these posture transitions that they need 50 to 100 Hz?

Line 481: Common but no study included used it?

Line 487: Are denoising and filtering considered independent topics or not? This is unclear.

Line 758-761: Is there evidence for the fact that adherence to wear and ActiGraph is higher than wearing an activPAL?

Line 768: this is not needed for the activPAL, why should it be needed for other sensors?

Line 775: this review is about posture detection, not about asymmetry, and micro-adjustments… see also 805 or 825

Line 777: Is it needed to repeat this information, it is also available from sensor manufacturers?

Line 783: The review is about development studies, what is the expectation? (A major methodological challenge is the lack of standardization in sensor placement)

Line 791: Is there an ask for self-reported reference criteria here?

Line 793ff: not part of this review topic.

Line 808: the focus of this review is on prolonged postures…

Line 813: the mix of intermediate and outcome features is somewhat confusing. It would help if these topics are presented separately.

Line 832: ML is only the default for development studies, epidemiological studies still most often use cut-points, no?

Line 836: performance is no review topic, is it?

Line 862 ff: Why should the reviewed field (studies with reference criteria) implement feedback, a different field might need to be reviewed regarding this topic, no?

Future works: most recommendations are not based on the results of this review, please revise.

Conclusion: most conclusions are not supported by the reviewed studies, please revise.

Line 955: are you asking for standardized calibration study designs?

**********

what does this mean?). If published, this will include your full peer review and any attached files.

**Do you want your identity to be public for this peer review?**  If you choose “no”, your identity will remain anonymous but your review may still be made public.

For information about this choice, including consent withdrawal, please see our Privacy Policy

Reviewer #1: No

Reviewer #2: No

Reviewer #3: **Yes:** Roman P Kuster

**********

**Figure resubmission:**

**Reproducibility:**

To enhance the reproducibility of your results, we recommend that authors of applicable studies deposit laboratory protocols in protocols.io, where a protocol can be assigned its own identifier (DOI) such that it can be cited independently in the future. Additionally, PLOS ONE offers an option to publish peer-reviewed clinical study protocols. Read more information on sharing protocols at https://plos.org/protocols?utm_medium=editorial-email&utm_source=authorletters&utm_campaign=protocols

Attachment Submitted filename: review of paper PDIG-D-26-00054 FINAL.pdf

# Author comment: Author response to Decision Letter 1

**DOI:** 10.1371/journal.pdig.0001538.r002

3 May 2026

Attachment Submitted filename: Rebuttals.pdf

# Peer review: Decision Letter 1

**DOI:** 10.1371/journal.pdig.0001538.r003

**Authors:** Nicole Li-Jessen, Josephine Nanyonjo

15 Jun 2026

A Systematic Review of Prolonged Posture Monitoring With Accelerometer-Based Wearable Sensors

PDIG-D-26-00054R1

Dear Ms Liao,

We are pleased to inform you that your manuscript 'A Systematic Review of Prolonged Posture Monitoring With Accelerometer-Based Wearable Sensors' has been provisionally accepted for publication in PLOS Digital Health.

Before your manuscript can be formally accepted you will need to complete some formatting changes, which you will receive in a follow-up email from a member of our team.

Please note that your manuscript will not be scheduled for publication until you have made the required changes, so a swift response is appreciated.

IMPORTANT: The editorial review process is now complete. PLOS will only permit corrections to spelling, formatting or significant scientific errors from this point onwards. Requests for major changes, or any which affect the scientific understanding of your work, will cause delays to the publication date of your manuscript.

If your institution or institutions have a press office, please notify them about your upcoming paper to help maximize its impact. If they'll be preparing press materials, please inform our press team as soon as possible -- no later than 48 hours after receiving the formal acceptance. Your manuscript will remain under strict press embargo until 2 pm Eastern Time on the date of publication. For more information, please contact digitalhealth@plos.org.

Thank you again for supporting Open Access publishing; we are looking forward to publishing your work in PLOS Digital Health.

Best regards,

Josephine Nanyonjo

Guest Editor

PLOS Digital Health

***********************************************************

**Additional Editor Comments (if provided):**

**Reviewer Comments (if any, and for reference):**

Reviewer's Responses to Questions

**Comments to the Author**

Reviewer #1: All comments have been addressed

Reviewer #2: All comments have been addressed

**********

publication criteria?

Reviewer #1: Yes

Reviewer #2: (No Response)

**********

3. Has the statistical analysis been performed appropriately and rigorously?

Reviewer #1: N/A

Reviewer #2: (No Response)

**********

4. Have the authors made all data underlying the findings in their manuscript fully available (please refer to the Data Availability Statement at the start of the manuscript PDF file)?

The PLOS Data policy

Reviewer #1: No

Reviewer #2: (No Response)

**********

5. Is the manuscript presented in an intelligible fashion and written in standard English?

Reviewer #1: Yes

Reviewer #2: (No Response)

**********

Reviewer #1: I appreciated the authors' effort in significantly revising the manuscript. My comments have been well addressed.

Reviewer #2: (No Response)

**********

what does this mean?). If published, this will include your full peer review and any attached files.

**Do you want your identity to be public for this peer review?**  If you choose “no”, your identity will remain anonymous but your review may still be made public.

For information about this choice, including consent withdrawal, please see our Privacy Policy

Reviewer #1: No

Reviewer #2: No

**********
